# Is inverse probability of censoring weighting a safer choice than per-protocol analysis in clinical trials?

**DOI:** 10.1177/09622802241289559

**Published:** 2024-12-12

**Authors:** Jingyi Xuan, Shahrul Mt-Isa, Nicholas Latimer, Helen Bell Gorrod, William Malbecq, Kristel Vandormael, Victoria Yorke-Edwards, Ian R White

**Affiliations:** 1 MRC Clinical Trials Unit at UCL, University College London, London, UK; 2 Biostatistics and Research Decision Sciences (BARDS) Health Technology Assessment (HTA) Statistics, 2793 MSD, Zurich, Switzerland; 3 Sheffield Centre for Health and Related Research, School of Medicine and Population Health, University of Sheffield, Sheffield, UK; 4 Delta Hat Limited, Nottingham, UK; 5 Department of Mathematics, University of Brussels, Brussels, Belgium; 6 Biostatistics and Research Decision Sciences (BARDS) Health Technology Assessment (HTA) Statistics, 2793 MSD, Brussels, Belgium; 7 Centre for Advanced Research Computing, University College London, London, UK; * Former employee of MSD, Brussels, Belgium throughout most of the duration of this study.

**Keywords:** Inverse probability of censoring weight, per-protocol, non-adherence, treatment switching, dependent censoring

## Abstract

Deviation from the treatment strategy under investigation occurs in many clinical trials. We term this intervention deviation. Per-protocol analyses are widely adopted to estimate a hypothetical estimand without the occurrence of intervention deviation. Per-protocol by censoring is prone to selection bias when intervention deviation is associated with time-varying confounders that also influence counterfactual outcomes. This can be corrected by inverse probability of censoring weighting, which gives extra weight to uncensored individuals who had similar prognostic characteristics to censored individuals. Such weights are computed by modelling selected covariates. Inverse probability of censoring weighting relies on the no unmeasured confounding assumption whose plausibility is not statistically testable. Suboptimal implementation of inverse probability of censoring weighting which violates the assumption will lead to bias. In a simulation study, we evaluated the performance of per-protocol and inverse probability of censoring weighting with different implementations to explore whether inverse probability of censoring weighting is a safe alternative to per-protocol. Scenarios were designed to vary intervention deviation in one or both arms with different prevalences, correlation between two confounders, effect of each confounder, and sample size. Results show that inverse probability of censoring weighting with different combinations of covariates outperforms per-protocol in most scenarios, except for an unusual case where selection bias caused by two confounders is in two directions, and ‘cancels’ out.

## 1. Introduction

Randomised controlled trials (RCTs) are the gold standard for evaluating therapeutic interventions.^1–3^ Intention to treat (ITT), which analyses by randomised treatment assignment is usually of interest and commonly conducted in RCTs.^4^ In a perfect trial where the assignment and receipt of treatment are the same and there are no outcome data missing, the interpretation of trial results is straightforward and ITT analysis provides a valid estimate of the treatment effect.

However, unplanned changes always occur in trials. Patients may discontinue their assigned treatments, take rescue or alternative treatment or switch to another available treatment (either within or outside the trial). Such post-randomisation events are defined as intercurrent events (ICEs) which can ‘influence either the interpretation or the existence of the measurements associated with the clinical question of interest’ and usually occur in clinical trials.^5,6^ ICEs are either violating the pre-specified trial protocol (e.g. non-adherence to assigned amount or schedule of treatment),^7,8^ or permitted by the protocol (e.g. treatment switching after disease progression, especially in oncology trials).^9–11^ Missing data in outcomes caused by loss to follow-up is another issue that occurs commonly. We focus on dealing with ICEs assuming no loss to follow-up throughout this study.

In the presence of ICEs potentially complicating the interpretation of trial results, it is important to clearly specify the research objective(s) and perform the proper analysis to realise the objective(s). The ICH E9 (R1) addendum offered a well-structured framework to translate the research questions into precisely defined estimands, specify statistical analyses targeting each estimand and assess the plausibility of each analysis.^12^

An estimand is defined as ‘A precise description of the treatment effect reflecting the clinical question posed by the trial objective. It summarises at a population level what the outcome would be in the same patients under different treatment conditions being compared’.^12^ According to the addendum, five attributes are required to construct an estimand: treatments, population, ICEs and the strategies to address them, (outcome) variables and population-level summary. Previous papers have provided clear guidance on, and examples of, constructing estimands^13–16^ and we provide more details with an example in human immunodeficiency virus (HIV) in Supplemental Material A. Here we focus on ICEs and it is important to distinguish between ICEs that are considered part of the treatment under investigation and ICEs that are deviations from that treatment.^17^ We define the latter type as an ‘intervention deviation’ (ID). After this classification, according to the framework laid out in the ICH E9 Addendum, we can choose within five strategies including treatment policy strategy, hypothetical strategy, composite variable strategy, while on treatment strategy and principal stratum strategy to deal with each of the ICEs.^12^ For example, when we are interested in the effect of implementing a treatment policy, we will treat the ICEs with the treatment policy strategy ignoring the events occurring after randomisation. The estimand in this case based on treatment policy strategy is called a treatment policy estimand. One common concern regarding the treatment policy estimand is about the external validity (i.e. whether the ICEs occur similarly in practice as in the trial).

Alternatively, we may want to go beyond the treatment policy estimand when there is additional interest in other research questions from other stakeholders or the treatment policy estimand is no longer meaningful when subsequent treatments after the occurrence of ICEs do not reflect clinical practice. Indeed, health technology assessment (HTA) agencies often have an interest in outcomes that would be observed if the new intervention is inserted into the standard treatment pathway, and therefore adjustments need to be made for IDs that are not representative of what would occur in standard clinical practice. This has been shown to be an important factor in analyses considered by healthcare decision-makers based on a review of technology appraisals (TAs) conducted by the National Institute for Health and Care Excellence (NICE).^10^ Also, some patients might want to know what the treatment effect is if they fully adhere to the treatment regimen. To address alternative research questions for better reflection of routine practice or targeting particular interests by some stakeholders, some ICEs can be classified as ID and treated with the hypothetical strategy. In this case, a hypothetical estimand with the treatment attribute being the treatment assigned with the ICEs not classified as ID targets the effect if IDs, possibly including stopping treatment, treatment switching and additional treatments, had not occurred. A hypothetical estimand needs to be precisely defined considering the trial setting and the routine practice and classifying the ICEs into IDs and others require careful consideration regarding the characteristics of the ICEs. IDs can be defined according to the reasons for, the time of their occurrence and the extent of the deviation and in turn contribute to different hypothetical estimands.^18^

With a well-defined estimand, we can then design statistical analyses to obtain reliable estimates. The ICH E9 (R1) addendum suggests that the choice of analysis should align with the estimand of interest and the assumptions required by the estimators for analysis should be justifiable.^12^ For a treatment policy estimand, researchers generally use ITT analyses, or else perform common statistical analyses without regard to ICEs. When targeting a hypothetical estimand, per-protocol (PP) is usually performed by exclusion or censoring.^19^ We prefer censoring rather than exclusion since the latter wastes the data from before the occurrence of ID and is also subject to immortal time bias. PP can only provide a valid estimate when the ID occurs independent of prognostic characteristics (i.e. censoring is non-informative).^20^ However, baseline demographic characteristics, and time-varying covariates (TVCs) such as indicators of disease severity, disease progression and prognostic biomarkers are often important factors influencing the occurrence of ID. Thus, participants who have and do not have ID generally differ in prognostic characteristics (i.e. censoring at ID is informative). In addition, in non-inferiority (NI) trials, even without the estimand explicitly defined, the common belief that ITT is anti-conservative (i.e. prone to reject the null) leads to the frequent use of PP.^21,22^ But anti-conservatism cannot be evaluated without the estimand being clearly defined so the simple choice of PP over ITT in such cases is questionable. Given that PP is likely to be problematic when targeting a hypothetical estimand, it has been suggested that we should go beyond PP.^19^

To account for the selection bias caused by the informative censoring in PP analysis, one simple method is to further adjust for the covariates by simple multivariate regression to recover the balance broken by the dependent censoring. This is only valid (with no unmeasured confounders) where confounders are not time-dependent. In a time-dependent setting with TVCs, simple regression methods will provide biased estimates due to over-adjusted confounders because the confounders may themselves be affected by treatment (i.e. confounders are also mediators).^23,24^

The development of Robins’ G-methods offers a path to obtaining valid estimates in the presence of time-varying post-randomisation factors potentially causing time-varying confounding.^25^ Inverse probability of censoring weighting (IPCW) is one of the G-methods to estimate a hypothetical estimand by censoring and giving weight computed on the basis of the similarity in prognostic characteristics between participants who have been censored and participants who have not yet been censored. Commonly used statistical methods for outcome analyses can then be implemented with their weighted version.^26,27^ We see the IPCW method as an elaborated version of the simple PP analysis and we note that the IPCW method (as defined in this article) was termed as a per-protocol approach using IPCW in a recent paper.^28^ Other adjusting methods are also available and the European Medicines Agency (EMA) has published guidance on the methods including IPCW, rank preserving structural failure time models (RPSFTM) and two-stage method (TSM).^29^ It was mentioned in ICH E9 (R1) that the analysis of hypothetical estimands requires the counterfactual outcomes which are not observed but the addendum does not cover causal analysis methods.^12^ In practice, IPCW has been pre-specified as an analysis method in the statistical analysis plan (SAP) and adopted in the analysis in several NICE TAs.^30,31^ Though some methodological papers have explored the use of IPCW in trials,^32–36^ IPCW remains uncommon in trials considering ID. IPCW may not have been adopted more widely due to concerns about the NUC assumption being untestable and a lack of implementation guidance.

The aim of this article is to choose a suitable method targeting a hypothetical estimand, taking the perspective of writing a SAP, that is, of planning an analysis before seeing the data. We aim to investigate whether IPCW is a safer alternative to PP when targeting hypothetical estimands through simulation by assessing and comparing the performance of PP and IPCW with correct or potential suboptimal implementations in a series of scenarios.

The article is structured as follows. In Section 2, we give an introduction to the methods under investigation. In Section 3, we describe the simulation study designed to compare the performance of methods under a series of scenarios. In Section 4, we present the results of our simulation. In Section 5, we provide a brief case study illustrating the application of the ideas presented. Finally, in Section 6, we discuss the strengths, limitations and future exploration directions of this study as well as give recommendations for analyses in trials.

## 2. Overview of methods

We focus on parallel-group, randomised, controlled clinical trials with ICEs. We assume longitudinal data with time-updated measurements of TVCs and time-to-event outcomes. For simplicity, we assume that only one type of ICE is classified as ID. This setting is actually quite general because when there are several ICEs defined as IDs all handled by the hypothetical strategy we can combine them into one ICE type and apply adjustment methods. The ID occurring over time is caused by baseline and time-varying confounders (which are influenced by the occurrence of the ID and have a causal effect on counterfactual outcomes). Our target is a hypothetical estimand if ID had not occurred. In this section, we define notations, provide a brief review of the methods under investigation and give a summary of the performance of methods taking the theoretical assumptions and potential implementations of each method in practice into consideration.

### 2.1. Notation

Let uppercase letters represent the random variables, lowercase letters represent the realisations of corresponding random variables or constants, Greek letters represent the unknown parameters and overbars represent the histories.

We use subscripts i to denote the values of the variables for patient i , labelled $i=1,\ldots ,n_{obs}$ . We use subscripts v to represent baseline and follow-up visits, labelled v=0,1,…,V where v=0 denotes the baseline and v=V denotes the last visit. Counterfactual variables are indexed with a superscript.

-
$B_{i}$ denotes the set of baseline (time-independent) covariates that are associated with both the occurrence of ID and the counterfactual outcomes.
-
$L_{iv}$ is the set of TVCs including three sets of covariates: $LC_{iv}$ (time-dependent confounders associated with both the occurrence of ID and the counterfactual outcome), $LR_{iv}$ (outcome risk factors that don’t predict ID) and $LI_{iv}$ (ID risk factors that do not predict outcome).
-
$Z_{i}$ is the binary variable for treatment allocation for participant i with 0 denoting the control group and 1 denoting the experimental group.
-
$C_{iv}$ is the binary indicator for the occurrence of ID ( C for censoring) for participant i at visit v where v=0 (baseline), 1,2,… with 0 denoting the absence of ID and 1 denoting the presence of ID. $C_{ivto}=\sum_{t=1}^{v}C_{it}$ denotes the presence of ID up until visit v .
-
$A_{iv}$ is the variable for treatment actually received for participant i at visit v . $A_{iv}$ takes a value in the set $A_{v}(Z\in A_{v})$ where different values represent different treatment benefits of subsequent treatment. Treatment received at v=0 is $A_{i0}=Z_{i}$ .
-
${\bar{A}}_{vi}=A_{0i},\ldots ,A_{vi}$ denotes the treatment history up to visit v for participant i . ${\bar{A}}_{i}$ is the treatment history of participant i during the trial. In particular, ${\bar{a}}_{0}=(0,0,0,\ldots )$ denotes the treatment regimen for the control group that randomly assigned treatment is received during the trial period without ID. Similarly, ${\bar{a}}_{1}=(1,1,1,\ldots )$ denotes the treatment regimen for the experimental group.
-
$T_{ivon}$ denotes the number of periods (intervals between visits) spent on treatment for participant i until visit v .
-
$T_{ivoff}$ denotes the number of periods spent off treatment for participant i until visit v .
-
$Y_{iv}$ denotes the outcome variable for participant i at visit v .
-
$Y_{iv}^{\bar{a}}$ denotes the potential outcome of participant i under treatment history $\bar{a}$ .

### 2.2. Intention to treat

ITT analysis attempts to estimate a treatment policy estimand by ignoring ID. ITT analysis compares treatment groups as randomly assigned and represents a valid comparison of randomised groups rather than estimating a hypothetical estimand.

### 2.3. Per-protocol

PP analysis is performed by simply censoring the occurrence of ID or excluding participants with ID. We only consider PP by censoring since PP by exclusion is thought to be indefensible in the presence of time-dependent IDs. The validity of estimates yielded by PP depends on the plausibility of its ‘non-informative censoring’ assumption. When the ID is not associated with the prognostic characteristics, the balance of prognostic factors by randomisation remains (i.e. the censored participants and uncensored participants are comparable regarding the prognostic factors). Such PP analysis by non-informative censoring will provide an unbiased estimate of the hypothetical estimand. However, when the ID is caused by confounders, the balance by randomisation is broken and PP censoring is now informative since it carries information about the prognosis of participants (i.e. the censored and uncensored participants are not comparable). In this case, the estimate obtained by PP analysis is subject to selection bias.^37–39^

### 2.4. Inverse probability of censoring weighting

IPCW works by censoring at ID and giving weights to uncensored participants to recover the broken balance of prognostic characteristics by censoring. The weight is the inverse of the probability of remaining uncensored given the values of both baseline and time-varying confounders. The estimation process of IPCW can be divided into two steps including modelling the weight and modelling the outcome.

First, with a set of baseline covariates $B_{i}$ and a set of TVCs $K_{iv}$ being a subset of L representing the selected covariates for analysis (which may actually be confounders only, non-confounding factors only, or both included), we fit a model by pooled logistic regression:

(1) $$logitP(C_{iv}=1|C_{i(v-1)}=0,B_{i},K_{iv})=\eta B_{i}+\zeta K_{iv}+\iota f(v)+\vartheta Z_{i}$$

where f(v) is a function of visit v .

After that, we predict the probability of occurrence of ID for individual i at visit v :

(2) $${\hat{p}}_{iv}=\hat{P}[C_{iv}=1|C_{i(v-1)}=0,B_{i},K_{iv}]$$

Then the unstabilised weight of individual i at visit v can be constructed by multiplying together all their previous and current probabilities of not having incurred ID up to visit v :

(3) $${\hat{W}}_{iv}=\prod_{k=1}^{v}\frac{1}{1-{\hat{p}}_{iv}}$$

Alternatively, stabilised weights which are recommended to improve the precision can be implemented by changing the numerator from 1 in equation (3) into the probability of a participant not having ID at visit v given that the participant was uncensored at last visit v−1 , conditional on treatment randomly assigned.^40^ First fit the model without $K_{iv}$ and $B_{i}$ and then obtain a numerator:

(4) $${\hat{p}}_{0iv}=\hat{P}[C_{iv}=1|C_{i(v-1)}=0]$$

Then obtain the stabilised weight by the following equation:

(5) $${\hat{SW}}_{iv}=\prod_{k=1}^{v}\frac{1-{\hat{p}}_{0iv}}{1-{\hat{p}}_{iv}}$$

After the weighting process, we can then perform the common methods in survival analysis including the Kaplan-Meier (KM) estimator, log-rank test, and Cox partial likelihood estimator of the ratio of the treatment–arm-specific mortality rates by their IPCW versions.^41,27^

Some assumptions are required for obtaining reliable estimates by IPCW. First, the performance of the IPCW method heavily relies on its NUC assumption whose plausibility is not statistically testable.^42,43^ Second, the models for ID and outcome should be correctly specified.^44^ Furthermore, the positivity assumption is required that there should not be participants who are certain to have ID.^45,44^ Measurement errors are another source of bias.

### 2.5. Summary of methods

Given the assumptions required by each method, we further consider sample size, data structure of covariates, ID and outcome and suboptimal implementations of methods potentially leading to bias (summarised in Table 1). Here we assume correct specifications (with no non-linear terms and no interaction terms between confounders and the treatment) of outcome and ID models. Unless the ID has no influence on the treatment effect, an ITT analysis targeting the treatment policy estimand generally provides different estimates of the treatment effect compared to an analysis that addresses a hypothetical estimand. PP analyses are subject to selection bias when the censoring is informative. IPCW with residual selection bias caused by violation of NUC can occur when adjusting for too few or mis-specified covariates, with missing data or insufficient measurement frequency in covariates or a mis-specified ID model. No measurements on a variable (e.g. omitting a variable) can be seen as an extreme case of missing measurements of a variable (e.g. missing data or insufficient frequency in the measurement of a variable). When the trial has a small sample size or ID with less vague criteria leading to certain ID occurrences in some participant groups, the positivity assumption is likely to be violated, because uncensored participants are unlikely to be representative of censored participants. Stochastic non-positivity can occur in trials with small sample sizes and deterministic non-positivity is likely to happen when ID is determined by the values of certain variables (deterministic ID). When covariates are measured at the wrong time or data for IDs are measured with errors, IPCW can also yield biased estimates.

**Table 1.** Potential source of bias by each method targeting a hypothetical estimand.

Method implementation			Assumption violated	Source of bias
***ITT***				
Ignoring ID			–	Difference in estimands
***PP***				
Censoring at ID			Non-informative censoring	Selection bias
***IPCW***				
Censoring at ID	+	No adjustment for selection bias	NUC	Selection bias (same as PP)
		Adjustment with too few covariates	NUC	Residual selection bias
		Adjustment with mis-specified covariates	NUC	Residual selection bias
		Adjustment with missing data in covariates	NUC	Residual selection bias
		Adjustment with insufficient covariate	NUC	Residual selection bias
		measurement frequency		
		Adjustment in a small sample	Positivity	Small sample bias
		Adjustment for deterministic ID	Positivity	Strong selection bias
		Adjustment with data measured with errors	No measurement error	Measurement bias

ITT: intention to treat; ID: intervention deviation; PP: per-protocol; IPCW: inverse probability of censoring weighting; NUC: no unmeasured confounding.

## 3. Simulation study

In this section, we introduce the simulation conducted to uncover the performance of each method in estimating a hypothetical estimand. The simulation study is informed by ODYSSEY (a trial in paediatric HIV infection) for a realistic trial setting.^46^ Our simulation refers to the properties of the TVCs, IDs and the outcome in the trial. We design the ID mechanism with higher prevalence and stronger selection bias in the base case scenario and vary the ID and outcome mechanism to accommodate wider trial settings. The design of the simulation is described using the ADEMP framework by Morris et al.^47^

### 3.1. Aim

The overall aim of this study is to know whether IPCW with different implementations that are likely to happen in practice (commonly manifesting as including correctly-specified, too many, too few or mis-specified covariates) can consistently outperform PP across a range of scenarios. Implementation of IPCW with too many covariates is realised by including additional non-confounding variables; with too few covariates is realised by omitting one of the confounders; and with mis-specified covariates is realised by including non-confounding covariate(s) and omitting a confounder. IPCW with measurement error in data is realised by adjusting for TVCs which are measured with delay at each visit. We focus on addressing the following five questions:

1. How does IPCW perform when confounders are correctly specified?
2. How does IPCW perform when there is residual confounding?
3. How does IPCW perform when unnecessary TVCs (non-confounding variables) are adjusted for?
4. How does IPCW perform when TVCs are measured with error?
5. Is IPCW with different confounder choices usually less biased than PP?

### 3.2. Data-generating mechanisms

**Figure 1.**
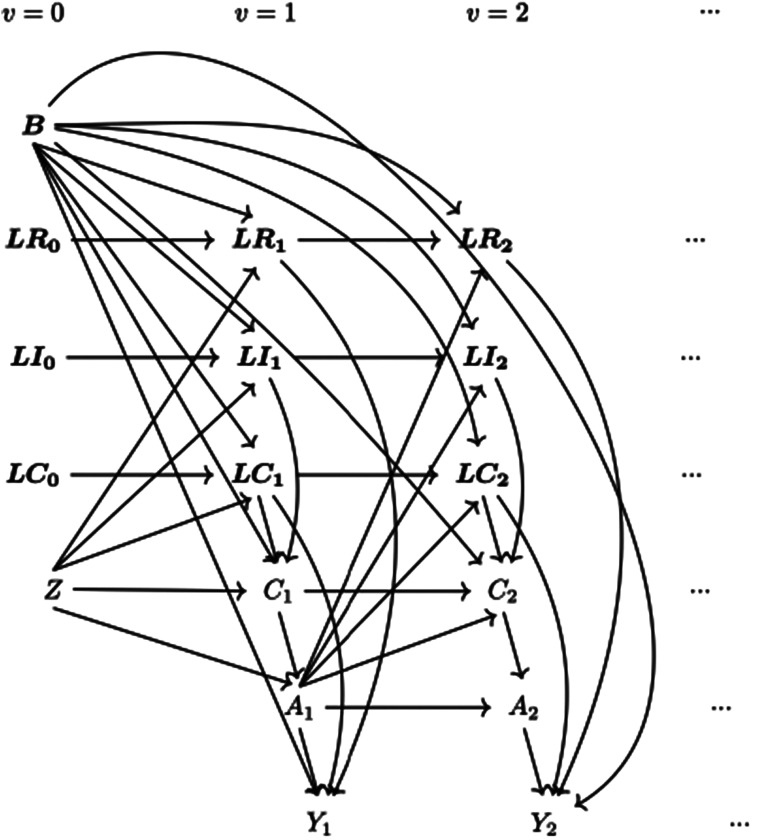
Causal directed acyclic graph (DAG) for data-generating mechanism. *Note:* (1) Suppressing i for the variables shown in Section 2.1, $LR_{v}$ , $LI_{v}$ and $LC_{v}$ are used to represent generic realised risk factors, intervention deviation factors, and confounders at visit v . (2) For longitudinal data measured at multiple visits, separate nodes are used for each variable at each time point to avoid cycles. (3) A is included in the DAG for better illustration but not included in the data-generating process.

Data-generating mechanisms are described in this section. Following a supplement in notations, we start with the framework for the simulation and then give mathematical details for the data generation of each variable. The data-generating process is depicted by a directed acyclic graph (DAG) (see Figure 1) as well as a detailed algorithm (see Supplemental Material B.1). Considering each part of the data-generating mechanism, we identified factors to be varied and designed scenarios to accommodate multiple settings for the exploration of the methods of interest.

We build on the notations described in Section 2.1, here we add further details. The vector of baseline covariates $B_{i}$ has three components: baseline age ( $B_{i1}$ ), gender ( $B_{i2}$ ) and WHO stage ( $B_{i3}$ ). The vector of TVCs measured at each visit v denoted as $L_{iv}$ has five components including logarithm of CD4 count ( $L_{iv1}$ ), logarithm of CD4/CD8 ratio ( $L_{iv2}$ ), viral load ( $L_{iv3}$ ), total cholesterol ( $L_{iv4}$ ) and triglycerides ( $L_{iv5}$ ). Therefore, sets of time-varying confounders, ID factors and outcome risk factors are $LC_{iv}=\{L_{iv1},L_{iv2}\}$ , $LR_{iv}=\{L_{iv3}\}$ and $LI_{iv}=\{L_{iv4},L_{iv5}\}$ .

#### 3.2.1. Data-generation process

The simulating process is designed in a setting where $n_{obs}$ participants enter the trial at baseline ( v=0 ) with baseline covariates $B_{i}$ measured and are randomly assigned to intervention $Z_{i}$ . Then at each of the following visits, data are simulated in a sequential conditional manner with TVCs ( $L_{iv}$ ), intervention received ( $A_{iv}$ ) and outcome status ( $Y_{iv}$ ) at each visit generated in turn conditional on the previous values, starting from the baseline visit. We show the causal ordering of two post-randomisation visits in Figure 1 where data are generated from left to right following time sequence and from top to bottom following the details of each variable.

1. Baseline measurement model. Values of covariates except for $B_{i2}$ at v=0 are generated from a truncated multivariate normal distribution. $B_{i1},B_{i3},L_{i01},L_{i02},L_{i03},L_{i04},L_{i05}∼TruncNorm(\mu_{0},\Sigma ,m,n)$ . Then the binary $B_{i2}$ is generated from $B_{i2}∼Bernoulli(logit^{-1}(\rho_{1}B_{i1}+\rho_{2}B_{i3}+\rho_{3}L_{i0}))$ .
2. Treatment allocation (randomisation) model. Treatment assignment is generated using $Z_{i}∼Bernoulli(0.5)$ which denotes a 1:1 randomisation to the control and the experimental group.
3. TVC model. The values of TVCs are simulated visit by visit depending on treatment history, visit and covariates history. The values of TVCs are generated at each follow-up visit v :
(6) $$\begin{array}{rl} L_{ivq} & =\beta_{q0}+\beta_{q1}Z_{i}+(\beta_{q2}+\beta_{q3}Z_{i})C_{i(v-1)to}+\beta_{q4}B_{i}+\beta_{q5}L_{i(v-1)q}+\epsilon_{iq}\\ & \;\epsilon_{i}∼Norm(\xi ,\Lambda ) \end{array}$$
  where v=1,…,8 , $i=1,\ldots ,n_{obs}$ and q=1,…,5 . $\epsilon_{i}$ is the vector of error terms for $L_{ivq}$ with mean vector ξ and covariance matrix (for q=1,2 ) Λ . $L_{1}$ and $L_{2}$ are made correlated by the error terms $\epsilon_{i}$ , rather than by letting each L depend on lagged values of other L ’s. The correlated error terms for each participant at each visit are realised by using Cholesky factorisation. The correlation matrix M of the error terms of time-dependent confounders is fixed across time. Non-confounding covariates ( q=3,4,5 ) are simulated independently.
4. ID model. Let $p_{iv}$ denote the probability of ID for participant i in the interval [v,v+1) . The indicator of ID for participant i at visit v is generated:
(7) $$\begin{array}{rl} p_{iv} & =logit^{-1}(\theta_{0}+\theta_{1}v+\theta_{2}Z_{i}+\theta_{3}B_{i}+\theta_{4}L_{iv})\\ & \;C_{iv}∼Bernoulli(p_{iv})\\ & \;if\;C_{i(v-1)}=0 \end{array}$$
$\theta_{3}$ is a vector of parameters for baseline covariates and $\theta_{3}=(\theta_{31},\theta_{32},\theta_{33})$ . $\theta_{4}$ is a vector of parameters for time-dependent covariates and $\theta_{4}=(\theta_{41},\theta_{42},\theta_{43},\theta_{44},\theta_{45})$ . Note that time-dependent covariates in the ID model include two confounders ( $L_{1}$ and $L_{2}$ ) and two non-confounding factors ( $L_{4}$ and $L_{5}$ ) associated with ID. Non-confounding risk factor ( $L_{3}$ ) is not included in the model since it is not associated with ID ( $\theta_{43}=0$ ). ID can occur either in the control arm only or in both arms.
5. Outcome model. In ODYSSEY, the primary endpoint is treatment failure by 96 weeks which is tested until week 96. For simplicity, we assume discrete time of outcome which is a close approximation to time to event data. We work with the probability of failure in a single time interval [v,v+1) given survival up to visit v , as the discrete equivalent of the hazard function. Let $\lambda_{iv}$ denote the probability of failure in the interval [v,v+1) conditional on survival up to visit v ,
(8) $$\begin{array}{rl} logit(\lambda_{iv}) & =\mu_{0}+\mu_{1}Z_{i}+(\mu_{2}+\mu_{3}Z_{i})T_{ivon}+(\mu_{4}+\mu_{5}Z_{i})T_{ivoff}+\mu_{6}B_{i}+\mu_{7}L_{iv}\\ & \;Y_{iv}∼Bernoulli(\lambda_{iv}) \end{array}$$
  where v=1,2,…,8 , $i=1,2,\ldots ,n_{obs}$ , $T_{ivon}=min(v,T_{ioff})$ and $T_{ivoff}=max(v-T_{ioff},0)$ . Time-dependent non-confounding covariates $L_{4}$ and $L_{5}$ are not included in the outcome model since they are not associated with the outcome ( $\mu_{74}=\mu_{75}=0$ ).

#### 3.2.2. Scenario design

Referring to the ODYSSEY trial data, some parameters are designed with fixed values in the models in the DGM which are shown in Supplemental Material B.2.

We first set a base case with a medium correlation between $L_{1}$ and $L_{2}$ , control-arm ID with medium prevalence, a medium magnitude of confounder on ID, the same sign of the effect of $L_{1}$ on ID and $L_{2}$ on ID (shown in Figure B1(a), a medium effect of arm on outcome, a medium effect of confounder on the outcome and a sample size 1000 (the choice of setting for the base-case scenario is shown in bold in Table 2).

**Table 2.** Summary of factors varied in models.

Factor	Variation	Expected effect
***TVC model***		
Correlation between $L_{1}$ and $L_{2}$	Low/**medium**/high	Residual selection bias
Between-arm difference in effect of ID	**NA**/0/ − / +	Post-ID treatment effect
***ID model***		
Arm of ID occurrence	**Control**/both	Total selection bias
Prevalence of ID	Low/**medium**/high	Total selection bias
Effect of treatment-ID	**NA**/0/ − / +	Direct effect on ID
Magnitude of confounder-ID	Low/**medium**/high	Total & residual selection bias
Sign of confounder-ID	$L_{1}\;L_{1}$ - $L_{2}\;L_{2}$ -/ $L_{1}$ - $L_{2}$ +	Total & residual selection bias
Deterministic ID	**No**/yes	Strong selection bias
***Outcome model***		
Effect of arm on outcome	Low/**medium**/high	Direct treatment effect
Effect of confounder on outcome	Low/**medium**/high	Total & residual selection bias
Between-arm difference in effect of ID	**NA**/0/ − / +	Post-ID treatment effect
***Setting***		
Sample size	200/500/**1000**	Small sample bias

*Note:* TVC: time-dependent covariates; ID: intervention deviation. Choice of settings in the base-case scenario (Scenario 1) is shown in bold. Since the base-case scenario has single-arm intervention deviation, arm-related factors are irrelevant and noted as NA. The between-arm difference in the effect of ID (Arm Diff) is the difference between the control and experimental groups in the effect of intervention deviation on covariates and outcome.

We attempt to cover different trial settings with different characteristics potentially violating the assumption(s) of each method under investigation as shown in Table 1. To realise this, we vary the factors shown in Table 2.

For the time-dependent confounder model, we varied the correlation between $L_{1}$ and $L_{2}$ to accommodate different expected effects of missing one of the confounders. We expect a smaller difference by omitting one confounder compared with NUC in the case where two confounders are highly correlated than in the case where two confounders are less correlated. The between-arm difference in the effect of ID is additionally explored apart from the main aims since it does not affect the performance of censoring methods including the PP and IPCW but only affects the performance of ITT analysis.

The ID model is designed to emulate the case in practice where participants with worse prognoses are more likely to have ID. We varied the arm of ID occurrence, the prevalence of ID, the effect of the arm on ID, and magnitude and the sign of the effect of confounder on ID. By varying the magnitude and sign of the effect of confounder on ID, the confounding caused by each confounder and the confounding in total are varied (edge $C\leftarrow L_{1}$ and edge $C\leftarrow L_{2}$ in Figure B1 in Supplemental Material B).^48,49^

The effect of arm on outcome and the effect of confounder on outcome are considered in the outcome model. These factors influence the event rate as well as the confounding caused by confounder (edge $L_{1}\to Y$ and edge $L_{2}\to Y$ in DAG in Figure B1 in Supplemental Material B). Similar to the between-arm difference in the time-dependent confounder model, the between-arm difference in the effect of ID is included here only for additional analysis that investigates the performance of ITT analysis.

While most scenarios are designed with a sample size of 1000, we also included scenarios with sample sizes of 200 and 500 to investigate small sample bias and imprecision.

Following the identified factors introduced above, this simulation is implemented in a one-by-one exploration process with 18 main scenarios explained in this section and four additional scenarios shown in Supplemental Material B. We show details for scenarios in Table 3 for main scenarios and in Supplemental Material B.2 for other scenarios.

**Table 3.** List of scenarios with varying factors, as described in Table 2. Base-case is Scenario 1, with whom the other scenarios are to be compared by changing one factor at a time.

	TVC model		ID model						Outcome model			
						Confounder-ID					
No.	Corr ( $L_{1}$ , $L_{2}$ )	Arm Diff	Arm(s)	ID prevalence	Arm-ID	Magnitude	Sign	Deterministic ID	Arm -outcome	Confounder -outcome	Arm Diff	Sample size
**1**	**Medium**	**NA**	**Ctrl**	**Medium**	**NA**	**Medium**	−/−−/−	**No**	**Medium**	**Medium**	**NA**	**1000**
2	**Low**	NA	Ctrl	Medium	NA	Medium	−/−	No	Medium	Medium	NA	1000
3	**High**	NA	Ctrl	Medium	NA	Medium	−/−	No	Medium	Medium	NA	1000
4	Medium	NA	Ctrl	**Low**	NA	Medium	−/−	No	Medium	Medium	NA	1000
5	Medium	NA	Ctrl	**High**	NA	Medium	−/−	No	Medium	Medium	NA	1000
6	Medium	NA	Ctrl	Medium	NA	**Low**	−/−	No	Medium	Medium	NA	1000
7	Medium	NA	Ctrl	Medium	NA	**High**	−/−	No	Medium	Medium	NA	1000
8	Medium	NA	Ctrl	Medium	NA	Medium	−/+−/+	No	Medium	Medium	NA	1000
9	Medium	NA	Ctrl	Medium	NA	Medium	−/−	**Yes**	Medium	Medium	NA	1000
10	Medium	**0**	**Both**	Medium	**0**	Medium	−/−	No	Medium	Medium	**0**	1000
11	Medium	**0**	**Both**	Medium	−−	Medium	−/−	No	Medium	Medium	**0**	1000
12	Medium	**0**	**Both**	Medium	++	Medium	−/−	No	Medium	Medium	**0**	1000
13	Medium	NA	Ctrl	Medium	NA	Medium	−/−	No	**Low**	Medium	NA	1000
14	Medium	NA	Ctrl	Medium	NA	Medium	−/−	No	**High**	Medium	NA	1000
15	Medium	NA	Ctrl	Medium	NA	Medium	−/−	No	Medium	**Low**	NA	1000
16	Medium	NA	Ctrl	Medium	NA	Medium	−/−	No	Medium	**High**	NA	1000
17	Medium	NA	Ctrl	Medium	NA	Medium	−/−	No	Medium	Medium	NA	**200**
18	Medium	NA	Ctrl	Medium	NA	Medium	−/−	No	Medium	Medium	NA	**500**

*Note:* TVC : time-varying covariates; ID: intervention deviation; Corr: correlation; Diff: difference; Ctrl: control; Arm: treatment arm. The line with all factors bold shows the settings for the base-case (Scenario 1). For each scenario, the changed factor setting is shown in bold.

### 3.3. Estimand

#### 3.3.1. Causal effect of interest

Given the time-dependent setting with sustained treatment, we define the interventions under investigation as the treatment history received ( $\bar{A}$ ) as a whole during the trial. Since our research interest is the hypothetical estimand without the occurrence of ID, we are comparing two treatment regimens ${\bar{a}}_{0}$ and ${\bar{a}}_{1}$ . The causal effect of interest is then defined by comparing counterfactual outcomes $Y_{v}^{{\bar{a}}_{0}}$ and $Y_{v}^{{\bar{a}}_{1}}$ .

#### 3.3.2. Choice of effect measure

We use the cumulative incidence difference at 96 weeks ( v=8 ) as an effect measure to compare counterfactual outcomes in two arms:

(9) $$RD(v)=Pr(Y_{v}^{{\bar{a}}_{1}}=1)-Pr(Y_{v}^{{\bar{a}}_{0}}=1)$$

RD(96) is the difference in the proportion of failing (meeting primary endpoint) in each arm at any visit up to the week 96 ( v=8 ) censoring date, representing a treatment effect in a randomised controlled trial similar to ODYSSEY.

#### 3.3.3. True value of the estimand

In this simulation, the true value of the estimand (risk difference) is not known directly: it does not appear in the data-generating mechanism. An alternative solution is to estimate the estimand from a large simulated dataset.^50^ To obtain the ‘true’ value of the estimand through simulation, we should set a sufficiently large dataset where the variance of the estimand is negligible. The ‘true’ risk difference was designed to be obtained by simulating the counterfactual treatment by always simulating the regimen without any occurrence of ID with a large sample size of $2\times 10^{6}$ .

### 3.4. Methods of analysis

#### 3.4.1. Handling ID

In the presence of ID, we consider ITT (since it is commonly implemented in RCTs), PP and IPCW methods (Table ). Based on different implementations potentially violating the assumptions of the methods as stated in Table 1, we designed multiple forms of inverse probability weighting model including IPCW with NUC, residual confounding and mismeasured confounders to represent realistic cases where IPCW can go wrong. IPCW with missing data or insufficient frequency of measurements in TVCs are not included as we expect them to be a special form of missing TVCs. IPCW with NUC is expected to provide an unbiased estimate of the estimand since the model is correctly specified while the other implementations violating the NUC are expected to provide biased estimates. IPCW with unnecessary covariates is added since it is a situation likely to occur in practice, potentially influencing the standard error (SE), though not expected to increase bias. All covariates are kept in the model as specified regardless of the statistical significance to reflect a structural way of confounder selection which is based on pre-specified covariates rather than a data-driven method.^51,52^

**Table 4.**
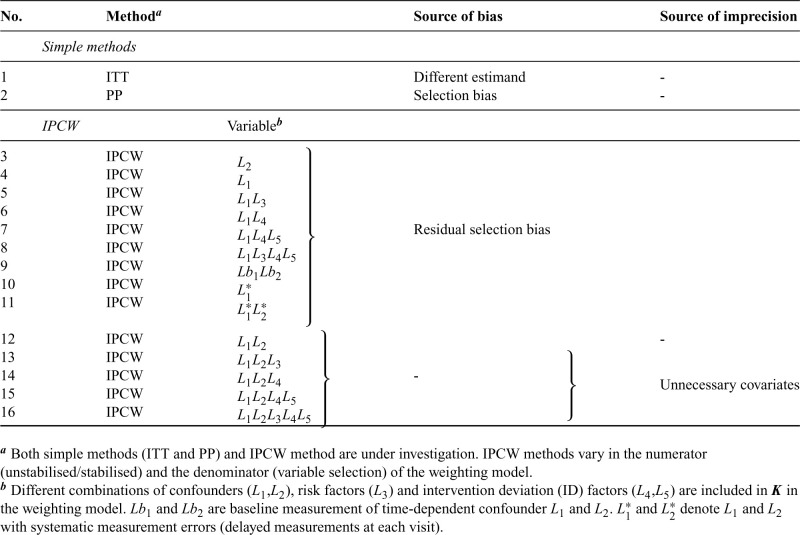
Analysis methods under investigation.

#### 3.4.2. Modelling outcome

With binary outcomes measured across follow-up visits, the outcome is modelled by pooled logistic regression (weighted version for IPCW).

(10) $$logit\lambda (Y_{iv}=1|Y_{i(v-1)}=0,C_{iv}=0)=\alpha Z_{i}+\gamma v+\psi Z_{i}v$$

Since our ultimate estimand is risk difference, the cumulative incidence in each arm and then their difference is calculated with SE by delta method after fitting the logistic model.

#### 3.4.3. Summary of method application

ITT is performed by fitting the outcome model (10) with all the participants.

PP is performed by censoring participants at the occurrence of ID and then fitting the outcome model (10) in the censored population.

The weighting process for IPCW is implemented as described in Section 2.4 but with a different TVC set $K_{iv}$ chosen as specified in Table . Then the outcome is modelled by (10) using weighted population computed with weight=W (3) or weight=SW (5).

### 3.5. Performance measures

Five performance measures: absolute bias, empirical standard error (EmpSE), model-based standard error (ModSE), root mean squared error (RMSE) and coverage (%) of the 95% confidence interval (CI) are evaluated. The pursuit of less biased estimates is the primary concern when evaluating the performances of the methods considering too few or mis-specified covariates. On the other hand, in the case of too much (unnecessary) adjustment, effect estimates are likely to be unbiased, and the focus turns then to the effect of adjustment on precision. We also report the Monte Carlo standard error (MCSE) for these performance measures to quantify the uncertainty of the simulation considering our chosen number of repetitions.

### 3.6. Implementation

#### 3.6.1. Retrieving model-based standard error

The model-based standard error was calculated through the delta method and a two-sided 95% CI was obtained using Normal theory for the cumulative incidence in all the scenarios. Bootstrapping was also considered to obtain non-conservative CIs. Given the large number of scenarios considered, and the fact that each of these required 1000 simulations, it was not possible to perform bootstrapping for every one of these. Therefore we ran Scenario 1 with two forms of IPCW to see the degree of difference in SE and coverage provided by delta and by bootstrapping.

#### 3.6.2. Handling of random number seeds

For all scenarios with varying data-generating mechanisms, the same starting seed was used so that results are correlated within each repetition.

To avoid possible overlap between the states that we use for generating random numbers in simulations aiming at exploring our aims, and the states that we use to simulate large datasets for the sake of obtaining the ‘true’ value of RD, separate streams are used for the random numbers for main exploration and truth calculation.^47^

#### 3.6.3. Software

The simulation study was performed in Stata software, version 16.0. Performance measures were computed by simsum.^53^ The visualisation of the performance measures was realised by siman in stata.^54^

## 4. Results

In this section, we present 18 main scenarios as described in Table 3 and we provide a summary of characteristics of the simulated datasets in Table C1 in Supplemental Material C. Among all scenarios, the prevalence of ID (the proportion of individuals who ever have an ID) in the control arm ranges from 0.210 to 0.601 with the base case having 0.387. The prevalence of ID in the experimental arm ranges from 0 to 0.403 with the base case having no ID. Correlation between $L_{1}$ and $L_{2}$ ranges from 0.130 to 0.761 with the base case having 0.447. The prevalence of the outcome in the control arm ranges from 0.163 to 0.227 with the base case having 0.188 and in the experimental arm ranges from 0.089 to 0.146 with the base case having 0.114. For each scenario, the ‘true’ value of the estimand is provided.

Among all the scenarios included in this study, we checked the missing values and outliers in the estimates data sets. We compared the EmpSE with ModSE for all the methods under investigation.

**Figure 2.**
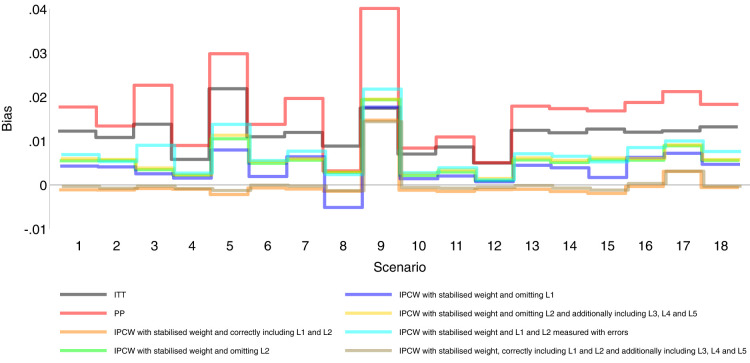
Bias comparison among methods in Scenarios 1–18. Monte Carlo standard error (MCSE) ≤ 0.002.

**Figure 3.**
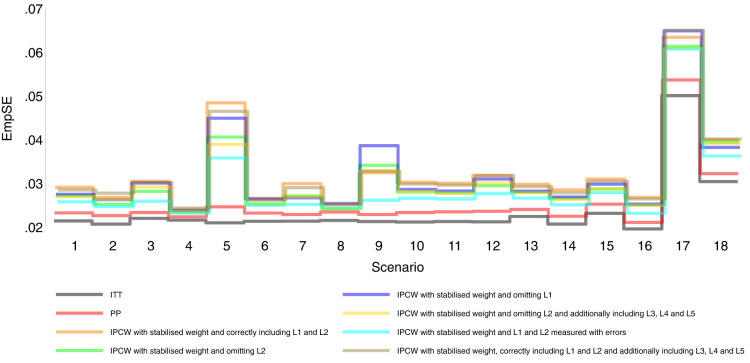
Empirical standard error comparison among methods in Scenarios 1–18.

**Figure 4.**
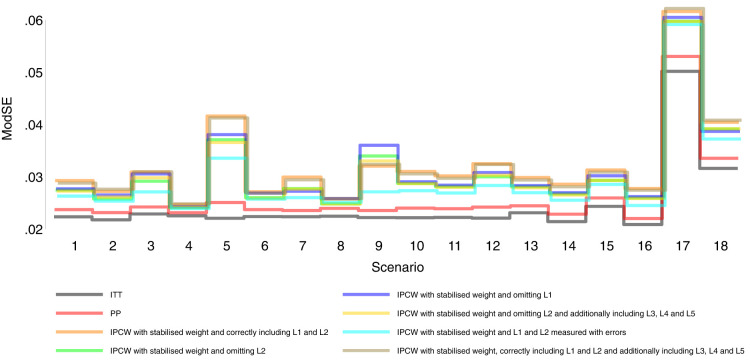
Model-based standard error comparison among methods in Scenarios 1–18.

**Figure 5.**
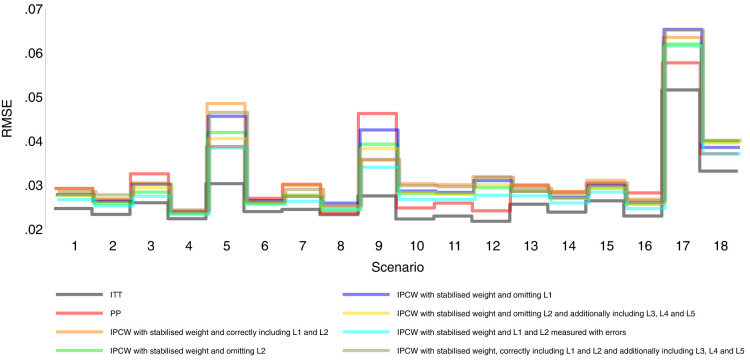
Root mean squared error comparison among methods in Scenarios 1–18.

**Figure 6.**
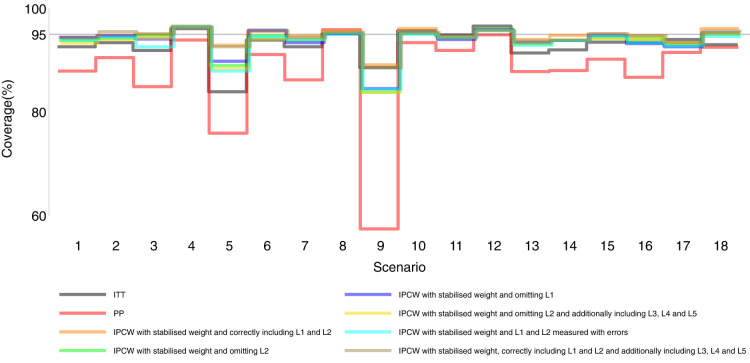
Coverage comparison among methods in Scenarios 1–18.

Our main focus in this study is the accuracy of methods. In theory, with the outcome model correctly specified, the differences in the denominator which are different forms of variable combinations influence the accuracy of the estimates and the numerator in stabilised weight influences the SEs. As expected, IPCW with unstabilised and stabilised weight yield similar point estimates of the risk difference (Supplemental Material C.4). In Table , where we listed a series of IPCW implementations, within each category of suboptimal implementation of IPCW, the methods show similar accuracy. To be concise, in this section, we only present the results comparing a selection of methods: ITT, PP, IPCW with NUC, IPCW omitting a confounder, IPCW with mis-specified TVCs, IPCW with measurement errors on confounders and IPCW with unnecessary TVCs. See Supplemental Material C.4 for full results of all methods. To address each question raised in Section 3.1, we first assess each method separately with a main focus on the bias across all scenarios and then compare the performance of all methods with all performance measures considered. In this section, the comparisons between methods are visualised in nested loop plots for all performance measures investigated (see Figures 2 to 6). We also provide further details on results for these plots in Tables C3 to C8 in Supplemental Material C.

### 4.1. Simple methods

Figures 2 to 6 show detailed results of the performance of simple methods including ITT (grey lines) and PP (red lines) across all scenarios. ITT analysis underestimated the estimand of interest in all scenarios. This is not surprising since the effect of subsequent treatment which by our design has a larger benefit than the treatment assigned is also taken into account in ITT analysis. Different effects of subsequent treatment in one or both groups are also considered (see Scenarios 19–22 in Supplemental Material C.1), and we can see different directions and magnitude of bias by ITT (see Scenarios 19–22 in Supplemental Material C.4). For PP analysis, we see a positive bias in all scenarios. The prevalence of ID in the control arm in single-arm-ID cases (or the difference in the prevalence of ID between arms in both-arm-ID cases) has an important impact on the bias (see Scenarios 1, 4 and 5 for single-arm-ID cases and Scenarios 10–12 for both-arm-ID cases). As shown by Scenarios 1, 6 and 7 the effect of confounder(s) on ID also influences the bias, and in particular in Scenario 9 where confounder(s) are deterministic, PP yields a large bias. Specifically in Scenario 8, we see a very small bias of 0.003 which can be explained by the ‘cancelling’ of confounding by L1 and L2 in different directions.

### 4.2. IPCW with confounders correctly specified

Orange lines in Figures 2 to 6 show detailed results of the performance of IPCWsL1L2. When the weighting model in IPCW is correctly specified without residual confounding and unnecessary TVCs, IPCWsL1L2 yields unbiased estimates in almost all scenarios except in Scenario 9 with deterministic ID and Scenario 17 with a sample size of 200. We see a large positive bias of 0.015 in the former scenario with structural non-positivity. There is a small positive bias in the latter case with stochastic non-positivity when the sample size is small. This is thought to be caused by empty cells (combinations of covariates) occurring which influences the fitting of the weighting model, which in turn leads to poorly calculated weights and bias. In addition, smaller SEs are obtained when the prevalence of ID decreases (Scenario 4) and the effect of confounder on ID decreases (Scenario 6). Different from the strict violation of non-positivity that leads to biased estimates shown in Scenario 9 and 17, as the prevalence of ID increases, participants with certain properties are almost certain to have ID (i.e. near non-positivity) leading to an increase in SE.

### 4.3. IPCW with residual confounding

IPCW with residual confounding here includes IPCW with missing confounders and IPCW with mis-specified confounders. We now look at green lines for IPCWsL1, blue lines for IPCWsL2 and yellow lines for IPCWsL1L3L4L5.

IPCWsL1 missing confounder L2 and IPCWsL2 missing confounder L1 presented biased estimates in all scenarios. IPCWsL2 provide a slightly smaller bias, larger RMSE and larger coverage than IPCWsL1 because L2 was designed to be a stronger confounder. But the difference is very small and it is likely to be caused by the difference of effect in the outcome model being small. A negative bias is observed for IPCWsL2 in Scenario 8 because L2 here was set to have effects on ID and the outcome in different directions. The different directions of bias yielded by IPCWsL1 and IPCWsL2 in Scenario 8 are caused by the different directions of confounding caused by L1 and L2. Except for that, a positive bias is seen for both implementations in all scenarios. Mis-specified IPCWsL1L3L4L5 with a missing confounder L2 and three unnecessary confounders L3, L4 and L5 presented biased estimates in all scenarios. IPCW with residual confounding caused by missing or mis-specified confounder(s) share the same factors that affect the bias as mentioned in IPCW with NUC.

### 4.4. IPCW with unnecessary TVCs

Brown lines show detailed results of the performance of IPCWsL1L2L3L4L5. When too many covariates are selected with no missing confounders but unnecessary TVCs (L3, L4 and L5) included, IPCWsL1L2L3L4L5 provides unbiased estimates in most scenarios except for the scenarios where the positivity assumption is violated (Scenarios 9 and 17). Compared with correctly specified IPCWsL1L2 (results in orange), the expected impact on SEs by including unnecessary TVCs is not substantial.

### 4.5. IPCW with TVCs measured with error

Results in cyan show the performance of IPCWsL1 ${}^{*}$ L2 ${}^{*}$ . Estimates with positive bias are obtained by IPCW with measurement error (IPCWsL1 ${}^{*}$ L2 ${}^{*}$ ) in all scenarios. Similar conclusions are drawn for factors influencing the bias of the estimates as in other IPCW implementations mentioned above.

### 4.6. Comparing PP and IPCW

We first look at the bias which is our first-order performance measure (see Figure 2) of interest. In almost all scenarios, bias yielded by suboptimal IPCW implementations (coloured green, blue, yellow, cyan and brown) lies in between the bias by IPCW with correct specification (IPCWsL1L2 coloured orange) and PP (coloured red) and all IPCW methods present less biased estimates than PP. However, different results are observed in Scenario 8. When the effect of confounder $L_{1}$ on ID and the effect of confounder $L_{1}$ on ID are in the opposite direction, the performances of the methods are different from those in other scenarios (see Figure B1(b) in the Supplemental Material). The bias yielded by IPCWsL1, IPCWsL1L3L4L5 and bias by PP are both 0.003 and the bias by IPCWsL2 is − 0.005 whose absolute value is larger than that by PP (see Scenario 8 in Table C16 in the Supplemental Material). We observe that PP yields a lower coverage percentage than IPCW in most scenarios except for Scenario 8 because the undercoverage of the methods is mainly caused by bias. Regarding the SEs, IPCW yields moderately larger SEs than PP when the prevalence of ID is high (Scenario 5) or the positivity assumption is violated (Scenarios 9 and 17) and IPCW yields slightly larger SEs in other scenarios. For RMSE, PP slightly outperforms IPCW when two confounders (L1 and L2) are highly correlated (Scenario 3) or there is deterministic ID (Scenario 9) while IPCW performs no worse than PP in all the other scenarios.

## 5. Case study

ODYSSEY was an NI trial investigating whether dolutegravir-based (DTG) antiretroviral therapy (ART) was noninferior to standard of care (SOC) with non-dolutegravir-based ART in children and adolescents living with HIV type 1 (HIV-1). The published analysis followed the ITT principle for the primary analysis and implemented simple PP with exclusion and censoring as a supplementary analysis.^46^ In this work, we focus on handling ICEs. Missing data caused by loss to follow-up can be addressed using appropriate methods (including IPCW) and we will explain more in Section 6. Focusing on estimating a hypothetical estimand, we started with planning the analysis followed by conducting the analysis in the trial data based on the analysis plan. We also repeated the published analysis with details provided in Supplemental Material D.

### 5.1. Plan for analysis

The trial has already been run and analysed. However, to illustrate how an analysis plan could be written, we first imagine that we are writing the trial SAP and have not seen the outcome data. The primary analysis targeted a treatment policy estimand which compared randomly assigned treatments in the presence of any ICEs denoted by the following equation:

(11) $$RD_{TP}=P(T\leq 96|Z=1)-P(T\leq 96|Z=0)$$

where $RD_{TP}$ denotes the treatment policy estimand. Z denotes the randomised group ( Z=1 for the DTG group and Z=0 for the SOC group) and T denotes time to treatment failure.

Here we focused on estimating a hypothetical estimand in the absence of ICEs. First, we defined two ICEs with ICE 1 being failure to meet inclusion criteria and ICE 2 being deviation from the treatment strategy under investigation. Then we specified a hypothetical estimand as the treatment effect in the absence of ICE 1 and ICE 2 denoted by the following equation:

(12) $$RD_{HYP}=P(T_{Z=1}^{I_{1}=0,{\bar{I}}_{2}=0}\leq 96)-P(T_{Z=0}^{I_{1}=0,{\bar{I}}_{2}=0}\leq 96)$$

where $RD_{HYP}$ denotes the hypothetical estimand. $I_{1}$ and $I_{2}$ denote ICE 1 and ICE 2, ${\bar{I}}_{2}$ denotes the history of ICE 2 occurrence.

The outcome modelling and estimation process were planned to follow the main analysis^46^ (see Supplemental Material D). To handle the ICEs, observations were censored at the occurrence of an ICE. To account for the potential selection bias caused by censoring, we identified baseline covariates (age, sex and WHO performance stage) and time-varying covariates (CD4 count, CD4/CD8 ratio and viral load) to estimate the weights (see equations (3) and (5)). Based on our current knowledge, we have identified all important confounders. Though the plausibility of NUC cannot be assessed statistically, we can explore by adding more potential confounders into the weighting model. We planned the sensitivity analysis using IPCW with baseline weight or body mass index (BMI) added to the set of selected confounders mentioned above. If there is unmeasured confounding, it can be in a direction that is either the same or the opposite to the measured confounding. Since we thought the latter case was not very likely to occur, IPCW could be seen as a safer option than PP for ODYSSEY data.

### 5.2. Conduct analysis

Now we use the data from the completed ODYSSEY trial to describe how to conduct analysis on a hypothetical estimand given the collected trial data. First, we described the properties of the actual receipt of treatments and ICEs (see Table 5). Overall, the prevalence of ICEs was quite low. A total of 30 (4%) participants had ICE 1 (23 randomised within incorrect strata; four starting second-line; three did not meet baseline VL requirements). A total of 33 (5%) participants had ICE 2 (a change of third agent for toxicity, protocol deviation, or pregnancy or ART discontinuation for > 31days). Therefore, small differences were expected between the treatment policy estimand and the hypothetical estimand and also between estimates by PP and IPCW.

**Table 5.** Receipt of treatment for each treatment regimen.

	Post-randomisation no. (%)		
Treatment arm	No changes	ICE 1	ICE 2	Total
DTG	329	10	11	350
	(94.00)	(2.86)	(3.14)	(100.00)
SOC	315	20	22	357
	(88.24)	(5.60)	(6.16)	(100.00)
Total	644	30	33	707
	(91.09)	(4.24)	(4.67)	(100.00)

ICE: intercurrent event; DTG: dolutegravir; SOC: standard of care.

Following the plans described in the previous section, the IPCW estimator was implemented to estimate the hypothetical estimand. ITT, PP and IPCW not weighting for ICE 1 were also included as references. Table 6 shows a summary of the results. Targeting a hypothetical estimand, all estimators provided quite similar estimates though IPCW methods provided slightly smaller treatment effect. We also conducted the planned sensitivity analysis exploring more potential confounders (weight and BMI) and Table D1 in Supplemental Material D showed a summary of results. No obvious impact was observed on the estimated treatment effect when we additionally included baseline weight or BMI in the weighting model, so we thought all important confounders (measured) were included. Given that no unmeasured confounders potentially leading to confounding in a direction different from that of the measured confounding are identified based on our current knowledge, IPCW2u and IPCW2s (in bold) were considered to be safe alternatives to PP.

**Table 6.** Analysis results by different estimators for defined estimands.

		Estimate ${}^{a}$		
Estimand	Estimator	EST	SE	95% CI
Treatment policy	ITT ${}^{b}$	− 0.080	0.028	( − 0.131, − 0.026)
Hypothetical	PP ${}^{b}$	− 0.068	0.029	( − 0.123, − 0.011)
	IPCW1u	− 0.061	0.033	( − 0.123, 0.004)
	IPCW1s	− 0.061	0.031	( − 0.114, 0.009)
	**IPCW2u** ${}^{c}$	** − 0.062**	**0.032**	(** − 0.117**, **0.008**)
	**IPCW2s** ${}^{c}$	** − 0.062**	**0.031**	(** − 0.116**, **0.004**)

*Note:* EST: point estimate; SE: standard error; 95% CI: 95% confidence interval; IPCW1u: IPCW with unstabilised weight for ICE 2; IPCW1s: IPCW with stabilised weight for ICE 2; IPCW2u: IPCW with unstabilised weight for ICE 1 and ICE 2; IPCW2s: IPCW with stabilised weight for ICE 1 and ICE 2.

${}^{a}$ Pre-specified NI margin: 0.100.

${}^{b}$ CIs are slightly different from those in the published paper. This is thought to be caused by different seeds used in bootstrapping.

${}^{c}$ Estimators specified for the hypothetical estimand.

## 6. Discussion

PP is widely adopted in trials but is prone to bias because it assumes non-informative censoring, which is violated when covariates causing ID are associated with the counterfactual outcomes. IPCW as an elaborated version of PP attempts to remove the selection bias by giving extra weight to the participants who remain uncensored with the information on identified confounders. Though it is commonly perceived that both PP and IPCW can provide biased estimates of a hypothetical estimand if their assumptions are violated, it is less well-known whether IPCW is a safer choice to obtain less biased estimates compared with PP.

We investigated the performance of PP compared with different implementations of IPCW which are likely to occur in real-world practice in a series of scenarios. Factors in the TVC model, ID model and outcome model are varied to cover a broader range of realistic trial settings. We included different types of TVCs including confounders, non-confounding factors that only influence ID and non-confounding factors that only influence the outcome. For the ID mechanism, we covered both the deterministic ID case and stochastic case depending on confounders considering decisions of IDs occurring in practice can either be physician-driven or patient-driven. Physician-driven IDs have less vague criteria (e.g. a specific threshold of biomarker values or tumour size) and those disease-related data are often collected so the NUC assumption is less likely to be violated but positivity is more likely to be violated. Patient-driven IDs are more likely to have residual confounding with omitted confounders while the positivity assumption is more likely to be satisfied. Outcome event rate and sample size are also varied so that our conclusions are more generalisable to different trial settings. We included many potential implementations of IPCW with different choices of TVCs and with varied data quality. IPCW with TVCs collected with insufficient frequency and measurement errors in ID data were also considered. We see the implementation of IPCW with only baseline measurement of TVCs as an extreme case of inadequate frequency and the case with measurement error is similar to the case with measurement errors in ID.

Under the data-generating mechanism explored in this study, IPCW implemented with all forms of variable selection outperforms PP for bias except for a less common case where the effects of two confounders are in opposite directions and cancel. That is to say, in reality, if the selection bias by different confounders is in opposite directions, then the total selection bias may happen to be smaller than the residual selection bias when omitting a confounder, so PP may outperform IPCW omitting a confounder. We conducted the simulation in the setting where PP censoring is informative. However, the IPCW estimator was shown to provide reliable point estimates and is guaranteed to be more efficient than the ordinary estimator without adjustment when the censoring is independent,^42^ and was also shown to perform well in the absence of selection bias where PP offers unbiased estimates in previous simulation studies.^55^ When the PP censoring is non-informative, implementing IPCW is actually adjusting for unnecessary covariates and this is similar to the cases in our simulation where we adjust for unnecessary covariates in IPCW. Therefore, we expect the results to apply in most trial settings with different ID mechanisms and conclude that IPCW is a safe alternative to PP in terms of accuracy.

In a case study, we illustrated how IPCW could be proposed when planning for the analysis and demonstrated that IPCW was a safe alternative to PP. ODYSSEY showed a special but ideal case of trial conduct with most patients following the correct treatment strategy with fairly low prevalence of ICEs. There may be concerns about the extra cost of collecting extensive data to account for potential confounding while the gain in adopting IPCW under such trial settings is not obvious. However, it is hard to predict the properties of potential ICEs at the trial planning stage and the desirable case with low prevalence may not always be the case. When the prevalence of ICEs ends up being moderate or high in the trial, PP is likely to be subject to substantial bias when estimating the hypothetical estimand. Such an estimand could be estimated more accurately by a pre-specified IPCW estimator with measured confounders, but would not be estimable were there no sufficient data on confounders available.

We believe that IPCW should be used in trials targeting a hypothetical estimand as a substitute for PP whenever TVCs are planned to be collected or can be collected. Though we are aware in theory and have seen from the results that IPCW usually provides larger SEs than PP, but the loss in precision is small in most scenarios while the accuracy gained by IPCW is obvious. However, we should also bear in mind the special case where PP can perform better than IPCW with missing confounder(s). The use of IPCW will need careful consideration regarding the status of the trial. For trials already conducted, if there are potential unmeasured confounders leading to the confounding that we anticipate to be in a different direction from the measured confounding, IPCW should be considered as an analysis method but with caution. We do not consider this as a case that is likely to occur in practice but this is seen as the only situation that can be harmful by replacing PP with IPCW for analysis. For trials not yet conducted, when we specify hypothetical estimands in the SAP, IPCW should be identified as the analysis method rather than PP. To satisfy the assumption required by IPCW, data including potential confounders should be specified based on substantive knowledge in a structural way using DAG. We also need sufficient information on the ID (e.g. reasons, time of occurrence and subsequent treatment) as this may affect the specification of the ID weighting model. In such cases, we collect all the confounders based on our knowledge and we do not know if there are any confounders which are in a different direction since they are unknown rather than unmeasured.

In practice, some trialists use multiple methods and use the similarity of the estimates by different methods to assess the reliability of the results. However, we should not simply rely on the comparisons between estimates by different estimators since we can not know the true value or the actual plausibility of the assumptions required by each method in a trial in practice. When the assumptions of both methods do not hold, we can still observe similar estimates by them which can be biased. This is shown both in the comparison of PP and IPCW (see Scenario 8) and in the comparison of ITT and PP (see Scenarios 19–22 in Supplemental Material C.4). Therefore, we should always focus on evaluating the model assumptions and we should be more confident about the method(s) specified in the SAP when the key assumptions are thought to be plausible with well-collected data. On the other hand, given that implementation mistakes are likely to occur due to the computational complexity of IPCW, the comparison between PP and IPCW may be helpful in recognising potential mis-implementation of IPCW. When there are substantial differences between the results either in point estimates or SEs, we should be able to give explanations. When there are important differences in SEs, it is worth checking the implementation details (e.g. cluster for individuals in a time-updated dataset) since IPCW does not follow an ordinary SE calculation process. In most cases, we should be aware that when the NUC assumption is considered to be satisfied, PP is subject to selection bias and IPCW corrects for such bias.

There are a few remaining limitations to this study, some of which point to the need for further work. First, the validity of these methods heavily relies on the plausibility of their assumptions which are untestable. The performance of the methods changes in terms of the real structure of TVCs, ID and outcome models. We only included a limited set of scenarios with a limited range of values of the factors and the performance in more extreme cases such as scenarios with very strong confounding leading to the failure of IPCW are not covered. However, our settings investigated here are realistic for the use of IPCW. Even in those uncovered cases, for example, when we have a very strong confounder, while IPCW is subject to a larger bias, PP is also subject to a larger bias in most cases since PP omits confounders. Second, we assumed the correct specification of both the ID and outcome model but in practice both the ID and outcome models are likely to be mis-specified with missing terms. We expect bias caused by a mis-specified model (e.g. missing interaction term or polynomial terms) to be smaller than that caused by NUC. Third, the simulation is in a setting where both time to occurrence of ID and time to endpoint are discrete which may not be representative of some real-world settings. However, the conclusions drawn from this study can be extended to those cases and we would expect similar conclusions to be drawn. Last, we assumed no missing data in the outcome which is another issue that commonly occurs in RCTs. To handle the missing data problem, we point readers towards some publications on a variety of methods that may be adopted in response to different missing mechanisms.^56,57^ ID and missing data problems can either be dealt with separately or together. IPCW can also be used in handling censoring by loss to follow-up. We are aware that missing data can potentially worsen the violation of the assumptions required by IPCW leading to the failure of IPCW, and a comparison of PP and IPCW can be explored in such cases in further studies.

While PP is often adopted and IPCW is used infrequently, we hope that the comparisons of estimated treatment effects by PP analysis versus IPCW in the simulation can shed light on the performance of these methods in real applications in practice. We are aware that there may be concerns about the use of IPCW. On the one hand, when the selection bias by different confounders is in opposite directions and cancels out, PP can be less biased. However, unless there are unmeasured confounders identified that are in the opposite direction to the measured confounding in completed trials, IPCW is not likely to perform worse than PP. On the other hand, even though IPCW outperforms PP in most circumstances except for the less common case where the selection bias by different confounders cancels, both PP and IPCW can give substantial bias when key assumptions are violated. For trials with a small sample size, we may obtain biased estimates and large SEs because the positivity assumption is likely to be violated. For completed trials with unmeasured confounders, poorly measured confounders (i.e. with missing values or measurement errors) or model misspecification, bias by IPCW may be non-ignorable. When designing trials, we suggest trial statisticians should be cautious about the specification of, and the data collection plan on ID and confounders in SAPs. Though there may still be residual confounding which is unknown rather than unmeasured, estimates by IPCW analyses can be seen as reliable based on current knowledge. In most situations, IPCW yields less bias than PP and with careful investigation of the plausibility of assumptions, IPCW provides reliable estimates. In conclusion, it is safe to substitute simple PP with IPCW as its extended version for the estimate of a hypothetical estimand.

## Supplemental Material

sj-pdf-1-smm-10.1177_09622802241289559 - Supplemental material for Is inverse probability of censoring weighting a safer choice than per-protocol analysis in clinical trials? Supplemental material, sj-pdf-1-smm-10.1177_09622802241289559 for Is inverse probability of censoring weighting a safer choice than per-protocol analysis in clinical trials? by Jingyi Xuan, Shahrul Mt-Isa, Nicholas Latimer, Helen Bell Gorrod, William Malbecq, Kristel Vandormael, Victoria Yorke-Edwards and Ian R White in Statistical Methods in Medical Research
