# Supplementary material for: Is inverse probability of censoring weighting a safer choice than per-protocol analysis in clinical trials?
Source: Stat Methods Med Res. 2024 Dec 12;34(2):286–306. doi: 10.1177/09622802241289559 (PMC11874582; doi:10.1177/09622802241289559)
Supplement: sj-pdf-1-smm-10.1177_09622802241289559 - Supplemental material for Is inverse probability of censoring weighting a safer choice than per-protocol analysis in clinical trials? [file sj-pdf-1-smm-10.1177_09622802241289559.pdf]

Is inverse probability of censoring weighting a safer choice than per-protocol analysis in clinical trials?

SUPPLEMENTARY MATERIALS

Appendix A: Estimand framework explained with an example
Appendix B: Data-generating details for simulation on IPCW safety
Appendix C: Results for simulation on IPCW safety
Appendix D: Supplementary information for the case study

Appendix A Estimand framework explained with an example

In this section, we provide a detailed description of the estimand framework and how to construct an estimand. An example in an HIV trial setting which refers to ODYSSEY is provided here. We considered four trial objectives that may be of interest and constructed the estimands targeting each of the objectives (Table A).

Table A1. Estimand attributes and an example in an HIV trial setting

Estimand attribute	Description	Example in an HIV trial setting				
		Trial objectives				
			The treatment effect of assigning the intervention strategy	**The treatment effect if both ICEs had not occurred**	The treatment effect if everyone initiates the correct treatment strategy	The treatment effect if nobody who initiated deviates from the treatment strategy under investigation
Treatment:	The treatment strategy of interest.		SOC with/without incorrect treatment initiation or deviation from treatment strategy vs. DTG with/without incorrect treatment initiation or deviation from treatment strategy	**SOC vs. DTG**	SOC with/without deviation from treatment strategy under investigation vs. DTG with/without deviation from treatment strategy under investigation	SOC with/without incorrect treatment initiation vs. DTG with/without incorrect treatment initiation
Population:	The population of individuals indicated by the trial objective.	HIV-1 infected children younger than 18 years and weighting $\geq 14kg$ (with specified inclusion criteria)				
ICEs with strategies:	Intercurrent events that influence the occurrence or the interpretation of the trial objective.	ICE1: Failure to initiate the correct treatment strategy[b]	Treatment policy strategy	**Hypothetical strategy**	Hypothetical strategy	Treatment policy strategy
		ICE2: Deviation from the treatment strategy under investigation	Treatment policy strategy	**Hypothetical strategy**	Treatment policy strategy	Hypothetical strategy
Outcome variable:	The variable that is used to realise the trial objective.	Virological or clinical failure by 96 weeks				
Population level summary:	A summary of the outcome variable for the comparison between treatment strategies.	Difference in cumulative incidence between two treatment strategies at week 96				

Note: SOC = Standard of care; DTG = Dolutegravir-based ART; ICE = Intercurrent event. The ICEs addressed with hypothetical strategy in bold are intervention deviation.

[a] The hypothetical estimand in bold is explored in the case study in Section 5.

[b] In most cases, this denotes not starting pre-specified backbone.

Appendix B Data-generating details for simulation on IPCW safety

B.1 Data-generating algorithm

For each individual $i = 1 , \dots , n$:

1. At $v = 0$:
   - Generate values for all the covariates (time-independent and time-dependent) at $v = 0$: $L_{i01}, L_{i02}, L_{i03}, L_{i04}, L_{i05}$.
   - Generate treatment assignment by randomisation: Z_i
   - Set indicator of intervention deviation: $C_{0i} = 0$
   - Set time on and off treatment assigned by randomisation: $T_{i0on} = 0$; $T_{i0off} = 0$
   - Set indicator of outcome: $Y_{i0} = 0$
2. At $v = 1, 2, ..., 8$ (if $Y_{i(v-1)} = 0$):
   - Generate time-varying covariates: $L_{iv1}, L_{iv2}, L_{iv3}, L_{iv4}, L_{iv5}$
   - Update indicator of intervention deviation C_{iv}:
     - If $C_{i(v-1)} = 1$:
       * Set C_{iv} to NULL
     - If $C_{i(v-1)} = 0$:
       * Generate indicator of intervention deviation C_{iv} at visit v
   - Generate indicator of outcome: Y_{iv}
   - Update periods on and off treatment assigned: T_{ivon} and T_{ivoff}

B.2 Numerical values for simulation models

$$\boldsymbol{\mu_0} = (11.95, 1.97, 5.79, -1.10, 4.39, 95.77, 138.13) \tag{1}$$

$$\boldsymbol{m} = (2.91, 1.00, 0.69, -5.07, 1.30, 1.26, 31.71) \tag{2}$$

$$\boldsymbol{n} = (17.99, 4.00, 8.00, 2.67, 6.64, 374.18, 256.00) \tag{3}$$

$$\boldsymbol{\Sigma_l} = \begin{pmatrix} 1 & 0.04 & -0.27 & -0.20 & -0.05 & 0.01 & 0.10 \\ 0.04 & 1 & -0.30 & -0.30 & 0.17 & 0.03 & -0.03 \\ -0.27 & -0.30 & 1 & 0.09 & -0.42 & -0.13 & 0.07 \\ -0.20 & -0.30 & 0.09 & 1 & -0.46 & -0.14 & 0.09 \\ -0.05 & 0.17 & -0.42 & -0.46 & 1 & 0.14 & -0.14 \\ 0.01 & 0.03 & -0.13 & -0.14 & 0.14 & 1 & 0.10 \\ 0.10 & -0.03 & 0.07 & 0.09 & -0.14 & 0.10 & 1 \end{pmatrix} \tag{4}$$

$$\boldsymbol{\Sigma_m} = \begin{pmatrix} 1 & 0.04 & -0.27 & -0.20 & -0.05 & 0.01 & 0.10 \\ 0.04 & 1 & -0.30 & -0.30 & 0.17 & 0.03 & -0.03 \\ -0.27 & -0.30 & 1 & 0.49 & -0.42 & -0.13 & 0.07 \\ -0.20 & -0.30 & 0.49 & 1 & -0.46 & -0.14 & 0.09 \\ -0.05 & 0.17 & -0.42 & -0.46 & 1 & 0.14 & -0.14 \\ 0.01 & 0.03 & -0.13 & -0.14 & 0.14 & 1 & 0.10 \\ 0.10 & -0.03 & 0.07 & 0.09 & -0.14 & 0.10 & 1 \end{pmatrix} \tag{5}$$

$$\boldsymbol{\Sigma_h} = \begin{pmatrix} 1 & 0.04 & -0.27 & -0.20 & -0.05 & 0.01 & 0.10 \\ 0.04 & 1 & -0.30 & -0.30 & 0.17 & 0.03 & -0.03 \\ -0.27 & -0.30 & 1 & 0.89 & -0.42 & -0.13 & 0.07 \\ -0.20 & -0.30 & 0.89 & 1 & -0.46 & -0.14 & 0.09 \\ -0.05 & 0.17 & -0.42 & -0.46 & 1 & 0.14 & -0.14 \\ 0.01 & 0.03 & -0.13 & -0.14 & 0.14 & 1 & 0.10 \\ 0.10 & -0.03 & 0.07 & 0.09 & -0.14 & 0.10 & 1 \end{pmatrix} \tag{6}$$

$$\boldsymbol{\rho} = (\rho_0, \rho_{31}, \rho_{32}, \rho_{34}) = (-3.00, 0.46, -0.83, -0.01) \tag{7}$$

$$\boldsymbol{\Lambda_l} = \begin{pmatrix} 1 & 0.01 \\ 0.01 & 1 \end{pmatrix} \tag{8}$$

$$\boldsymbol{\Lambda_m} = \begin{pmatrix} 1 & 0.40 \\ 0.40 & 1 \end{pmatrix} \tag{9}$$

$$\boldsymbol{\Lambda_h} = \begin{pmatrix} 1 & 0.70 \\ 0.70 & 1 \end{pmatrix} \tag{10}$$

Table B1. Summary of numerical values for parameters in TVC model

TVC	β_{q0}	β_{q1}	β_{q2}	β_{q3}	$\boldsymbol{\beta_{q4}}$	β_{q5}	sd_q
L_1	2.39	0.20	*	*	(-0.02,-0.04, -0.01)	0.66	0.70
L_2	0.11	0.10	*	*	(-0.01,-0.07, -0.02)	0.72	0.78
L_3	1.29	-0.17	0.24	0	(0,0,0.05)	0.25	0.81
L_4	67.47	0	7.89	0	(0,-2.99,0)	0	0.57
L_5	84.00	-13.97	0	0	(-0.92,0,-3.78)	0.42	53.55

* Values for β_{12}, β_{13}, β_{22} and β_{23} vary and details are shown in Table B2

$$\boldsymbol{\theta_3} = (0.24, 0.50, -0.83) \tag{11}$$

$$\mu_2 = -0.13 \tag{12}$$

$$\boldsymbol{\mu_6} = (0.04, 0.18, 0) \tag{13}$$

Table B2. Summary of varying parameter values in Scenario 1-22

No.	θ_0	θ_1	θ_2	$\boldsymbol{\theta_4}$	θ_5	μ_0	μ_1	μ_3	μ_4	μ_5	$\boldsymbol{\mu_7}$	$\boldsymbol{\Sigma}$	$\boldsymbol{\Lambda}$	n_{obs}	β_{12}	β_{13}	β_{22}	β_{23}
**1**	**-4.04**	**0.1**	**0**	**(-0.48,-0.46,0,0.01,0.01)**	**0**	**-3.01**	**-0.1**	**-0.04**	**-0.19**	**-0.03**	**(-0.50,-0.50,0.78,0,0)**	$\boldsymbol{\Sigma_m}$	$\boldsymbol{\Lambda_m}$	**1000**	**0.21**	**-**	**0.17**	**-**
2	-4.04	0.1	0	(-0.48,-0.46,0,0.01,0.01)	0	-3.01	-0.1	-0.04	-0.19	-0.03	(-0.50,-0.50,0.78,0,0)	$\boldsymbol{\Sigma_l}$	$\boldsymbol{\Lambda_l}$	1000	0.21	-	0.17	-
3	-4.04	0.1	0	(-0.48,-0.46,0,0.01,0.01)	0	-3.01	-0.1	-0.04	-0.19	-0.03	(-0.50,-0.50,0.78,0,0)	$\boldsymbol{\Sigma_h}$	$\boldsymbol{\Lambda_h}$	1000	0.21	-	0.17	-
4	-5.04	0.1	0	(-0.48,-0.46,0,0.01,0.01)	0	-3.01	-0.1	-0.04	-0.19	-0.03	(-0.50,-0.50,0.78,0,0)	$\boldsymbol{\Sigma_l}$	$\boldsymbol{\Lambda_l}$	1000	0.21	-	0.17	-
5	-3.04	0.1	0	(-0.48,-0.46,0,0.01,0.01)	0	-3.01	-0.1	-0.04	-0.19	-0.03	(-0.50,-0.50,0.78,0,0)	$\boldsymbol{\Sigma_l}$	$\boldsymbol{\Lambda_l}$	1000	0.21	-	0.17	-
6	-5.30	0.1	0	(-0.28,-0.46,0,0.01,0.01)	0	-3.01	-0.1	-0.04	-0.19	-0.03	(-0.50,-0.50,0.78,0,0)	$\boldsymbol{\Sigma_l}$	$\boldsymbol{\Lambda_l}$	1000	0.21	-	0.17	-
7	-3.00	0.1	0	(-0.68,-0.46,0,0.01,0.01)	0	-3.01	-0.1	-0.04	-0.19	-0.03	(-0.50,-0.50,0.78,0,0)	$\boldsymbol{\Sigma_l}$	$\boldsymbol{\Lambda_l}$	1000	0.21	-	0.17	-
8	-10.00	0.1	0	(0.48,-0.46,0,0.01,0.01)	0	-3.01	-0.1	-0.04	-0.19	-0.03	(-0.50,-0.50,0.78,0,0)	$\boldsymbol{\Sigma_l}$	$\boldsymbol{\Lambda_l}$	1000	0.21	-	0.17	-
9	-4.04	0.1	0	(-0.48,-0.46,0,0.01,0.01)	0	-3.01	-0.1	-0.04	-0.19	-0.03	(-0.50,-0.50,0.78,0,0)	$\boldsymbol{\Sigma_l}$	$\boldsymbol{\Lambda_l}$	1000	0.21	-	0.17	-
10	-4.04	0.1	0	(-0.48,-0.46,0,0.01,0.01)	1	-3.01	-0.1	-0.04	-0.19	-0.03	(-0.50,-0.50,0.78,0,0)	$\boldsymbol{\Sigma_l}$	$\boldsymbol{\Lambda_l}$	1000	0.21	0	0.17	0
11	-4.04	0.1	-0.51	(-0.48,-0.46,0,0.01,0.01)	1	-3.01	-0.1	-0.04	-0.19	-0.03	(-0.50,-0.50,0.78,0,0)	$\boldsymbol{\Sigma_l}$	$\boldsymbol{\Lambda_l}$	1000	0.21	0	0.17	0
12	-4.04	0.1	0.51	(-0.48,-0.46,0,0.01,0.01)	1	-3.01	-0.1	-0.04	-0.19	-0.03	(-0.50,-0.50,0.78,0,0)	$\boldsymbol{\Sigma_l}$	$\boldsymbol{\Lambda_l}$	1000	0.21	0	0.17	0
13	-4.04	0.1	0	(-0.48,-0.46,0,0.01,0.01)	0	-3.01	0	0	-0.19	-0.03	(-0.50,-0.50,0.78,0,0)	$\boldsymbol{\Sigma_l}$	$\boldsymbol{\Lambda_l}$	1000	0.21	-	0.17	-
14	-4.04	0.1	0	(-0.48,-0.46,0,0.01,0.01)	0	-3.01	-0.3	-0.07	-0.19	-0.03	(-0.50,-0.50,0.78,0,0)	$\boldsymbol{\Sigma_l}$	$\boldsymbol{\Lambda_l}$	1000	0.21	-	0.17	-
15	-4.04	0.1	0	(-0.48,-0.46,0,0.01,0.01)	0	-3.90	-0.1	-0.04	-0.19	-0.03	(-0.30,-0.50,0.78,0,0)	$\boldsymbol{\Sigma_l}$	$\boldsymbol{\Lambda_l}$	1000	0.21	-	0.17	-
16	-4.04	0.1	0	(-0.48,-0.46,0,0.01,0.01)	0	-2.10	-0.1	-0.04	-0.19	-0.03	(-0.70,-0.50,0.78,0,0)	$\boldsymbol{\Sigma_l}$	$\boldsymbol{\Lambda_l}$	1000	0.21	-	0.17	-
17	-4.04	0.1	0	(-0.48,-0.46,0,0.01,0.01)	0	-3.01	-0.1	-0.04	-0.19	-0.03	(-0.50,-0.50,0.78,0,0)	$\boldsymbol{\Sigma_l}$	$\boldsymbol{\Lambda_l}$	200	0.21	-	0.17	-
18	-4.04	0.1	0	(-0.48,-0.46,0,0.01,0.01)	0	-3.01	-0.1	-0.04	-0.19	-0.03	(-0.50,-0.50,0.78,0,0)	$\boldsymbol{\Sigma_l}$	$\boldsymbol{\Lambda_l}$	500	0.21	-	0.17	-
19	-4.04	0.1	0	(-0.48,-0.46,0,0.01,0.01)	1	-3.01	-0.1	-0.04	-0.11	-0.03	(-0.50,-0.50,0.78,0,0)	$\boldsymbol{\Sigma_l}$	$\boldsymbol{\Lambda_l}$	1000	-0.21	-	-0.17	-
20	-4.04	0.1	0	(-0.48,-0.46,0,0.01,0.01)	1	-3.01	-0.1	-0.04	-0.11	-0.03	(-0.50,-0.50,0.78,0,0)	$\boldsymbol{\Sigma_l}$	$\boldsymbol{\Lambda_l}$	1000	-0.21	-0.21	-0.17	-0.17
21	-4.04	0.1	0	(-0.48,-0.46,0,0.01,0.01)	1	-3.01	-0.1	-0.04	-0.11	-0.09	(-0.50,-0.50,0.78,0,0)	$\boldsymbol{\Sigma_l}$	$\boldsymbol{\Lambda_l}$	1000	-0.21	0.21	-0.17	0.17
22	-4.04	0.1	0	(-0.48,-0.46,0,0.01,0.01)	1	-3.01	-0.1	-0.04	-0.19	0.05	(-0.50,-0.50,0.78,0,0)	$\boldsymbol{\Sigma_l}$	$\boldsymbol{\Lambda_l}$	1000	0.21	-0.21	0.17	-0.17

Note: ID in Scenario 9 is set to be certain to occur when L1 $<$ 5 and L2 $<$ -2 representing a case with deterministic non-positivity. The parameter values changed to vary the factor under investigation in each scenario compared with the base-case scenario are coloured red.

B.3 Signed DAG for direction of confounding

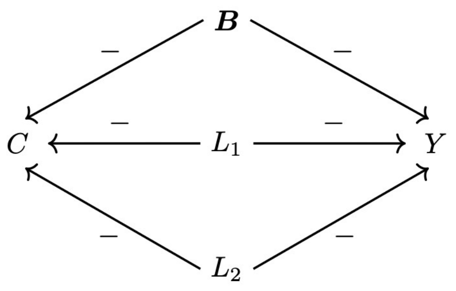

(a) Same direction of confounding by two confounders: confounding by L_1 is negative and confounding by L_2 is also negative.

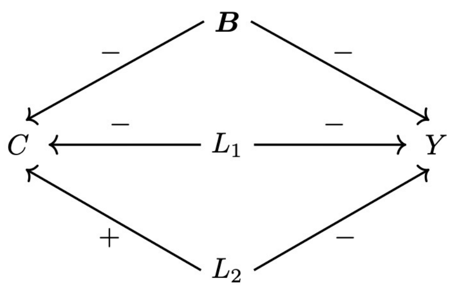

(b) Opposite direction of confounding by two confounders: confounding by L_1 positive while confounding by L_2 negative.

Figure B1. Signed causal DAGs: direction of confounding by two confounders to be the same or the opposite.

Appendix C Results for simulation on IPCW safety

C.1 Summary of simulated scenarios

Table C1. Summary of characteristics of Scenarios 1-22.

Scenario	Prevalence of ID		Corr	True RD
	Control arm	Experimental arm		
1	0.387	0.000	0.447	-0.080
2	0.377	0.000	**0.130**	-0.077
3	0.395	0.000	**0.761**	-0.083
4	**0.210**	0.000	0.447	-0.080
5	**0.601**	0.000	0.448	-0.080
6	0.367	0.000	0.447	-0.080
7	0.366	0.000	0.446	-0.080
8	0.355	0.000	0.449	-0.080
9	0.456	0.000	0.444	-0.080
10	0.387	**0.303**	0.452	-0.080
11	0.387	**0.219**	0.450	-0.080
12	0.387	**0.403**	0.455	-0.080
13	0.387	0.000	0.447	-0.080
14	0.387	0.000	0.447	-0.104
15	0.387	0.000	0.448	-0.087
16	0.387	0.000	0.446	-0.075
17	0.391	0.000	0.447	-0.080
18	0.386	0.000	0.446	-0.080
19	0.387	0.303	0.448	-0.080
20	0.387	0.303	0.462	-0.080
21	0.387	0.303	0.448	-0.080
22	0.387	0.303	0.462	-0.080

Note: ID = Intervention deviation; Corr = Correlation of L_1 and L_2. Scenarios 1–18 are main scenarios covered in the main text exploring PP and IPCW. Scenarios 19–22 are additionally included for situations influencing the performance of ITT.

C.2 Summary of weights of IPCW

Table C2. Ratio (Min/Max) of IPW

	S1	S4	S5	S9	S17	S18
Unstabilised						
IPCWuL2	23.791	7.885	128.565	61.704	10.598	16.249
IPCWuL1	23.811	7.151	95.925	46.290	10.921	16.034
IPCWuL1L3	23.789	7.234	98.339	44.767	10.607	16.031
IPCWuL1L4	24.759	7.520	98.833	49.081	11.560	16.790
IPCWuL1L4L5	25.625	7.580	89.804	49.483	11.270	15.911
IPCWuL1L3L4L5	25.312	7.653	90.522	47.474	11.132	15.932
IPCWuLb1Lb2	22.369	6.251	88.826	26.393	9.873	15.502
IPCWuL1*	15.954	5.409	64.963	13.589	9.151	12.280
IPCWuL1L2	24.050	8.136	88.261	32.054	10.847	15.972
IPCWuL1L2L3	23.941	8.177	88.661	31.688	10.580	16.154
IPCWuL1L2L4	25.962	8.480	93.823	33.178	11.346	16.608
IPCWuL1L2L4L5	26.073	8.550	88.220	33.108	11.078	16.222
IPCWuL1L2L3L4L5	25.785	8.607	87.507	32.684	10.977	16.308
IPCWuL1*L2*	15.588	5.693	66.320	13.037	8.679	11.884
Stabilised						
IPCWsL2	24.690	7.993	134.180	64.844	11.199	16.976
IPCWsL1	24.682	7.254	101.092	48.441	11.406	16.749
IPCWsL1L3	24.648	7.339	103.425	46.840	11.104	16.746
IPCWsL1L4	25.718	7.642	105.938	51.812	12.145	17.619
IPCWsL1L4L5	26.688	7.720	98.159	52.241	11.921	16.676
IPCWsL1L3L4L5	26.356	7.800	98.796	50.097	11.797	16.712
IPCWsLb1Lb2	22.799	6.278	91.612	26.248	10.210	15.857
IPCWsL1*	16.254	5.437	67.092	13.767	9.458	12.594
IPCWsL1L2	25.171	8.289	95.419	33.970	11.529	16.942
IPCWsL1L2L3	25.068	8.333	95.815	33.579	11.283	17.142
IPCWsL1L2L4	27.246	8.660	103.224	35.464	12.131	17.750
IPCWsL1L2L4L5	27.363	8.744	97.703	35.490	11.952	17.293
IPCWsL1L2L3L4L5	27.070	8.805	96.977	35.003	11.860	17.374
IPCWsL1*L2*	16.006	5.746	69.157	13.576	9.056	12.305

C.3 Summary of performance measures by methods

Table C3. Results for simple methods (ITT and PP).

Scenario			Performance measure					
**No.**	**True**	**Description**	**Mean**	**Bias**	**EmpSE**	**RMSE**	**ModSE**	**Coverage(%)**
ITT								
1	-0.080	Base	-0.067	0.012	0.021	0.025	0.022	92.60
2	-0.077	Corr: Low	-0.065	0.011	0.021	0.023	0.022	93.40
3	-0.082	Corr: High	-0.069	0.014	0.022	0.026	0.023	91.90
4	-0.080	ID prevalence: Low	-0.074	0.006	0.022	0.022	0.023	96.10
5	-0.080	ID prevalence: High	-0.057	0.022	0.021	0.030	0.022	83.90
6	-0.080	Confounder-ID Magnitude: Low	-0.068	0.011	0.021	0.024	0.022	93.90
7	-0.080	Confounder-ID Magnitude: High	-0.067	0.012	0.021	0.024	0.022	92.60
8	-0.080	Confounder-ID Sign: $-/+$	-0.070	0.009	0.021	0.023	0.022	95.10
9	-0.080	Deterministic ID	-0.062	0.017	0.021	0.027	0.022	88.60
10	-0.080	Both-arm ID, arm-ID:0	-0.072	0.007	0.021	0.022	0.022	95.70
11	-0.080	Both-arm ID, arm-ID:$-$	-0.071	0.009	0.021	0.023	0.022	94.90
12	-0.080	Both-arm ID, arm-ID:$+$	-0.074	0.005	0.021	0.022	0.022	96.60
13	-0.080	Arm-outcome: Low	-0.044	0.012	0.022	0.026	0.023	91.40
14	-0.104	Arm-outcome: High	-0.093	0.012	0.021	0.024	0.021	92.00
15	-0.087	Confounder-outcome: Low	-0.074	0.013	0.023	0.026	0.024	93.50
16	-0.075	Confounder-outcome: High	-0.062	0.012	0.020	0.023	0.021	93.50
17	-0.080	Sample size: 200	-0.067	0.012	0.050	0.052	0.050	93.99
18	-0.080	Sample size: 500	-0.066	0.013	0.030	0.033	0.032	93.00
PP								
1	-0.080	Base	-0.062	0.018	0.023	0.029	0.024	87.90
2	-0.077	Corr: Low	-0.063	0.013	0.023	0.026	0.023	90.50
3	-0.082	Corr: High	-0.060	0.023	0.023	0.032	0.024	84.90
4	-0.080	ID prevalence: Low	-0.070	0.009	0.022	0.024	0.023	93.90
5	-0.080	ID prevalence: High	-0.049	0.030	0.025	0.039	0.025	75.90
6	-0.080	Confounder-ID Magnitude: Low	-0.066	0.014	0.023	0.027	0.024	91.10
7	-0.080	Confounder-ID Magnitude: High	-0.060	0.020	0.023	0.030	0.024	86.20
8	-0.080	Confounder-ID Sign: $-/+$	-0.076	0.003	0.023	0.024	0.024	95.90
9	-0.080	Deterministic ID	-0.039	0.040	0.023	0.046	0.024	57.40
10	-0.080	Both-arm ID, arm-ID:0	-0.071	0.008	0.023	0.025	0.024	93.40
11	-0.080	Both-arm ID, arm-ID:$-$	-0.068	0.011	0.023	0.026	0.024	91.90
12	-0.080	Both-arm ID, arm-ID:$+$	-0.074	0.005	0.024	0.024	0.024	94.90
13	-0.080	Arm-outcome: Low	-0.038	0.018	0.024	0.030	0.024	87.80
14	-0.104	Arm-outcome: High	-0.087	0.017	0.022	0.028	0.023	88.00
15	-0.087	Confounder-outcome: Low	-0.070	0.017	0.025	0.030	0.026	90.20
16	-0.075	Confounder-outcome: High	-0.056	0.019	0.021	0.028	0.022	86.70
17	-0.080	Sample size: 200	-0.058	0.021	0.054	0.058	0.053	91.50
18	-0.080	Sample size: 500	-0.061	0.018	0.032	0.037	0.034	92.50
Largest MCSE			0.002	0.002	0.001	0.001	0.001	1.56

Note: MCSE = Monte Carlo Standard Error; EmpSE = Empirical Standard Error; RMSE = Root Mean Square Error; ModSE = Model-based Standard Error; Corr: Correlation; ID = Intervention deviation.

Table C4. Results for IPCW with NUC: IPCW with stabilised weight and correctly including L1 and L2 (IPCWsL1L2).

Scenario			Performance measure					
**No.**	**True**	**Description**	**Mean**	**Bias**	**EmpSE**	**RMSE**	**ModSE**	**Coverage(%)**
IPCWsL1L2								
1	-0.080	Base	-0.080	-0.001	0.029	0.029	0.029	94.40
2	-0.077	Corr: Low	-0.077	-0.001	0.027	0.027	0.027	94.80
3	-0.082	Corr: High	-0.083	-0.001	0.030	0.030	0.031	95.00
4	-0.080	ID prevalence: Low	-0.080	-0.001	0.024	0.024	0.025	96.60
5	-0.080	ID prevalence: High	-0.081	-0.002	0.045	0.045	0.042	92.99
6	-0.080	Confounder-ID Magnitude: Low	-0.080	-0.001	0.027	0.027	0.027	95.60
7	-0.080	Confounder-ID Magnitude: High	-0.080	-0.001	0.029	0.029	0.029	94.69
8	-0.080	Confounder-ID Sign: -/+	-0.081	-0.001	0.025	0.025	0.026	95.50
9	-0.080	Deterministic ID	-0.064	**0.015**	0.030	0.034	0.031	**89.09**
10	-0.080	Both-arm ID, arm-ID:0	-0.081	-0.001	0.030	0.030	0.031	96.10
11	-0.080	Both-arm ID, arm-ID:-	-0.081	-0.001	0.030	0.030	0.030	94.50
12	-0.080	Both-arm ID, arm-ID:+	-0.080	-0.001	0.032	0.032	0.032	95.80
13	-0.080	Arm-outcome: Low	-0.057	-0.001	0.030	0.030	0.030	93.99
14	-0.104	Arm-outcome: High	-0.106	-0.002	0.028	0.029	0.029	94.80
15	-0.087	Confounder-outcome: Low	-0.089	-0.002	0.031	0.031	0.031	95.10
16	-0.075	Confounder-outcome: High	-0.075	-0.000	0.027	0.027	0.028	94.70
17	-0.080	Sample size: 200	-0.076	0.003	0.063	0.063	0.062	93.40
18	-0.080	Sample size: 500	-0.080	-0.001	0.040	0.040	0.040	96.10
Largest MCSE			0.002	0.002	0.001	0.002	0.001	0.99

Note: MCSE = Monte Carlo Standard Error; EmpSE = Empirical Standard Error; RMSE = Root Mean Square Error; ModSE = Model-based Standard Error; Corr: Correlation; ID = Intervention deviation.

Table C5. Results for IPCW with too few TVCs: IPCW with stabilised weight and omitting L2 and IPCW with stabilised weight and omitting L1 (IPCWsL1 and IPCWsL2).

Scenario			Performance measure					
**No.**	**True**	**Description**	**Mean**	**Bias**	**EmpSE**	**RMSE**	**ModSE**	**Coverage(%)**
IPCWsL1								
1	-0.080	Base	-0.074	0.005	0.027	0.028	0.027	93.80
2	-0.077	Corr: Low	-0.070	0.006	0.025	0.026	0.026	94.20
3	-0.082	Corr: High	-0.079	0.003	0.028	0.028	0.029	94.80
4	-0.080	ID prevalence: Low	-0.077	0.002	0.023	0.023	0.024	96.50
5	-0.080	ID prevalence: High	-0.069	0.010	0.041	0.042	0.037	89.00
6	-0.080	Confounder-ID Magnitude: Low	-0.074	0.005	0.025	0.026	0.026	94.70
7	-0.080	Confounder-ID Magnitude: High	-0.074	0.006	0.027	0.028	0.028	93.90
8	-0.080	Confounder-ID Sign: $-/+$	-0.076	0.003	0.024	0.024	0.025	95.30
9	-0.080	Deterministic ID	-0.060	0.019	0.034	0.039	0.034	83.80
10	-0.080	Both-arm ID, arm-ID:0	-0.077	0.002	0.028	0.028	0.029	95.20
11	-0.080	Both-arm ID, arm-ID:$-$	-0.076	0.003	0.028	0.028	0.028	94.30
12	-0.080	Both-arm ID, arm-ID:$+$	-0.078	0.001	0.029	0.029	0.030	96.00
13	-0.080	Arm-outcome: Low	-0.051	0.006	0.028	0.029	0.028	93.20
14	-0.104	Arm-outcome: High	-0.099	0.005	0.027	0.027	0.027	93.80
15	-0.087	Confounder-outcome: Low	-0.081	0.006	0.029	0.029	0.029	94.30
16	-0.075	Confounder-outcome: High	-0.069	0.006	0.025	0.026	0.026	94.20
17	-0.080	Sample size: 200	-0.070	0.009	0.061	0.062	0.060	92.99
18	-0.080	Sample size: 500	-0.074	0.006	0.039	0.040	0.039	95.00
IPCWsL2								
1	-0.080	Base	-0.075	0.004	0.027	0.028	0.028	94.40
2	-0.077	Corr: Low	-0.072	0.004	0.026	0.027	0.026	94.70
3	-0.082	Corr: High	-0.080	0.003	0.030	0.030	0.031	94.10
4	-0.080	ID prevalence: Low	-0.078	0.002	0.024	0.024	0.024	96.40
5	-0.080	ID prevalence: High	-0.071	0.009	0.041	0.042	0.038	89.89
6	-0.080	Confounder-ID Magnitude: Low	-0.077	0.002	0.026	0.026	0.027	95.80
7	-0.080	Confounder-ID Magnitude: High	-0.073	0.006	0.027	0.027	0.027	93.40
8	-0.080	Confounder-ID Sign: $-/+$	-0.084	-0.005	0.025	0.026	0.026	95.10
9	-0.080	Deterministic ID	-0.061	0.019	0.033	0.038	0.033	84.45
10	-0.080	Both-arm ID, arm-ID:0	-0.078	0.001	0.029	0.029	0.029	95.20
11	-0.080	Both-arm ID, arm-ID:$-$	-0.077	0.002	0.028	0.028	0.028	94.00
12	-0.080	Both-arm ID, arm-ID:$+$	-0.079	0.001	0.031	0.031	0.031	95.90
13	-0.080	Arm-outcome: Low	-0.052	0.004	0.028	0.029	0.028	93.40
14	-0.104	Arm-outcome: High	-0.101	0.004	0.027	0.027	0.027	93.80
15	-0.087	Confounder-outcome: Low	-0.085	0.002	0.030	0.030	0.030	94.80
16	-0.075	Confounder-outcome: High	-0.068	0.006	0.025	0.026	0.026	93.20
17	-0.080	Sample size: 200	-0.072	0.007	0.065	0.065	0.060	92.60
18	-0.080	Sample size: 500	-0.075	0.005	0.038	0.038	0.039	95.30
Largest MCSE			0.002	0.002	0.001	0.003	0.001	1.17

Note: MCSE = Monte Carlo Standard Error; EmpSE = Empirical Standard Error; RMSE = Root Mean Square Error; ModSE = Model-based Standard Error; Corr: Correlation; ID = Intervention deviation.

Table C6. Results for IPCW with mis-specified TVCs: IPCW with stabilised weight, omitting L2 and additional including L3, L4 and L5 (IPCWsL1L3L4L5).

Scenario			Performance measure					
**No.**	**True**	**Description**	**Mean**	**Bias**	**EmpSE**	**RMSE**	**ModSE**	**Coverage(%)**
IPCWsL1L3L4L5								
1	-0.080	Base	-0.073	0.006	0.027	0.028	0.027	93.30
2	-0.077	Corr: Low	-0.070	0.006	0.025	0.026	0.025	93.99
3	-0.082	Corr: High	-0.079	0.004	0.029	0.029	0.030	94.30
4	-0.080	ID prevalence: Low	-0.077	0.002	0.023	0.024	0.024	96.40
5	-0.080	ID prevalence: High	-0.068	0.011	0.039	0.040	0.037	88.40
6	-0.080	Confounder-ID Magnitude: Low	-0.074	0.005	0.025	0.026	0.026	94.10
7	-0.080	Confounder-ID Magnitude: High	-0.073	0.006	0.027	0.028	0.028	94.00
8	-0.080	Confounder-ID Sign: $-/+$	-0.076	0.003	0.024	0.024	0.025	95.40
9	-0.080	Deterministic ID	-0.060	0.020	0.033	0.038	0.033	83.80
10	-0.080	Both-arm ID, arm-ID:0	-0.077	0.003	0.028	0.028	0.029	95.20
11	-0.080	Both-arm ID, arm-ID:$-$	-0.076	0.003	0.028	0.028	0.028	94.40
12	-0.080	Both-arm ID, arm-ID:$+$	-0.078	0.001	0.030	0.030	0.030	95.70
13	-0.080	Arm-outcome: Low	-0.050	0.006	0.028	0.029	0.028	93.10
14	-0.104	Arm-outcome: High	-0.099	0.006	0.026	0.027	0.026	93.90
15	-0.087	Confounder-outcome: Low	-0.081	0.006	0.029	0.029	0.029	94.20
16	-0.075	Confounder-outcome: High	-0.068	0.006	0.025	0.026	0.026	94.00
17	-0.080	Sample size: 200	-0.070	0.009	0.061	0.062	0.060	93.30
18	-0.080	Sample size: 500	-0.074	0.006	0.039	0.040	0.039	95.20
Largest MCSE			0.002	0.002	0.001	0.002	0.001	1.17

Note: MCSE = Monte Carlo Standard Error; EmpSE = Empirical Standard Error; RMSE = Root Mean Square Error; ModSE = Model-based Standard Error; Corr: Correlation; ID = Intervention deviation.

Table C7. Results for IPCW with unnecessary TVC: IPCW with stabilised weight, correctly including L1 and L2 and additional including L3, L4 and L5 (IPCWsL1L2L3L4L5).

**Scenario**			**Performance measure**					
**No.**	**True**	**Description**	**Mean**	**Bias**	**EmpSE**	**RMSE**	**ModSE**	**Coverage(%)**
IPCWsL1L2L3L4L5								
1	-0.080	Base	-0.080	-0.000	0.028	0.028	0.029	94.30
2	-0.077	Corr: Low	-0.076	-0.000	0.027	0.027	0.027	95.50
3	-0.082	Corr: High	-0.083	-0.000	0.030	0.030	0.031	95.10
4	-0.080	ID prevalence: Low	-0.080	-0.001	0.024	0.024	0.025	96.40
5	-0.080	ID prevalence: High	-0.080	-0.001	0.044	0.044	0.041	92.69
6	-0.080	Confounder-ID Magnitude: Low	-0.079	-0.000	0.026	0.026	0.027	95.70
7	-0.080	Confounder-ID Magnitude: High	-0.080	-0.000	0.029	0.029	0.029	94.40
8	-0.080	Confounder-ID Sign: -/+	-0.081	-0.001	0.025	0.025	0.026	95.40
9	-0.080	Deterministic ID	-0.065	0.015	0.031	0.034	0.032	88.49
10	-0.080	Both-arm ID, arm-ID:0	-0.080	-0.001	0.030	0.030	0.031	95.40
11	-0.080	Both-arm ID, arm-ID:-	-0.080	-0.001	0.030	0.030	0.030	94.40
12	-0.080	Both-arm ID, arm-ID:+	-0.080	-0.001	0.032	0.032	0.032	95.80
13	-0.080	Arm-outcome: Low	-0.056	-0.000	0.029	0.029	0.029	93.70
14	-0.104	Arm-outcome: High	-0.105	-0.001	0.028	0.028	0.028	93.90
15	-0.087	Confounder-outcome: Low	-0.088	-0.001	0.030	0.030	0.031	95.10
16	-0.075	Confounder-outcome: High	-0.074	0.000	0.026	0.026	0.027	94.80
17	-0.080	Sample size: 200	-0.076	0.003	0.065	0.065	0.062	93.39
18	-0.080	Sample size: 500	-0.080	-0.000	0.040	0.040	0.041	95.60
Largest MCSE			0.002	0.002	0.001	0.002	0.001	1.01

Note: MCSE = Monte Carlo Standard Error; EmpSE = Empirical Standard Error; RMSE = Root Mean Square Error; ModSE = Model-based Standard Error; Corr: Correlation; ID = Intervention deviation.

Table C8. Results for IPCW with measurement error: IPCW with stabilised weight and L1 and L2 measured with errors (IPCWsL1*L2*).

Scenario			Performance measure					
**No.**	**True**	**Description**	**Mean**	**Bias**	**EmpSE**	**RMSE**	**ModSE**	**Coverage(%)**
*IPCWsL1*L2**								
1	-0.080	Base	-0.072	0.007	0.026	0.027	0.026	93.90
2	-0.077	Corr: Low	-0.071	0.005	0.025	0.025	0.025	94.40
3	-0.082	Corr: High	-0.074	0.009	0.026	0.027	0.027	92.60
4	-0.080	ID prevalence: Low	-0.077	0.003	0.023	0.023	0.024	96.50
5	-0.080	ID prevalence: High	-0.066	0.014	0.036	0.038	0.034	87.90
6	-0.080	Confounder-ID Magnitude: Low	-0.074	0.006	0.025	0.026	0.026	94.50
7	-0.080	Confounder-ID Magnitude: High	-0.072	0.008	0.025	0.026	0.026	94.00
8	-0.080	Confounder-ID Sign: -/+	-0.077	0.002	0.024	0.024	0.025	95.10
9	-0.080	Deterministic ID	-0.058	0.022	0.026	0.034	0.027	84.40
10	-0.080	Both-arm ID, arm-ID:0	-0.077	0.003	0.027	0.027	0.027	95.00
11	-0.080	Both-arm ID, arm-ID:-	-0.075	0.004	0.026	0.027	0.027	94.40
12	-0.080	Both-arm ID, arm-ID:+	-0.078	0.001	0.028	0.028	0.028	95.70
13	-0.080	Arm-outcome: Low	-0.049	0.007	0.027	0.028	0.027	92.90
14	-0.104	Arm-outcome: High	-0.098	0.007	0.025	0.026	0.025	93.80
15	-0.087	Confounder-outcome: Low	-0.082	0.005	0.028	0.028	0.029	94.70
16	-0.075	Confounder-outcome: High	-0.066	0.008	0.023	0.025	0.024	93.40
17	-0.080	Sample size: 200	-0.069	0.010	0.061	0.062	0.059	92.80
18	-0.080	Sample size: 500	-0.072	0.008	0.036	0.037	0.037	94.60
Largest MCSE			0.002	0.002	0.001	0.002	0.001	1.15

Note: MCSE = Monte Carlo Standard Error; EmpSE = Empirical Standard Error; RMSE = Root Mean Square Error; ModSE = Model-based Standard Error; Corr: Correlation; ID = Intervention deviation.

C.4 Summary of performance measures under investigation for each scenario

Results of all performance measures under investigation described in Section 3.5 for all scenarios are shown in the following tables.

Table C9. Table of simsum results for scenario 1

	Mean Estimate	MCSE	Bias	MCSE	EmpSE	MCSE	RMSE	MCSE	ModSE	MCSE	Coverage(%)	MCSE
ITT	-0.067	0.001	0.012	0.001	0.021	0.000	0.025	0.000	0.022	0.000	92.60	0.83
PP	-0.062	0.001	0.018	0.001	0.023	0.001	0.029	0.001	0.024	0.000	87.90	1.03
IPCWuL2	-0.074	0.001	0.005	0.001	0.027	0.001	0.028	0.001	0.028	0.000	94.00	0.75
IPCWsL2	-0.075	0.001	0.004	0.001	0.027	0.001	0.028	0.001	0.028	0.000	94.40	0.73
IPCWuL1	-0.073	0.001	0.006	0.001	0.027	0.001	0.028	0.001	0.027	0.000	93.60	0.77
IPCWsL1	-0.074	0.001	0.005	0.001	0.027	0.001	0.028	0.001	0.027	0.000	93.80	0.76
IPCWuL1L3	-0.073	0.001	0.006	0.001	0.027	0.001	0.028	0.001	0.027	0.000	93.60	0.77
IPCWsL1L3	-0.074	0.001	0.005	0.001	0.027	0.001	0.028	0.001	0.027	0.000	94.00	0.75
IPCWuL1L4	-0.073	0.001	0.007	0.001	0.027	0.001	0.028	0.001	0.027	0.000	93.20	0.80
IPCWsL1L4	-0.074	0.001	0.006	0.001	0.027	0.001	0.028	0.001	0.027	0.000	93.50	0.78
IPCWuL1L4L5	-0.072	0.001	0.007	0.001	0.027	0.001	0.028	0.001	0.027	0.000	93.00	0.81
IPCWsL1L4L5	-0.073	0.001	0.006	0.001	0.027	0.001	0.028	0.001	0.027	0.000	93.40	0.79
IPCWuL1L3L4L5	-0.073	0.001	0.007	0.001	0.027	0.001	0.028	0.001	0.027	0.000	93.20	0.80
IPCWsL1L3L4L5	-0.073	0.001	0.006	0.001	0.027	0.001	0.028	0.001	0.027	0.000	93.30	0.79
IPCWuLb1Lb2	-0.065	0.001	0.014	0.001	0.025	0.001	0.029	0.001	0.025	0.000	90.30	0.94
IPCWsLb1Lb2	-0.066	0.001	0.014	0.001	0.025	0.001	0.029	0.001	0.026	0.000	90.80	0.91
IPCWuL1*	-0.068	0.001	0.012	0.001	0.025	0.001	0.028	0.001	0.026	0.000	92.10	0.85
IPCWsL1*	-0.069	0.001	0.011	0.001	0.025	0.001	0.027	0.001	0.026	0.000	92.60	0.83
IPCWuL1L2	-0.080	0.001	-0.000	0.001	0.029	0.001	0.029	0.001	0.029	0.001	94.50	0.72
IPCWsL1L2	-0.080	0.001	-0.001	0.001	0.029	0.001	0.029	0.001	0.029	0.001	94.40	0.73
IPCWuL1L2L3	-0.079	0.001	-0.000	0.001	0.029	0.001	0.029	0.001	0.029	0.000	94.40	0.73
IPCWsL1L2L3	-0.080	0.001	-0.001	0.001	0.029	0.001	0.029	0.001	0.029	0.001	94.30	0.73
IPCWuL1L2L4	-0.079	0.001	-0.000	0.001	0.029	0.001	0.029	0.001	0.029	0.000	94.10	0.75
IPCWsL1L2L4	-0.080	0.001	-0.001	0.001	0.029	0.001	0.029	0.001	0.029	0.000	94.20	0.74
IPCWuL1L2L4L5	-0.079	0.001	0.001	0.001	0.028	0.001	0.028	0.001	0.029	0.000	94.30	0.73
IPCWsL1L2L4L5	-0.080	0.001	-0.000	0.001	0.028	0.001	0.028	0.001	0.029	0.000	94.20	0.74
IPCWuL1L2L3L4L5	-0.079	0.001	0.001	0.001	0.028	0.001	0.028	0.001	0.029	0.000	94.20	0.74
IPCWsL1L2L3L4L5	-0.080	0.001	-0.000	0.001	0.028	0.001	0.028	0.001	0.029	0.000	94.30	0.73
IPCWuL1*L2*	-0.072	0.001	0.008	0.001	0.026	0.001	0.027	0.001	0.026	0.000	93.50	0.78
IPCWsL1*L2*	-0.072	0.001	0.007	0.001	0.026	0.001	0.027	0.001	0.026	0.000	93.90	0.76

Table C10. Table of simsum results for scenario 2

	Mean Estimate	MCSE	Bias	MCSE	EmpSE	MCSE	RMSE	MCSE	ModSE	MCSE	Coverage(%)	MCSE
ITT	-0.065	0.001	0.011	0.001	0.021	0.000	0.023	0.000	0.022	0.000	93.40	0.79
PP	-0.063	0.001	0.013	0.001	0.023	0.001	0.026	0.001	0.023	0.000	90.50	0.93
IPCWuL2	-0.071	0.001	0.005	0.001	0.026	0.001	0.027	0.001	0.026	0.000	94.10	0.75
IPCWsL2	-0.072	0.001	0.004	0.001	0.026	0.001	0.027	0.001	0.026	0.000	94.70	0.71
IPCWuL1	-0.070	0.001	0.006	0.001	0.025	0.001	0.026	0.001	0.026	0.000	93.70	0.77
IPCWsL1	-0.070	0.001	0.006	0.001	0.025	0.001	0.026	0.001	0.026	0.000	94.20	0.74
IPCWuL1L3	-0.070	0.001	0.006	0.001	0.025	0.001	0.026	0.001	0.026	0.000	93.80	0.76
IPCWsL1L3	-0.070	0.001	0.005	0.001	0.025	0.001	0.026	0.001	0.026	0.000	94.10	0.75
IPCWuL1L4	-0.069	0.001	0.007	0.001	0.025	0.001	0.026	0.001	0.026	0.000	93.60	0.77
IPCWsL1L4	-0.070	0.001	0.006	0.001	0.025	0.001	0.026	0.001	0.026	0.000	93.70	0.77
IPCWuL1L4L5	-0.069	0.001	0.007	0.001	0.025	0.001	0.026	0.001	0.025	0.000	93.59	0.78
IPCWsL1L4L5	-0.070	0.001	0.006	0.001	0.025	0.001	0.026	0.001	0.025	0.000	93.79	0.76
IPCWuL1L3L4L5	-0.069	0.001	0.007	0.001	0.025	0.001	0.026	0.001	0.025	0.000	93.49	0.78
IPCWsL1L3L4L5	-0.070	0.001	0.006	0.001	0.025	0.001	0.026	0.001	0.025	0.000	93.99	0.75
IPCWuLb1Lb2	-0.066	0.001	0.010	0.001	0.024	0.001	0.026	0.001	0.025	0.000	91.70	0.87
IPCWsLb1Lb2	-0.067	0.001	0.009	0.001	0.024	0.001	0.026	0.001	0.025	0.000	92.60	0.83
IPCWuL1*	-0.067	0.001	0.009	0.001	0.024	0.001	0.026	0.001	0.025	0.000	92.40	0.84
IPCWsL1*	-0.067	0.001	0.009	0.001	0.024	0.001	0.026	0.001	0.025	0.000	93.20	0.80
IPCWuL1L2	-0.076	0.001	-0.000	0.001	0.027	0.001	0.027	0.001	0.027	0.000	94.90	0.70
IPCWsL1L2	-0.077	0.001	-0.001	0.001	0.027	0.001	0.027	0.001	0.027	0.000	94.80	0.70
IPCWuL1L2L3	-0.076	0.001	-0.000	0.001	0.027	0.001	0.027	0.001	0.027	0.000	94.90	0.70
IPCWsL1L2L3	-0.077	0.001	-0.001	0.001	0.027	0.001	0.027	0.001	0.027	0.000	95.10	0.68
IPCWuL1L2L4	-0.076	0.001	-0.000	0.001	0.027	0.001	0.027	0.001	0.027	0.000	95.40	0.66
IPCWsL1L2L4	-0.077	0.001	-0.001	0.001	0.027	0.001	0.027	0.001	0.027	0.000	95.40	0.66
IPCWuL1L2L4L5	-0.076	0.001	0.000	0.001	0.026	0.001	0.026	0.001	0.027	0.000	95.60	0.65
IPCWsL1L2L4L5	-0.076	0.001	-0.001	0.001	0.027	0.001	0.027	0.001	0.027	0.000	95.50	0.66
IPCWuL1L2L3L4L5	-0.076	0.001	0.000	0.001	0.026	0.001	0.026	0.001	0.027	0.000	95.70	0.64
IPCWsL1L2L3L4L5	-0.076	0.001	-0.000	0.001	0.027	0.001	0.027	0.001	0.027	0.000	95.50	0.66
IPCWuL1*L2*	-0.070	0.001	0.006	0.001	0.025	0.001	0.025	0.001	0.025	0.000	94.30	0.73
IPCWsL1*L2*	-0.071	0.001	0.005	0.001	0.025	0.001	0.025	0.001	0.025	0.000	94.40	0.73

Table C11. Table of simsum results for scenario 3

	Mean Estimate	MCSE	Bias	MCSE	EmpSE	MCSE	RMSE	MCSE	ModSE	MCSE	Coverage(%)	MCSE
ITT	-0.069	0.001	0.014	0.001	0.022	0.000	0.026	0.001	0.023	0.000	91.90	0.86
PP	-0.060	0.001	0.023	0.001	0.023	0.001	0.032	0.001	0.024	0.000	84.90	1.13
IPCWuL2	-0.079	0.001	0.003	0.001	0.030	0.001	0.030	0.001	0.030	0.001	93.60	0.77
IPCWsL2	-0.080	0.001	0.003	0.001	0.030	0.001	0.030	0.001	0.031	0.001	94.10	0.75
IPCWuL1	-0.078	0.001	0.004	0.001	0.028	0.001	0.028	0.001	0.029	0.000	94.10	0.75
IPCWsL1	-0.079	0.001	0.003	0.001	0.028	0.001	0.028	0.001	0.029	0.000	94.80	0.70
IPCWuL1L3	-0.078	0.001	0.004	0.001	0.028	0.001	0.028	0.001	0.029	0.000	94.40	0.73
IPCWsL1L3	-0.079	0.001	0.004	0.001	0.028	0.001	0.028	0.001	0.029	0.000	94.90	0.70
IPCWuL1L4	-0.078	0.001	0.004	0.001	0.029	0.001	0.029	0.001	0.030	0.001	94.00	0.75
IPCWsL1L4	-0.079	0.001	0.003	0.001	0.029	0.001	0.029	0.001	0.030	0.001	94.20	0.74
IPCWuL1L4L5	-0.078	0.001	0.005	0.001	0.029	0.001	0.029	0.001	0.030	0.001	93.90	0.76
IPCWsL1L4L5	-0.079	0.001	0.004	0.001	0.029	0.001	0.029	0.001	0.030	0.001	94.40	0.73
IPCWuL1L3L4L5	-0.078	0.001	0.005	0.001	0.029	0.001	0.029	0.001	0.030	0.001	94.10	0.75
IPCWsL1L3L4L5	-0.079	0.001	0.004	0.001	0.029	0.001	0.029	0.001	0.030	0.001	94.30	0.73
IPCWuLb1Lb2	-0.064	0.001	0.019	0.001	0.026	0.001	0.032	0.001	0.027	0.000	86.60	1.08
IPCWsLb1Lb2	-0.064	0.001	0.018	0.001	0.026	0.001	0.032	0.001	0.027	0.000	87.20	1.06
IPCWuL1*	-0.069	0.001	0.013	0.001	0.025	0.001	0.029	0.001	0.026	0.000	91.40	0.89
IPCWsL1*	-0.070	0.001	0.012	0.001	0.025	0.001	0.028	0.001	0.027	0.000	91.70	0.87
IPCWuL1L2	-0.082	0.001	0.000	0.001	0.030	0.001	0.030	0.001	0.031	0.001	94.60	0.71
IPCWsL1L2	-0.083	0.001	-0.001	0.001	0.030	0.001	0.030	0.001	0.031	0.001	95.00	0.69
IPCWuL1L2L3	-0.082	0.001	0.000	0.001	0.030	0.001	0.030	0.001	0.031	0.001	94.60	0.71
IPCWsL1L2L3	-0.083	0.001	-0.001	0.001	0.030	0.001	0.030	0.001	0.031	0.001	94.80	0.70
IPCWuL1L2L4	-0.082	0.001	0.000	0.001	0.031	0.001	0.031	0.001	0.032	0.001	94.00	0.75
IPCWsL1L2L4	-0.083	0.001	-0.001	0.001	0.031	0.001	0.031	0.001	0.032	0.001	94.80	0.70
IPCWuL1L2L4L5	-0.082	0.001	0.001	0.001	0.030	0.001	0.030	0.001	0.031	0.001	95.10	0.68
IPCWsL1L2L4L5	-0.083	0.001	-0.000	0.001	0.030	0.001	0.030	0.001	0.031	0.001	95.10	0.68
IPCWuL1L2L3L4L5	-0.082	0.001	0.001	0.001	0.030	0.001	0.030	0.001	0.031	0.001	95.20	0.68
IPCWsL1L2L3L4L5	-0.083	0.001	-0.000	0.001	0.030	0.001	0.030	0.001	0.031	0.001	95.10	0.68
IPCWuL1*L2*	-0.073	0.001	0.010	0.001	0.026	0.001	0.028	0.001	0.027	0.000	92.10	0.85
IPCWsL1*L2*	-0.074	0.001	0.009	0.001	0.026	0.001	0.027	0.001	0.027	0.000	92.60	0.83

Table C12. Table of simsum results for scenario 4

	Mean Estimate	MCSE	Bias	MCSE	EmpSE	MCSE	RMSE	MCSE	ModSE	MCSE	Coverage(%)	MCSE
ITT	-0.074	0.001	0.006	0.001	0.022	0.000	0.022	0.000	0.023	0.000	96.10	0.61
PP	-0.070	0.001	0.009	0.001	0.022	0.000	0.024	0.000	0.023	0.000	93.90	0.76
IPCWuL2	-0.077	0.001	0.002	0.001	0.024	0.001	0.024	0.001	0.024	0.000	96.60	0.57
IPCWsL2	-0.078	0.001	0.002	0.001	0.024	0.001	0.024	0.001	0.024	0.000	96.40	0.59
IPCWuL1	-0.077	0.001	0.003	0.001	0.023	0.001	0.023	0.000	0.024	0.000	96.30	0.60
IPCWsL1	-0.077	0.001	0.002	0.001	0.023	0.001	0.023	0.000	0.024	0.000	96.50	0.58
IPCWuL1L3	-0.077	0.001	0.003	0.001	0.023	0.001	0.023	0.000	0.024	0.000	96.50	0.58
IPCWsL1L3	-0.077	0.001	0.002	0.001	0.023	0.001	0.023	0.000	0.024	0.000	96.50	0.58
IPCWuL1L4	-0.077	0.001	0.003	0.001	0.023	0.001	0.024	0.000	0.024	0.000	96.60	0.57
IPCWsL1L4	-0.077	0.001	0.002	0.001	0.023	0.001	0.024	0.000	0.024	0.000	96.60	0.57
IPCWuL1L4L5	-0.077	0.001	0.003	0.001	0.023	0.001	0.024	0.000	0.024	0.000	96.10	0.61
IPCWsL1L4L5	-0.077	0.001	0.002	0.001	0.023	0.001	0.024	0.000	0.024	0.000	96.50	0.58
IPCWuL1L3L4L5	-0.077	0.001	0.003	0.001	0.023	0.001	0.024	0.000	0.024	0.000	96.00	0.62
IPCWsL1L3L4L5	-0.077	0.001	0.002	0.001	0.023	0.001	0.024	0.000	0.024	0.000	96.40	0.59
IPCWuLb1Lb2	-0.073	0.001	0.007	0.001	0.023	0.001	0.024	0.000	0.024	0.000	94.70	0.71
IPCWsLb1Lb2	-0.073	0.001	0.006	0.001	0.023	0.001	0.024	0.000	0.024	0.000	94.90	0.70
IPCWuL1*	-0.074	0.001	0.005	0.001	0.023	0.001	0.023	0.000	0.024	0.000	95.80	0.63
IPCWsL1*	-0.075	0.001	0.005	0.001	0.023	0.001	0.023	0.000	0.024	0.000	95.90	0.63
IPCWuL1L2	-0.080	0.001	-0.001	0.001	0.024	0.001	0.024	0.001	0.025	0.000	96.60	0.57
IPCWsL1L2	-0.080	0.001	-0.001	0.001	0.024	0.001	0.024	0.001	0.025	0.000	96.60	0.57
IPCWuL1L2L3	-0.080	0.001	-0.001	0.001	0.024	0.001	0.024	0.001	0.025	0.000	96.40	0.59
IPCWsL1L2L3	-0.080	0.001	-0.001	0.001	0.024	0.001	0.024	0.001	0.025	0.000	96.40	0.59
IPCWuL1L2L4	-0.080	0.001	-0.001	0.001	0.024	0.001	0.024	0.001	0.025	0.000	96.30	0.60
IPCWsL1L2L4	-0.080	0.001	-0.001	0.001	0.024	0.001	0.024	0.001	0.025	0.000	96.30	0.60
IPCWuL1L2L4L5	-0.080	0.001	-0.000	0.001	0.024	0.001	0.024	0.001	0.025	0.000	96.30	0.60
IPCWsL1L2L4L5	-0.080	0.001	-0.001	0.001	0.024	0.001	0.024	0.001	0.025	0.000	96.40	0.59
IPCWuL1L2L3L4L5	-0.080	0.001	-0.000	0.001	0.024	0.001	0.024	0.001	0.025	0.000	96.30	0.60
IPCWsL1L2L3L4L5	-0.080	0.001	-0.001	0.001	0.024	0.001	0.024	0.001	0.025	0.000	96.40	0.59
IPCWuL1*L2*	-0.076	0.001	0.003	0.001	0.023	0.001	0.023	0.000	0.024	0.000	96.30	0.60
IPCWsL1*L2*	-0.077	0.001	0.003	0.001	0.023	0.001	0.023	0.000	0.024	0.000	96.50	0.58

Table C13. Table of simsum results for scenario 5

	Mean Estimate	MCSE	Bias	MCSE	EmpSE	MCSE	RMSE	MCSE	ModSE	MCSE	Coverage(%)	MCSE
ITT	-0.057	0.001	0.022	0.001	0.021	0.000	0.030	0.001	0.022	0.000	83.90	1.16
PP	-0.049	0.001	0.030	0.001	0.025	0.001	0.039	0.001	0.025	0.000	75.90	1.35
IPCWuL2	-0.069	0.001	0.011	0.001	0.041	0.001	0.042	0.002	0.038	0.001	88.09	1.02
IPCWsL2	-0.071	0.001	0.009	0.001	0.041	0.001	0.042	0.002	0.038	0.001	89.89	0.95
IPCWuL1	-0.067	0.001	0.013	0.001	0.040	0.001	0.042	0.002	0.037	0.001	87.10	1.06
IPCWsL1	-0.069	0.001	0.010	0.001	0.041	0.001	0.042	0.002	0.037	0.001	89.00	0.99
IPCWuL1L3	-0.067	0.001	0.013	0.001	0.040	0.001	0.042	0.002	0.037	0.001	87.30	1.05
IPCWsL1L3	-0.069	0.001	0.010	0.001	0.040	0.001	0.042	0.002	0.037	0.001	89.20	0.98
IPCWuL1L4	-0.066	0.001	0.013	0.001	0.039	0.001	0.041	0.002	0.036	0.001	86.69	1.07
IPCWsL1L4	-0.068	0.001	0.011	0.001	0.039	0.001	0.041	0.002	0.037	0.001	88.70	1.00
IPCWuL1L4L5	-0.066	0.001	0.014	0.001	0.039	0.001	0.041	0.002	0.036	0.001	86.90	1.07
IPCWsL1L4L5	-0.068	0.001	0.012	0.001	0.039	0.001	0.041	0.002	0.037	0.001	88.20	1.02
IPCWuL1L3L4L5	-0.066	0.001	0.013	0.001	0.039	0.001	0.041	0.002	0.036	0.001	87.09	1.06
IPCWsL1L3L4L5	-0.068	0.001	0.011	0.001	0.039	0.001	0.040	0.002	0.037	0.001	88.40	1.01
IPCWuLb1Lb2	-0.053	0.001	0.026	0.001	0.036	0.001	0.044	0.001	0.033	0.001	77.10	1.33
IPCWsLb1Lb2	-0.055	0.001	0.024	0.001	0.036	0.001	0.043	0.001	0.033	0.001	78.60	1.30
IPCWuL1*	-0.057	0.001	0.023	0.001	0.033	0.001	0.040	0.001	0.031	0.001	81.50	1.23
IPCWsL1*	-0.059	0.001	0.021	0.001	0.033	0.001	0.039	0.001	0.032	0.001	83.50	1.17
IPCWuL1L2	-0.078	0.001	0.001	0.001	0.045	0.001	0.045	0.002	0.041	0.001	92.49	0.83
IPCWsL1L2	-0.081	0.001	-0.002	0.001	0.045	0.001	0.045	0.002	0.042	0.001	92.99	0.81
IPCWuL1L2L3	-0.078	0.001	0.001	0.001	0.045	0.001	0.045	0.002	0.041	0.001	92.29	0.84
IPCWsL1L2L3	-0.081	0.001	-0.002	0.001	0.045	0.001	0.045	0.002	0.042	0.001	92.79	0.82
IPCWuL1L2L4	-0.078	0.001	0.002	0.001	0.043	0.001	0.043	0.002	0.040	0.001	92.09	0.85
IPCWsL1L2L4	-0.080	0.001	-0.001	0.001	0.044	0.001	0.044	0.002	0.041	0.001	92.99	0.81
IPCWuL1L2L4L5	-0.077	0.001	0.002	0.001	0.043	0.001	0.043	0.002	0.040	0.001	92.29	0.84
IPCWsL1L2L4L5	-0.080	0.001	-0.001	0.001	0.044	0.001	0.044	0.002	0.041	0.001	93.09	0.80
IPCWuL1L2L3L4L5	-0.078	0.001	0.002	0.001	0.043	0.001	0.043	0.002	0.041	0.001	92.09	0.85
IPCWsL1L2L3L4L5	-0.080	0.001	-0.001	0.001	0.044	0.001	0.044	0.002	0.041	0.001	92.69	0.82
IPCWuL1*L2*	-0.063	0.001	0.016	0.001	0.036	0.001	0.039	0.002	0.033	0.001	86.50	1.08
IPCWsL1*L2*	-0.066	0.001	0.014	0.001	0.036	0.001	0.038	0.002	0.034	0.001	87.90	1.03

Table C14. Table of simsum results for scenario 6

	Mean Estimate	MCSE	Bias	MCSE	EmpSE	MCSE	RMSE	MCSE	ModSE	MCSE	Coverage(%)	MCSE
ITT	-0.068	0.001	0.011	0.001	0.021	0.000	0.024	0.000	0.022	0.000	93.90	0.76
PP	-0.066	0.001	0.014	0.001	0.023	0.001	0.027	0.001	0.024	0.000	91.10	0.90
IPCWuL2	-0.077	0.001	0.003	0.001	0.026	0.001	0.026	0.001	0.027	0.000	95.50	0.66
IPCWsL2	-0.077	0.001	0.002	0.001	0.026	0.001	0.026	0.001	0.027	0.000	95.80	0.63
IPCWuL1	-0.074	0.001	0.006	0.001	0.025	0.001	0.026	0.001	0.026	0.000	94.30	0.73
IPCWsL1	-0.074	0.001	0.005	0.001	0.025	0.001	0.026	0.001	0.026	0.000	94.70	0.71
IPCWuL1L3	-0.074	0.001	0.006	0.001	0.025	0.001	0.026	0.001	0.026	0.000	94.40	0.73
IPCWsL1L3	-0.074	0.001	0.005	0.001	0.025	0.001	0.026	0.001	0.026	0.000	94.60	0.71
IPCWuL1L4	-0.073	0.001	0.006	0.001	0.025	0.001	0.026	0.001	0.026	0.000	93.80	0.76
IPCWsL1L4	-0.074	0.001	0.005	0.001	0.025	0.001	0.026	0.001	0.026	0.000	94.30	0.73
IPCWuL1L4L5	-0.073	0.001	0.006	0.001	0.025	0.001	0.026	0.001	0.026	0.000	93.70	0.77
IPCWsL1L4L5	-0.074	0.001	0.005	0.001	0.025	0.001	0.026	0.001	0.026	0.000	94.20	0.74
IPCWuL1L3L4L5	-0.073	0.001	0.006	0.001	0.025	0.001	0.026	0.001	0.026	0.000	93.70	0.77
IPCWsL1L3L4L5	-0.074	0.001	0.005	0.001	0.025	0.001	0.026	0.001	0.026	0.000	94.10	0.75
IPCWuLb1Lb2	-0.068	0.001	0.011	0.001	0.024	0.001	0.027	0.001	0.025	0.000	91.90	0.86
IPCWsLb1Lb2	-0.069	0.001	0.010	0.001	0.025	0.001	0.026	0.001	0.025	0.000	92.60	0.83
IPCWuL1*	-0.069	0.001	0.010	0.001	0.024	0.001	0.026	0.001	0.025	0.000	92.80	0.82
IPCWsL1*	-0.070	0.001	0.009	0.001	0.024	0.001	0.026	0.001	0.025	0.000	93.00	0.81
IPCWuL1L2	-0.079	0.001	0.000	0.001	0.026	0.001	0.026	0.001	0.027	0.000	95.40	0.66
IPCWsL1L2	-0.080	0.001	-0.001	0.001	0.027	0.001	0.027	0.001	0.027	0.000	95.60	0.65
IPCWuL1L2L3	-0.079	0.001	0.000	0.001	0.026	0.001	0.026	0.001	0.027	0.000	95.30	0.67
IPCWsL1L2L3	-0.080	0.001	-0.001	0.001	0.027	0.001	0.027	0.001	0.027	0.000	95.80	0.63
IPCWuL1L2L4	-0.079	0.001	0.000	0.001	0.026	0.001	0.026	0.001	0.027	0.000	95.70	0.64
IPCWsL1L2L4	-0.080	0.001	-0.000	0.001	0.026	0.001	0.026	0.001	0.027	0.000	95.80	0.63
IPCWuL1L2L4L5	-0.079	0.001	0.001	0.001	0.026	0.001	0.026	0.001	0.027	0.000	95.60	0.65
IPCWsL1L2L4L5	-0.079	0.001	-0.000	0.001	0.026	0.001	0.026	0.001	0.027	0.000	95.60	0.65
IPCWuL1L2L3L4L5	-0.078	0.001	0.001	0.001	0.026	0.001	0.026	0.001	0.027	0.000	95.70	0.64
IPCWsL1L2L3L4L5	-0.079	0.001	-0.000	0.001	0.026	0.001	0.026	0.001	0.027	0.000	95.70	0.64
IPCWuL1*L2*	-0.073	0.001	0.006	0.001	0.025	0.001	0.026	0.001	0.026	0.000	94.20	0.74
IPCWsL1*L2*	-0.074	0.001	0.006	0.001	0.025	0.001	0.026	0.001	0.026	0.000	94.50	0.72

Table C15. Table of simsum results for scenario 7

	Mean Estimate	MCSE	Bias	MCSE	EmpSE	MCSE	RMSE	MCSE	ModSE	MCSE	Coverage(%)	MCSE
ITT	-0.067	0.001	0.012	0.001	0.021	0.000	0.024	0.000	0.022	0.000	92.60	0.83
PP	-0.060	0.001	0.020	0.001	0.023	0.001	0.030	0.001	0.024	0.000	86.20	1.09
IPCWuL2	-0.072	0.001	0.007	0.001	0.027	0.001	0.028	0.001	0.027	0.000	93.20	0.80
IPCWsL2	-0.073	0.001	0.006	0.001	0.027	0.001	0.027	0.001	0.027	0.000	93.40	0.79
IPCWuL1	-0.073	0.001	0.006	0.001	0.027	0.001	0.028	0.001	0.028	0.000	93.80	0.76
IPCWsL1	-0.074	0.001	0.006	0.001	0.027	0.001	0.028	0.001	0.028	0.000	93.90	0.76
IPCWuL1L3	-0.073	0.001	0.006	0.001	0.027	0.001	0.028	0.001	0.028	0.001	93.70	0.77
IPCWsL1L3	-0.074	0.001	0.006	0.001	0.027	0.001	0.028	0.001	0.028	0.001	93.70	0.77
IPCWuL1L4	-0.073	0.001	0.006	0.001	0.027	0.001	0.028	0.001	0.028	0.000	93.60	0.77
IPCWsL1L4	-0.074	0.001	0.006	0.001	0.027	0.001	0.028	0.001	0.028	0.000	93.80	0.76
IPCWuL1L4L5	-0.073	0.001	0.007	0.001	0.027	0.001	0.028	0.001	0.027	0.000	93.80	0.76
IPCWsL1L4L5	-0.073	0.001	0.006	0.001	0.027	0.001	0.027	0.001	0.028	0.000	93.80	0.76
IPCWuL1L3L4L5	-0.073	0.001	0.007	0.001	0.027	0.001	0.028	0.001	0.028	0.000	93.80	0.76
IPCWsL1L3L4L5	-0.073	0.001	0.006	0.001	0.027	0.001	0.028	0.001	0.028	0.000	94.00	0.75
IPCWuLb1Lb2	-0.063	0.001	0.016	0.001	0.025	0.001	0.030	0.001	0.025	0.000	89.30	0.98
IPCWsLb1Lb2	-0.064	0.001	0.016	0.001	0.025	0.001	0.029	0.001	0.025	0.000	90.20	0.94
IPCWuL1*	-0.067	0.001	0.012	0.001	0.025	0.001	0.027	0.001	0.025	0.000	91.90	0.86
IPCWsL1*	-0.068	0.001	0.011	0.001	0.025	0.001	0.027	0.001	0.025	0.000	92.50	0.83
IPCWuL1L2	-0.080	0.001	-0.000	0.001	0.030	0.001	0.030	0.001	0.030	0.001	94.70	0.71
IPCWsL1L2	-0.080	0.001	-0.001	0.001	0.030	0.001	0.030	0.001	0.030	0.001	94.70	0.71
IPCWuL1L2L3	-0.079	0.001	-0.000	0.001	0.030	0.001	0.030	0.001	0.030	0.001	94.40	0.73
IPCWsL1L2L3	-0.080	0.001	-0.001	0.001	0.030	0.001	0.030	0.001	0.030	0.001	94.70	0.71
IPCWuL1L2L4	-0.079	0.001	-0.000	0.001	0.030	0.001	0.030	0.001	0.030	0.001	94.40	0.73
IPCWsL1L2L4	-0.080	0.001	-0.001	0.001	0.030	0.001	0.030	0.001	0.030	0.001	94.50	0.72
IPCWuL1L2L4L5	-0.079	0.001	0.000	0.001	0.029	0.001	0.029	0.001	0.029	0.001	94.70	0.71
IPCWsL1L2L4L5	-0.080	0.001	-0.000	0.001	0.029	0.001	0.029	0.001	0.029	0.001	94.90	0.70
IPCWuL1L2L3L4L5	-0.079	0.001	0.000	0.001	0.029	0.001	0.029	0.001	0.029	0.001	94.20	0.74
IPCWsL1L2L3L4L5	-0.080	0.001	-0.000	0.001	0.029	0.001	0.029	0.001	0.029	0.001	94.40	0.73
IPCWuL1*L2*	-0.071	0.001	0.008	0.001	0.025	0.001	0.026	0.001	0.026	0.000	93.30	0.79
IPCWsL1*L2*	-0.072	0.001	0.008	0.001	0.025	0.001	0.026	0.001	0.026	0.000	94.00	0.75

Table C16. Table of simsum results for scenario 8

	Mean Estimate	MCSE	Bias	MCSE	EmpSE	MCSE	RMSE	MCSE	ModSE	MCSE	Coverage(%)	MCSE
ITT	-0.070	0.001	0.009	0.001	0.021	0.000	0.023	0.000	0.022	0.000	95.10	0.68
PP	-0.076	0.001	0.003	0.001	0.023	0.001	0.024	0.000	0.024	0.000	95.90	0.63
IPCWuL2	-0.083	0.001	-0.004	0.001	0.025	0.001	0.026	0.001	0.026	0.000	95.40	0.66
IPCWsL2	-0.084	0.001	-0.005	0.001	0.025	0.001	0.026	0.001	0.026	0.000	95.10	0.68
IPCWuL1	-0.075	0.001	0.004	0.001	0.024	0.001	0.024	0.001	0.025	0.000	95.20	0.68
IPCWsL1	-0.076	0.001	0.003	0.001	0.024	0.001	0.024	0.001	0.025	0.000	95.30	0.67
IPCWuL1L3	-0.076	0.001	0.004	0.001	0.024	0.001	0.024	0.001	0.025	0.000	95.20	0.68
IPCWsL1L3	-0.077	0.001	0.003	0.001	0.024	0.001	0.024	0.001	0.025	0.000	95.30	0.67
IPCWuL1L4	-0.075	0.001	0.004	0.001	0.024	0.001	0.024	0.001	0.025	0.000	95.10	0.68
IPCWsL1L4	-0.076	0.001	0.003	0.001	0.024	0.001	0.024	0.001	0.025	0.000	95.30	0.67
IPCWuL1L4L5	-0.075	0.001	0.004	0.001	0.024	0.001	0.024	0.001	0.025	0.000	95.20	0.68
IPCWsL1L4L5	-0.076	0.001	0.003	0.001	0.024	0.001	0.024	0.001	0.025	0.000	95.30	0.67
IPCWuL1L3L4L5	-0.075	0.001	0.004	0.001	0.024	0.001	0.024	0.001	0.025	0.000	95.20	0.68
IPCWsL1L3L4L5	-0.076	0.001	0.003	0.001	0.024	0.001	0.024	0.001	0.025	0.000	95.40	0.66
IPCWuLb1Lb2	-0.079	0.001	0.001	0.001	0.024	0.001	0.024	0.001	0.025	0.000	95.80	0.63
IPCWsLb1Lb2	-0.080	0.001	-0.000	0.001	0.025	0.001	0.024	0.001	0.025	0.000	95.90	0.63
IPCWuL1*	-0.073	0.001	0.006	0.001	0.024	0.001	0.025	0.001	0.025	0.000	94.10	0.75
IPCWsL1*	-0.074	0.001	0.005	0.001	0.024	0.001	0.025	0.001	0.025	0.000	94.40	0.73
IPCWuL1L2	-0.080	0.001	-0.000	0.001	0.025	0.001	0.025	0.001	0.026	0.000	95.50	0.66
IPCWsL1L2	-0.081	0.001	-0.001	0.001	0.025	0.001	0.025	0.001	0.026	0.000	95.50	0.66
IPCWuL1L2L3	-0.080	0.001	-0.000	0.001	0.025	0.001	0.025	0.001	0.026	0.000	95.30	0.67
IPCWsL1L2L3	-0.081	0.001	-0.001	0.001	0.025	0.001	0.025	0.001	0.026	0.000	95.30	0.67
IPCWuL1L2L4	-0.080	0.001	-0.000	0.001	0.025	0.001	0.025	0.001	0.026	0.000	95.50	0.66
IPCWsL1L2L4	-0.081	0.001	-0.001	0.001	0.025	0.001	0.025	0.001	0.026	0.000	95.70	0.64
IPCWuL1L2L4L5	-0.080	0.001	-0.000	0.001	0.025	0.001	0.025	0.001	0.026	0.000	95.50	0.66
IPCWsL1L2L4L5	-0.081	0.001	-0.001	0.001	0.025	0.001	0.025	0.001	0.026	0.000	95.40	0.66
IPCWuL1L2L3L4L5	-0.080	0.001	-0.000	0.001	0.025	0.001	0.025	0.001	0.026	0.000	95.40	0.66
IPCWsL1L2L3L4L5	-0.081	0.001	-0.001	0.001	0.025	0.001	0.025	0.001	0.026	0.000	95.40	0.66
IPCWuL1*L2*	-0.076	0.001	0.003	0.001	0.024	0.001	0.024	0.001	0.025	0.000	94.90	0.70
IPCWsL1*L2*	-0.077	0.001	0.002	0.001	0.024	0.001	0.024	0.001	0.025	0.000	95.10	0.68

Table C17. Table of simsum results for scenario 9

	Mean Estimate	MCSE	Bias	MCSE	EmpSE	MCSE	RMSE	MCSE	ModSE	MCSE	Coverage(%)	MCSE
ITT	-0.062	0.001	0.017	0.001	0.021	0.000	0.027	0.001	0.022	0.000	88.60	1.01
PP	-0.039	0.001	0.040	0.001	0.023	0.001	0.046	0.001	0.024	0.000	57.40	1.56
IPCWuL2	-0.060	0.001	0.019	0.001	0.033	0.001	0.038	0.001	0.033	0.001	84.05	1.16
IPCWsL2	-0.061	0.001	0.019	0.001	0.033	0.001	0.038	0.001	0.033	0.001	84.45	1.15
IPCWuL1	-0.060	0.001	0.020	0.001	0.034	0.001	0.039	0.001	0.034	0.001	83.30	1.18
IPCWsL1	-0.060	0.001	0.019	0.001	0.034	0.001	0.039	0.001	0.034	0.001	83.80	1.17
IPCWuL1L3	-0.060	0.001	0.019	0.001	0.034	0.001	0.039	0.001	0.034	0.001	84.30	1.15
IPCWsL1L3	-0.061	0.001	0.019	0.001	0.034	0.001	0.039	0.001	0.034	0.001	84.50	1.14
IPCWuL1L4	-0.059	0.001	0.020	0.001	0.033	0.001	0.039	0.001	0.033	0.001	83.00	1.19
IPCWsL1L4	-0.059	0.001	0.020	0.001	0.033	0.001	0.039	0.001	0.033	0.001	83.30	1.18
IPCWuL1L4L5	-0.059	0.001	0.021	0.001	0.033	0.001	0.039	0.001	0.033	0.001	82.40	1.20
IPCWsL1L4L5	-0.059	0.001	0.020	0.001	0.033	0.001	0.038	0.001	0.033	0.001	83.10	1.19
IPCWuL1L3L4L5	-0.060	0.001	0.020	0.001	0.033	0.001	0.038	0.001	0.033	0.001	83.20	1.18
IPCWsL1L3L4L5	-0.060	0.001	0.020	0.001	0.033	0.001	0.038	0.001	0.033	0.001	83.80	1.17
IPCWuLb1Lb2	-0.046	0.001	0.033	0.001	0.026	0.001	0.042	0.001	0.026	0.001	69.80	1.45
IPCWsLb1Lb2	-0.046	0.001	0.033	0.001	0.026	0.001	0.042	0.001	0.026	0.001	70.20	1.45
IPCWuL1*	-0.052	0.001	0.027	0.001	0.026	0.001	0.037	0.001	0.026	0.000	78.40	1.30
IPCWsL1*	-0.053	0.001	0.027	0.001	0.026	0.001	0.037	0.001	0.027	0.000	79.20	1.28
IPCWuL1L2	-0.064	0.001	0.016	0.001	0.030	0.001	0.034	0.001	0.031	0.001	88.19	1.02
IPCWsL1L2	-0.064	0.001	0.015	0.001	0.030	0.001	0.034	0.001	0.031	0.001	89.09	0.99
IPCWuL1L2L3	-0.064	0.001	0.015	0.001	0.030	0.001	0.034	0.001	0.031	0.001	88.99	0.99
IPCWsL1L2L3	-0.065	0.001	0.015	0.001	0.030	0.001	0.034	0.001	0.031	0.001	89.49	0.97
IPCWuL1L2L4	-0.063	0.001	0.016	0.001	0.030	0.001	0.034	0.001	0.031	0.001	88.39	1.01
IPCWsL1L2L4	-0.064	0.001	0.015	0.001	0.030	0.001	0.034	0.001	0.031	0.001	88.69	1.00
IPCWuL1L2L4L5	-0.064	0.001	0.016	0.001	0.031	0.001	0.035	0.001	0.032	0.001	87.99	1.03
IPCWsL1L2L4L5	-0.064	0.001	0.015	0.001	0.031	0.001	0.035	0.001	0.032	0.001	88.29	1.02
IPCWuL1L2L3L4L5	-0.064	0.001	0.015	0.001	0.031	0.001	0.035	0.001	0.032	0.001	88.39	1.01
IPCWsL1L2L3L4L5	-0.065	0.001	0.015	0.001	0.031	0.001	0.034	0.001	0.032	0.001	88.49	1.01
IPCWuL1*L2*	-0.057	0.001	0.022	0.001	0.026	0.001	0.034	0.001	0.027	0.000	83.80	1.17
IPCWsL1*L2*	-0.058	0.001	0.022	0.001	0.026	0.001	0.034	0.001	0.027	0.000	84.40	1.15

Table C18. Table of simsum results for scenario 10

	Mean Estimate	MCSE	Bias	MCSE	EmpSE	MCSE	RMSE	MCSE	ModSE	MCSE	Coverage(%)	MCSE
ITT	-0.072	0.001	0.007	0.001	0.021	0.000	0.022	0.000	0.022	0.000	95.70	0.64
PP	-0.071	0.001	0.008	0.001	0.023	0.001	0.025	0.001	0.024	0.000	93.40	0.79
IPCWuL2	-0.077	0.001	0.002	0.001	0.029	0.001	0.029	0.001	0.029	0.000	95.10	0.68
IPCWsL2	-0.078	0.001	0.001	0.001	0.029	0.001	0.029	0.001	0.029	0.000	95.20	0.68
IPCWuL1	-0.077	0.001	0.003	0.001	0.028	0.001	0.028	0.001	0.029	0.000	95.20	0.68
IPCWsL1	-0.077	0.001	0.002	0.001	0.028	0.001	0.028	0.001	0.029	0.000	95.20	0.68
IPCWuL1L3	-0.077	0.001	0.003	0.001	0.028	0.001	0.028	0.001	0.029	0.000	95.40	0.66
IPCWsL1L3	-0.077	0.001	0.002	0.001	0.028	0.001	0.028	0.001	0.029	0.000	95.50	0.66
IPCWuL1L4	-0.076	0.001	0.003	0.001	0.028	0.001	0.028	0.001	0.029	0.000	95.30	0.67
IPCWsL1L4	-0.077	0.001	0.002	0.001	0.028	0.001	0.028	0.001	0.029	0.000	95.40	0.66
IPCWuL1L4L5	-0.076	0.001	0.003	0.001	0.028	0.001	0.028	0.001	0.028	0.000	94.90	0.70
IPCWsL1L4L5	-0.077	0.001	0.003	0.001	0.028	0.001	0.028	0.001	0.029	0.000	95.30	0.67
IPCWuL1L3L4L5	-0.076	0.001	0.003	0.001	0.028	0.001	0.028	0.001	0.029	0.000	95.10	0.68
IPCWsL1L3L4L5	-0.077	0.001	0.003	0.001	0.028	0.001	0.028	0.001	0.029	0.000	95.20	0.68
IPCWuLb1Lb2	-0.072	0.001	0.007	0.001	0.026	0.001	0.027	0.001	0.026	0.000	94.90	0.70
IPCWsLb1Lb2	-0.073	0.001	0.006	0.001	0.026	0.001	0.026	0.001	0.026	0.000	94.90	0.70
IPCWuL1*	-0.074	0.001	0.005	0.001	0.026	0.001	0.026	0.001	0.026	0.000	94.90	0.70
IPCWsL1*	-0.075	0.001	0.005	0.001	0.026	0.001	0.026	0.001	0.027	0.000	95.00	0.69
IPCWuL1L2	-0.080	0.001	-0.000	0.001	0.030	0.001	0.030	0.001	0.031	0.001	96.00	0.62
IPCWsL1L2	-0.081	0.001	-0.001	0.001	0.030	0.001	0.030	0.001	0.031	0.001	96.10	0.61
IPCWuL1L2L3	-0.080	0.001	-0.000	0.001	0.030	0.001	0.030	0.001	0.031	0.000	95.70	0.64
IPCWsL1L2L3	-0.081	0.001	-0.001	0.001	0.030	0.001	0.030	0.001	0.031	0.000	95.80	0.63
IPCWuL1L2L4	-0.080	0.001	-0.000	0.001	0.030	0.001	0.030	0.001	0.031	0.000	95.60	0.65
IPCWsL1L2L4	-0.080	0.001	-0.001	0.001	0.031	0.001	0.031	0.001	0.031	0.000	96.10	0.61
IPCWuL1L2L4L5	-0.079	0.001	0.000	0.001	0.030	0.001	0.030	0.001	0.030	0.000	95.30	0.67
IPCWsL1L2L4L5	-0.080	0.001	-0.001	0.001	0.030	0.001	0.030	0.001	0.031	0.000	95.70	0.64
IPCWuL1L2L3L4L5	-0.079	0.001	0.000	0.001	0.030	0.001	0.030	0.001	0.030	0.000	95.30	0.67
IPCWsL1L2L3L4L5	-0.080	0.001	-0.001	0.001	0.030	0.001	0.030	0.001	0.031	0.000	95.40	0.66
IPCWuL1*L2*	-0.076	0.001	0.003	0.001	0.026	0.001	0.027	0.001	0.027	0.000	95.10	0.68
IPCWsL1*L2*	-0.077	0.001	0.003	0.001	0.027	0.001	0.027	0.001	0.027	0.000	95.00	0.69

Table C19. Table of simsum results for scenario 11

	Mean Estimate	MCSE	Bias	MCSE	EmpSE	MCSE	RMSE	MCSE	ModSE	MCSE	Coverage(%)	MCSE
ITT	-0.071	0.001	0.009	0.001	0.021	0.000	0.023	0.000	0.022	0.000	94.90	0.70
PP	-0.068	0.001	0.011	0.001	0.023	0.001	0.026	0.001	0.024	0.000	91.90	0.86
IPCWuL2	-0.077	0.001	0.003	0.001	0.028	0.001	0.028	0.001	0.028	0.000	93.90	0.76
IPCWsL2	-0.077	0.001	0.002	0.001	0.028	0.001	0.028	0.001	0.028	0.000	94.00	0.75
IPCWuL1	-0.076	0.001	0.004	0.001	0.028	0.001	0.028	0.001	0.028	0.000	94.40	0.73
IPCWsL1	-0.076	0.001	0.003	0.001	0.028	0.001	0.028	0.001	0.028	0.000	94.30	0.73
IPCWuL1L3	-0.076	0.001	0.004	0.001	0.028	0.001	0.028	0.001	0.028	0.000	94.10	0.75
IPCWsL1L3	-0.076	0.001	0.003	0.001	0.028	0.001	0.028	0.001	0.028	0.000	94.20	0.74
IPCWuL1L4	-0.075	0.001	0.004	0.001	0.028	0.001	0.028	0.001	0.028	0.000	94.40	0.73
IPCWsL1L4	-0.076	0.001	0.003	0.001	0.028	0.001	0.028	0.001	0.028	0.000	94.50	0.72
IPCWuL1L4L5	-0.075	0.001	0.004	0.001	0.028	0.001	0.028	0.001	0.028	0.000	94.20	0.74
IPCWsL1L4L5	-0.076	0.001	0.004	0.001	0.028	0.001	0.028	0.001	0.028	0.000	94.50	0.72
IPCWuL1L3L4L5	-0.075	0.001	0.004	0.001	0.028	0.001	0.028	0.001	0.028	0.000	94.10	0.75
IPCWsL1L3L4L5	-0.076	0.001	0.003	0.001	0.028	0.001	0.028	0.001	0.028	0.000	94.40	0.73
IPCWuLb1Lb2	-0.070	0.001	0.009	0.001	0.025	0.001	0.027	0.001	0.026	0.000	93.20	0.80
IPCWsLb1Lb2	-0.071	0.001	0.008	0.001	0.026	0.001	0.027	0.001	0.026	0.000	93.70	0.77
IPCWuL1*	-0.072	0.001	0.007	0.001	0.026	0.001	0.027	0.001	0.026	0.000	94.00	0.75
IPCWsL1*	-0.073	0.001	0.006	0.001	0.026	0.001	0.026	0.001	0.026	0.000	94.00	0.75
IPCWuL1L2	-0.080	0.001	-0.001	0.001	0.030	0.001	0.030	0.001	0.030	0.001	94.40	0.73
IPCWsL1L2	-0.081	0.001	-0.001	0.001	0.030	0.001	0.030	0.001	0.030	0.001	94.50	0.72
IPCWuL1L2L3	-0.080	0.001	-0.001	0.001	0.030	0.001	0.030	0.001	0.030	0.000	94.50	0.72
IPCWsL1L2L3	-0.081	0.001	-0.001	0.001	0.030	0.001	0.030	0.001	0.030	0.000	94.60	0.71
IPCWuL1L2L4	-0.080	0.001	-0.001	0.001	0.030	0.001	0.030	0.001	0.030	0.000	94.30	0.73
IPCWsL1L2L4	-0.081	0.001	-0.001	0.001	0.030	0.001	0.030	0.001	0.030	0.000	94.50	0.72
IPCWuL1L2L4L5	-0.079	0.001	0.000	0.001	0.029	0.001	0.029	0.001	0.030	0.000	94.10	0.75
IPCWsL1L2L4L5	-0.080	0.001	-0.001	0.001	0.030	0.001	0.030	0.001	0.030	0.000	94.30	0.73
IPCWuL1L2L3L4L5	-0.079	0.001	0.000	0.001	0.029	0.001	0.029	0.001	0.030	0.000	94.20	0.74
IPCWsL1L2L3L4L5	-0.080	0.001	-0.001	0.001	0.030	0.001	0.030	0.001	0.030	0.000	94.40	0.73
IPCWuL1*L2*	-0.075	0.001	0.005	0.001	0.026	0.001	0.027	0.001	0.027	0.000	94.50	0.72
IPCWsL1*L2*	-0.075	0.001	0.004	0.001	0.026	0.001	0.027	0.001	0.027	0.000	94.40	0.73

Table C20. Table of simsum results for scenario 12

	Mean Estimate	MCSE	Bias	MCSE	EmpSE	MCSE	RMSE	MCSE	ModSE	MCSE	Coverage(%)	MCSE
ITT	-0.074	0.001	0.005	0.001	0.021	0.000	0.022	0.000	0.022	0.000	96.60	0.57
PP	-0.074	0.001	0.005	0.001	0.024	0.001	0.024	0.000	0.024	0.000	94.90	0.70
IPCWuL2	-0.078	0.001	0.001	0.001	0.031	0.001	0.031	0.001	0.031	0.000	95.90	0.63
IPCWsL2	-0.079	0.001	0.001	0.001	0.031	0.001	0.031	0.001	0.031	0.000	95.90	0.63
IPCWuL1	-0.078	0.001	0.002	0.001	0.029	0.001	0.029	0.001	0.030	0.000	96.00	0.62
IPCWsL1	-0.078	0.001	0.001	0.001	0.029	0.001	0.029	0.001	0.030	0.000	96.00	0.62
IPCWuL1L3	-0.078	0.001	0.002	0.001	0.029	0.001	0.029	0.001	0.030	0.000	95.90	0.63
IPCWsL1L3	-0.078	0.001	0.001	0.001	0.029	0.001	0.029	0.001	0.030	0.000	96.00	0.62
IPCWuL1L4	-0.078	0.001	0.002	0.001	0.030	0.001	0.030	0.001	0.030	0.000	96.10	0.61
IPCWsL1L4	-0.078	0.001	0.001	0.001	0.030	0.001	0.030	0.001	0.030	0.000	96.10	0.61
IPCWuL1L4L5	-0.077	0.001	0.002	0.001	0.030	0.001	0.030	0.001	0.030	0.000	95.50	0.66
IPCWsL1L4L5	-0.078	0.001	0.002	0.001	0.030	0.001	0.030	0.001	0.030	0.000	95.80	0.63
IPCWuL1L3L4L5	-0.077	0.001	0.002	0.001	0.030	0.001	0.030	0.001	0.030	0.000	95.70	0.64
IPCWsL1L3L4L5	-0.078	0.001	0.001	0.001	0.030	0.001	0.030	0.001	0.030	0.000	95.70	0.64
IPCWuLb1Lb2	-0.075	0.001	0.004	0.001	0.028	0.001	0.028	0.002	0.028	0.001	95.50	0.66
IPCWsLb1Lb2	-0.075	0.001	0.004	0.001	0.028	0.001	0.028	0.002	0.028	0.001	95.70	0.64
IPCWuL1*	-0.076	0.001	0.003	0.001	0.027	0.001	0.027	0.001	0.027	0.000	95.40	0.66
IPCWsL1*	-0.077	0.001	0.002	0.001	0.027	0.001	0.027	0.001	0.027	0.000	95.40	0.66
IPCWuL1L2	-0.080	0.001	-0.000	0.001	0.032	0.001	0.032	0.001	0.032	0.001	95.60	0.65
IPCWsL1L2	-0.080	0.001	-0.001	0.001	0.032	0.001	0.032	0.001	0.032	0.001	95.80	0.63
IPCWuL1L2L3	-0.080	0.001	-0.000	0.001	0.032	0.001	0.032	0.001	0.032	0.001	95.40	0.66
IPCWsL1L2L3	-0.080	0.001	-0.001	0.001	0.032	0.001	0.032	0.001	0.032	0.001	95.50	0.66
IPCWuL1L2L4	-0.080	0.001	-0.000	0.001	0.032	0.001	0.032	0.001	0.033	0.001	95.90	0.63
IPCWsL1L2L4	-0.080	0.001	-0.001	0.001	0.032	0.001	0.032	0.001	0.033	0.001	96.20	0.60
IPCWuL1L2L4L5	-0.079	0.001	0.000	0.001	0.032	0.001	0.032	0.001	0.032	0.000	96.20	0.61
IPCWsL1L2L4L5	-0.080	0.001	-0.001	0.001	0.032	0.001	0.032	0.001	0.032	0.000	96.30	0.60
IPCWuL1L2L3L4L5	-0.079	0.001	0.000	0.001	0.032	0.001	0.032	0.001	0.032	0.000	95.90	0.63
IPCWsL1L2L3L4L5	-0.080	0.001	-0.001	0.001	0.032	0.001	0.032	0.001	0.032	0.000	95.80	0.63
IPCWuL1*L2*	-0.078	0.001	0.002	0.001	0.028	0.001	0.028	0.001	0.028	0.000	95.50	0.66
IPCWsL1*L2*	-0.078	0.001	0.001	0.001	0.028	0.001	0.028	0.001	0.028	0.000	95.70	0.64

Table C21. Table of simsum results for scenario 13

	Mean Estimate	MCSE	Bias	MCSE	EmpSE	MCSE	RMSE	MCSE	ModSE	MCSE	Coverage(%)	MCSE
ITT	-0.044	0.001	0.012	0.001	0.022	0.001	0.026	0.001	0.023	0.000	91.40	0.89
PP	-0.038	0.001	0.018	0.001	0.024	0.001	0.030	0.001	0.024	0.000	87.80	1.03
IPCWuL2	-0.051	0.001	0.005	0.001	0.028	0.001	0.029	0.001	0.028	0.000	93.30	0.79
IPCWsL2	-0.052	0.001	0.004	0.001	0.028	0.001	0.029	0.001	0.028	0.000	93.40	0.79
IPCWuL1	-0.050	0.001	0.006	0.001	0.028	0.001	0.029	0.001	0.028	0.000	92.80	0.82
IPCWsL1	-0.051	0.001	0.006	0.001	0.028	0.001	0.029	0.001	0.028	0.000	93.20	0.80
IPCWuL1L3	-0.050	0.001	0.006	0.001	0.028	0.001	0.029	0.001	0.028	0.000	93.10	0.80
IPCWsL1L3	-0.051	0.001	0.006	0.001	0.028	0.001	0.029	0.001	0.028	0.000	93.30	0.79
IPCWuL1L4	-0.049	0.001	0.007	0.001	0.028	0.001	0.028	0.001	0.028	0.000	92.60	0.83
IPCWsL1L4	-0.050	0.001	0.006	0.001	0.028	0.001	0.028	0.001	0.028	0.000	92.70	0.82
IPCWuL1L4L5	-0.049	0.001	0.007	0.001	0.028	0.001	0.029	0.001	0.028	0.000	92.80	0.82
IPCWsL1L4L5	-0.050	0.001	0.006	0.001	0.028	0.001	0.028	0.001	0.028	0.000	92.80	0.82
IPCWuL1L3L4L5	-0.049	0.001	0.007	0.001	0.028	0.001	0.029	0.001	0.028	0.000	93.00	0.81
IPCWsL1L3L4L5	-0.050	0.001	0.006	0.001	0.028	0.001	0.029	0.001	0.028	0.000	93.10	0.80
IPCWuLb1Lb2	-0.042	0.001	0.015	0.001	0.026	0.001	0.030	0.001	0.026	0.000	90.60	0.92
IPCWsLb1Lb2	-0.042	0.001	0.014	0.001	0.026	0.001	0.029	0.001	0.026	0.000	90.90	0.91
IPCWuL1*	-0.044	0.001	0.012	0.001	0.026	0.001	0.029	0.001	0.026	0.000	91.99	0.86
IPCWsL1*	-0.045	0.001	0.011	0.001	0.026	0.001	0.028	0.001	0.026	0.000	92.00	0.86
IPCWuL1L2	-0.056	0.001	-0.000	0.001	0.030	0.001	0.030	0.001	0.030	0.001	93.90	0.76
IPCWsL1L2	-0.057	0.001	-0.001	0.001	0.030	0.001	0.030	0.001	0.030	0.001	93.99	0.75
IPCWuL1L2L3	-0.056	0.001	-0.000	0.001	0.030	0.001	0.030	0.001	0.030	0.000	93.89	0.76
IPCWsL1L2L3	-0.057	0.001	-0.001	0.001	0.030	0.001	0.030	0.001	0.030	0.000	93.99	0.75
IPCWuL1L2L4	-0.056	0.001	0.000	0.001	0.030	0.001	0.030	0.001	0.030	0.000	93.70	0.77
IPCWsL1L2L4	-0.057	0.001	-0.001	0.001	0.030	0.001	0.030	0.001	0.030	0.000	93.90	0.76
IPCWuL1L2L4L5	-0.055	0.001	0.001	0.001	0.029	0.001	0.029	0.001	0.029	0.000	93.40	0.79
IPCWsL1L2L4L5	-0.056	0.001	-0.000	0.001	0.029	0.001	0.029	0.001	0.029	0.000	93.80	0.76
IPCWuL1L2L3L4L5	-0.055	0.001	0.001	0.001	0.029	0.001	0.029	0.001	0.029	0.000	93.70	0.77
IPCWsL1L2L3L4L5	-0.056	0.001	-0.000	0.001	0.029	0.001	0.029	0.001	0.029	0.000	93.70	0.77
IPCWuL1*L2*	-0.048	0.001	0.008	0.001	0.027	0.001	0.028	0.001	0.027	0.000	92.70	0.82
IPCWsL1*L2*	-0.049	0.001	0.007	0.001	0.027	0.001	0.028	0.001	0.027	0.000	92.90	0.81

Table C22. Table of simsum results for scenario 14

	Mean Estimate	MCSE	Bias	MCSE	EmpSE	MCSE	RMSE	MCSE	ModSE	MCSE	Coverage(%)	MCSE
ITT	-0.093	0.001	0.012	0.001	0.021	0.000	0.024	0.000	0.021	0.000	92.00	0.86
PP	-0.087	0.001	0.017	0.001	0.022	0.001	0.028	0.001	0.023	0.000	88.00	1.03
IPCWuL2	-0.100	0.001	0.005	0.001	0.027	0.001	0.027	0.001	0.027	0.000	93.70	0.77
IPCWsL2	-0.101	0.001	0.004	0.001	0.027	0.001	0.027	0.001	0.027	0.000	93.80	0.76
IPCWuL1	-0.099	0.001	0.006	0.001	0.026	0.001	0.027	0.001	0.027	0.000	93.20	0.80
IPCWsL1	-0.099	0.001	0.005	0.001	0.027	0.001	0.027	0.001	0.027	0.000	93.80	0.76
IPCWuL1L3	-0.099	0.001	0.006	0.001	0.027	0.001	0.027	0.001	0.027	0.000	93.50	0.78
IPCWsL1L3	-0.099	0.001	0.005	0.001	0.027	0.001	0.027	0.001	0.027	0.000	93.80	0.76
IPCWuL1L4	-0.098	0.001	0.006	0.001	0.026	0.001	0.027	0.001	0.026	0.000	93.10	0.80
IPCWsL1L4	-0.099	0.001	0.005	0.001	0.026	0.001	0.027	0.001	0.027	0.000	93.40	0.79
IPCWuL1L4L5	-0.098	0.001	0.007	0.001	0.026	0.001	0.027	0.001	0.026	0.000	92.90	0.81
IPCWsL1L4L5	-0.099	0.001	0.006	0.001	0.026	0.001	0.027	0.001	0.026	0.000	93.80	0.76
IPCWuL1L3L4L5	-0.098	0.001	0.006	0.001	0.026	0.001	0.027	0.001	0.026	0.000	93.10	0.80
IPCWsL1L3L4L5	-0.099	0.001	0.006	0.001	0.026	0.001	0.027	0.001	0.026	0.000	93.90	0.76
IPCWuLb1Lb2	-0.090	0.001	0.014	0.001	0.024	0.001	0.028	0.001	0.025	0.000	89.90	0.95
IPCWsLb1Lb2	-0.091	0.001	0.013	0.001	0.024	0.001	0.028	0.001	0.025	0.000	90.60	0.92
IPCWuL1*	-0.093	0.001	0.011	0.001	0.024	0.001	0.027	0.001	0.025	0.000	91.70	0.87
IPCWsL1*	-0.094	0.001	0.010	0.001	0.025	0.001	0.027	0.001	0.025	0.000	92.00	0.86
IPCWuL1L2	-0.105	0.001	-0.001	0.001	0.028	0.001	0.028	0.001	0.028	0.001	95.00	0.69
IPCWsL1L2	-0.106	0.001	-0.002	0.001	0.028	0.001	0.029	0.001	0.029	0.001	94.80	0.70
IPCWuL1L2L3	-0.105	0.001	-0.001	0.001	0.028	0.001	0.028	0.001	0.028	0.001	94.90	0.70
IPCWsL1L2L3	-0.106	0.001	-0.001	0.001	0.028	0.001	0.028	0.001	0.028	0.001	94.80	0.70
IPCWuL1L2L4	-0.105	0.001	-0.000	0.001	0.029	0.001	0.029	0.001	0.029	0.000	94.40	0.73
IPCWsL1L2L4	-0.106	0.001	-0.001	0.001	0.029	0.001	0.029	0.001	0.029	0.001	94.70	0.71
IPCWuL1L2L4L5	-0.104	0.001	0.000	0.001	0.028	0.001	0.028	0.001	0.028	0.000	93.90	0.76
IPCWsL1L2L4L5	-0.105	0.001	-0.001	0.001	0.028	0.001	0.028	0.001	0.028	0.000	94.00	0.75
IPCWuL1L2L3L4L5	-0.104	0.001	0.000	0.001	0.028	0.001	0.028	0.001	0.028	0.000	93.90	0.76
IPCWsL1L2L3L4L5	-0.105	0.001	-0.001	0.001	0.028	0.001	0.028	0.001	0.028	0.000	93.90	0.76
IPCWuL1*L2*	-0.097	0.001	0.007	0.001	0.025	0.001	0.026	0.001	0.025	0.000	93.10	0.80
IPCWsL1*L2*	-0.098	0.001	0.007	0.001	0.025	0.001	0.026	0.001	0.025	0.000	93.80	0.76

Table C23. Table of simsum results for scenario 15

	Mean Estimate	MCSE	Bias	MCSE	EmpSE	MCSE	RMSE	MCSE	ModSE	MCSE	Coverage(%)	MCSE
ITT	-0.074	0.001	0.013	0.001	0.023	0.001	0.026	0.001	0.024	0.000	93.50	0.78
PP	-0.070	0.001	0.017	0.001	0.025	0.001	0.030	0.001	0.026	0.000	90.20	0.94
IPCWuL2	-0.084	0.001	0.003	0.001	0.030	0.001	0.030	0.001	0.030	0.000	94.70	0.71
IPCWsL2	-0.085	0.001	0.002	0.001	0.030	0.001	0.030	0.001	0.030	0.000	94.80	0.70
IPCWuL1	-0.080	0.001	0.007	0.001	0.029	0.001	0.029	0.001	0.029	0.000	93.70	0.77
IPCWsL1	-0.081	0.001	0.006	0.001	0.029	0.001	0.029	0.001	0.029	0.000	94.30	0.73
IPCWuL1L3	-0.080	0.001	0.007	0.001	0.029	0.001	0.029	0.001	0.029	0.000	94.00	0.75
IPCWsL1L3	-0.081	0.001	0.006	0.001	0.029	0.001	0.029	0.001	0.029	0.000	94.40	0.73
IPCWuL1L4	-0.080	0.001	0.007	0.001	0.029	0.001	0.029	0.001	0.029	0.000	93.80	0.76
IPCWsL1L4	-0.081	0.001	0.006	0.001	0.029	0.001	0.029	0.001	0.029	0.000	94.10	0.75
IPCWuL1L4L5	-0.079	0.001	0.007	0.001	0.028	0.001	0.029	0.001	0.029	0.000	93.50	0.78
IPCWsL1L4L5	-0.081	0.001	0.006	0.001	0.029	0.001	0.029	0.001	0.029	0.000	94.10	0.75
IPCWuL1L3L4L5	-0.080	0.001	0.007	0.001	0.029	0.001	0.029	0.001	0.029	0.000	93.90	0.76
IPCWsL1L3L4L5	-0.081	0.001	0.006	0.001	0.029	0.001	0.029	0.001	0.029	0.000	94.20	0.74
IPCWuLb1Lb2	-0.073	0.001	0.013	0.001	0.027	0.001	0.030	0.001	0.028	0.000	91.69	0.87
IPCWsLb1Lb2	-0.074	0.001	0.012	0.001	0.027	0.001	0.030	0.001	0.028	0.000	91.79	0.87
IPCWuL1*	-0.076	0.001	0.011	0.001	0.027	0.001	0.029	0.001	0.028	0.000	93.10	0.80
IPCWsL1*	-0.077	0.001	0.010	0.001	0.027	0.001	0.029	0.001	0.028	0.000	93.40	0.79
IPCWuL1L2	-0.088	0.001	-0.001	0.001	0.031	0.001	0.031	0.001	0.031	0.000	95.10	0.68
IPCWsL1L2	-0.089	0.001	-0.002	0.001	0.031	0.001	0.031	0.001	0.031	0.000	95.10	0.68
IPCWuL1L2L3	-0.087	0.001	-0.001	0.001	0.031	0.001	0.031	0.001	0.031	0.000	95.30	0.67
IPCWsL1L2L3	-0.089	0.001	-0.002	0.001	0.031	0.001	0.031	0.001	0.031	0.000	95.50	0.66
IPCWuL1L2L4	-0.087	0.001	-0.001	0.001	0.031	0.001	0.031	0.001	0.031	0.000	94.90	0.70
IPCWsL1L2L4	-0.088	0.001	-0.002	0.001	0.031	0.001	0.031	0.001	0.031	0.000	95.10	0.68
IPCWuL1L2L4L5	-0.087	0.001	-0.000	0.001	0.030	0.001	0.030	0.001	0.031	0.000	94.80	0.70
IPCWsL1L2L4L5	-0.088	0.001	-0.001	0.001	0.030	0.001	0.030	0.001	0.031	0.000	95.10	0.68
IPCWuL1L2L3L4L5	-0.087	0.001	-0.000	0.001	0.030	0.001	0.030	0.001	0.031	0.000	95.20	0.68
IPCWsL1L2L3L4L5	-0.088	0.001	-0.001	0.001	0.030	0.001	0.030	0.001	0.031	0.000	95.10	0.68
IPCWuL1*L2*	-0.080	0.001	0.006	0.001	0.028	0.001	0.029	0.001	0.028	0.000	94.30	0.73
IPCWsL1*L2*	-0.082	0.001	0.005	0.001	0.028	0.001	0.028	0.001	0.029	0.000	94.70	0.71

Table C24. Table of simsum results for scenario 16

	Mean Estimate	MCSE	Bias	MCSE	EmpSE	MCSE	RMSE	MCSE	ModSE	MCSE	Coverage(%)	MCSE
ITT	-0.062	0.001	0.012	0.001	0.020	0.000	0.023	0.000	0.021	0.000	93.50	0.78
PP	-0.056	0.001	0.019	0.001	0.021	0.000	0.028	0.001	0.022	0.000	86.70	1.07
IPCWuL2	-0.068	0.001	0.007	0.001	0.025	0.001	0.026	0.001	0.026	0.000	93.10	0.80
IPCWsL2	-0.068	0.001	0.006	0.001	0.025	0.001	0.026	0.001	0.026	0.000	93.20	0.80
IPCWuL1	-0.068	0.001	0.006	0.001	0.025	0.001	0.026	0.001	0.026	0.000	94.00	0.75
IPCWsL1	-0.069	0.001	0.006	0.001	0.025	0.001	0.026	0.001	0.026	0.000	94.20	0.74
IPCWuL1L3	-0.068	0.001	0.006	0.001	0.025	0.001	0.026	0.001	0.026	0.000	94.10	0.75
IPCWsL1L3	-0.069	0.001	0.006	0.001	0.025	0.001	0.026	0.001	0.026	0.000	94.40	0.73
IPCWuL1L4	-0.068	0.001	0.006	0.001	0.025	0.001	0.026	0.001	0.026	0.000	93.60	0.77
IPCWsL1L4	-0.069	0.001	0.006	0.001	0.025	0.001	0.026	0.001	0.026	0.000	94.00	0.75
IPCWuL1L4L5	-0.068	0.001	0.007	0.001	0.025	0.001	0.026	0.001	0.026	0.000	93.90	0.76
IPCWsL1L4L5	-0.068	0.001	0.006	0.001	0.025	0.001	0.026	0.001	0.026	0.000	94.20	0.74
IPCWuL1L3L4L5	-0.068	0.001	0.007	0.001	0.025	0.001	0.026	0.001	0.026	0.000	93.90	0.76
IPCWsL1L3L4L5	-0.068	0.001	0.006	0.001	0.025	0.001	0.026	0.001	0.026	0.000	94.00	0.75
IPCWuLb1Lb2	-0.059	0.001	0.016	0.001	0.023	0.001	0.027	0.001	0.024	0.000	89.00	0.99
IPCWsLb1Lb2	-0.059	0.001	0.015	0.001	0.023	0.001	0.027	0.001	0.024	0.000	89.50	0.97
IPCWuL1*	-0.062	0.001	0.012	0.001	0.023	0.001	0.026	0.001	0.024	0.000	92.10	0.85
IPCWsL1*	-0.063	0.001	0.012	0.001	0.023	0.001	0.026	0.001	0.024	0.000	92.30	0.84
IPCWuL1L2	-0.074	0.001	0.000	0.001	0.027	0.001	0.027	0.001	0.028	0.001	94.60	0.71
IPCWsL1L2	-0.075	0.001	-0.000	0.001	0.027	0.001	0.027	0.001	0.028	0.001	94.70	0.71
IPCWuL1L2L3	-0.074	0.001	0.000	0.001	0.027	0.001	0.027	0.001	0.028	0.001	94.50	0.72
IPCWsL1L2L3	-0.075	0.001	-0.000	0.001	0.027	0.001	0.027	0.001	0.028	0.001	94.60	0.71
IPCWuL1L2L4	-0.074	0.001	0.001	0.001	0.027	0.001	0.027	0.001	0.028	0.001	94.70	0.71
IPCWsL1L2L4	-0.075	0.001	-0.000	0.001	0.027	0.001	0.027	0.001	0.028	0.001	94.80	0.70
IPCWuL1L2L4L5	-0.073	0.001	0.001	0.001	0.026	0.001	0.026	0.001	0.027	0.000	94.70	0.71
IPCWsL1L2L4L5	-0.074	0.001	0.000	0.001	0.026	0.001	0.026	0.001	0.027	0.000	95.00	0.69
IPCWuL1L2L3L4L5	-0.073	0.001	0.001	0.001	0.026	0.001	0.026	0.001	0.027	0.000	94.60	0.71
IPCWsL1L2L3L4L5	-0.074	0.001	0.000	0.001	0.026	0.001	0.026	0.001	0.027	0.000	94.80	0.70
IPCWuL1*L2*	-0.065	0.001	0.009	0.001	0.023	0.001	0.025	0.001	0.024	0.000	93.10	0.80
IPCWsL1*L2*	-0.066	0.001	0.008	0.001	0.023	0.001	0.025	0.001	0.024	0.000	93.40	0.79

Table C25. Table of simsum results for scenario 17

	Mean Estimate	MCSE	Bias	MCSE	EmpSE	MCSE	RMSE	MCSE	ModSE	MCSE	Coverage(%)	MCSE
ITT	-0.067	0.002	0.012	0.002	0.050	0.001	0.052	0.001	0.050	0.000	93.99	0.75
PP	-0.058	0.002	0.021	0.002	0.054	0.001	0.058	0.001	0.053	0.000	91.50	0.88
IPCWuL2	-0.071	0.002	0.008	0.002	0.065	0.001	0.065	0.003	0.060	0.001	92.10	0.85
IPCWsL2	-0.072	0.002	0.007	0.002	0.065	0.001	0.065	0.003	0.060	0.001	92.60	0.83
IPCWuL1	-0.070	0.002	0.010	0.002	0.061	0.001	0.062	0.002	0.059	0.001	92.80	0.82
IPCWsL1	-0.070	0.002	0.009	0.002	0.061	0.001	0.062	0.002	0.060	0.001	92.99	0.81
IPCWuL1L3	-0.069	0.002	0.010	0.002	0.060	0.001	0.061	0.001	0.059	0.001	93.00	0.81
IPCWsL1L3	-0.070	0.002	0.009	0.002	0.060	0.001	0.061	0.001	0.059	0.001	93.10	0.80
IPCWuL1L4	-0.070	0.002	0.009	0.002	0.062	0.001	0.063	0.002	0.060	0.001	93.20	0.80
IPCWsL1L4	-0.071	0.002	0.008	0.002	0.063	0.001	0.063	0.002	0.060	0.001	93.19	0.80
IPCWuL1L4L5	-0.069	0.002	0.010	0.002	0.061	0.001	0.062	0.001	0.060	0.001	93.09	0.80
IPCWsL1L4L5	-0.070	0.002	0.009	0.002	0.061	0.001	0.062	0.001	0.060	0.001	93.09	0.80
IPCWuL1L3L4L5	-0.069	0.002	0.010	0.002	0.061	0.001	0.061	0.001	0.059	0.001	93.20	0.80
IPCWsL1L3L4L5	-0.070	0.002	0.009	0.002	0.061	0.001	0.062	0.001	0.060	0.001	93.30	0.79
IPCWuLb1Lb2	-0.062	0.002	0.017	0.002	0.059	0.001	0.061	0.001	0.057	0.001	91.90	0.86
IPCWsLb1Lb2	-0.063	0.002	0.016	0.002	0.059	0.001	0.061	0.001	0.057	0.001	92.20	0.85
IPCWuL1*	-0.065	0.002	0.015	0.002	0.060	0.001	0.061	0.002	0.058	0.001	92.38	0.84
IPCWsL1*	-0.066	0.002	0.014	0.002	0.060	0.001	0.061	0.002	0.058	0.001	92.40	0.84
IPCWuL1L2	-0.075	0.002	0.004	0.002	0.063	0.001	0.063	0.002	0.061	0.001	93.20	0.80
IPCWsL1L2	-0.076	0.002	0.003	0.002	0.063	0.001	0.063	0.002	0.062	0.001	93.40	0.79
IPCWuL1L2L3	-0.075	0.002	0.005	0.002	0.063	0.001	0.063	0.002	0.061	0.001	93.30	0.79
IPCWsL1L2L3	-0.076	0.002	0.004	0.002	0.063	0.001	0.063	0.002	0.061	0.001	93.40	0.79
IPCWuL1L2L4	-0.076	0.002	0.004	0.002	0.065	0.001	0.065	0.002	0.062	0.001	93.30	0.79
IPCWsL1L2L4	-0.077	0.002	0.003	0.002	0.065	0.001	0.065	0.002	0.063	0.001	93.30	0.79
IPCWuL1L2L4L5	-0.076	0.002	0.004	0.002	0.065	0.001	0.065	0.002	0.062	0.001	93.80	0.76
IPCWsL1L2L4L5	-0.077	0.002	0.003	0.002	0.065	0.001	0.065	0.002	0.062	0.001	93.60	0.77
IPCWuL1L2L3L4L5	-0.075	0.002	0.004	0.002	0.065	0.001	0.065	0.002	0.062	0.001	93.50	0.78
IPCWsL1L2L3L4L5	-0.076	0.002	0.003	0.002	0.065	0.001	0.065	0.002	0.062	0.001	93.39	0.79
IPCWuL1*L2*	-0.068	0.002	0.011	0.002	0.061	0.001	0.061	0.002	0.059	0.001	92.80	0.82
IPCWsL1*L2*	-0.069	0.002	0.010	0.002	0.061	0.001	0.062	0.002	0.059	0.001	92.80	0.82

Table C26. Table of simsum results for scenario 18

	Mean Estimate	MCSE	Bias	MCSE	EmpSE	MCSE	RMSE	MCSE	ModSE	MCSE	Coverage(%)	MCSE
ITT	-0.066	0.001	0.013	0.001	0.030	0.001	0.033	0.001	0.032	0.000	93.00	0.81
PP	-0.061	0.001	0.018	0.001	0.032	0.001	0.037	0.001	0.034	0.000	92.50	0.83
IPCWuL2	-0.074	0.001	0.006	0.001	0.038	0.001	0.039	0.001	0.039	0.000	95.10	0.68
IPCWsL2	-0.075	0.001	0.005	0.001	0.038	0.001	0.038	0.001	0.039	0.001	95.30	0.67
IPCWuL1	-0.073	0.001	0.006	0.001	0.039	0.001	0.040	0.001	0.039	0.001	95.00	0.69
IPCWsL1	-0.074	0.001	0.006	0.001	0.039	0.001	0.040	0.001	0.039	0.001	95.00	0.69
IPCWuL1L3	-0.073	0.001	0.006	0.001	0.039	0.001	0.040	0.001	0.039	0.001	94.90	0.70
IPCWsL1L3	-0.074	0.001	0.006	0.001	0.039	0.001	0.040	0.001	0.039	0.001	95.20	0.68
IPCWuL1L4	-0.073	0.001	0.006	0.001	0.039	0.001	0.040	0.001	0.039	0.001	94.50	0.72
IPCWsL1L4	-0.074	0.001	0.006	0.001	0.039	0.001	0.040	0.001	0.039	0.001	94.70	0.71
IPCWuL1L4L5	-0.073	0.001	0.007	0.001	0.039	0.001	0.040	0.002	0.039	0.001	94.80	0.70
IPCWsL1L4L5	-0.073	0.001	0.006	0.001	0.039	0.001	0.040	0.002	0.039	0.001	95.00	0.69
IPCWuL1L3L4L5	-0.073	0.001	0.007	0.001	0.039	0.001	0.040	0.002	0.039	0.001	95.10	0.68
IPCWsL1L3L4L5	-0.074	0.001	0.006	0.001	0.039	0.001	0.040	0.002	0.039	0.001	95.20	0.68
IPCWuLb1Lb2	-0.065	0.001	0.014	0.001	0.035	0.001	0.038	0.001	0.036	0.000	92.79	0.82
IPCWsLb1Lb2	-0.066	0.001	0.014	0.001	0.035	0.001	0.038	0.001	0.036	0.000	93.10	0.80
IPCWuL1*	-0.067	0.001	0.012	0.001	0.036	0.001	0.038	0.001	0.037	0.000	93.60	0.77
IPCWsL1*	-0.068	0.001	0.011	0.001	0.036	0.001	0.038	0.001	0.037	0.000	94.00	0.75
IPCWuL1L2	-0.079	0.001	0.000	0.001	0.040	0.001	0.040	0.001	0.040	0.001	95.90	0.63
IPCWsL1L2	-0.080	0.001	-0.001	0.001	0.040	0.001	0.040	0.001	0.040	0.001	96.10	0.61
IPCWuL1L2L3	-0.079	0.001	0.000	0.001	0.040	0.001	0.040	0.001	0.040	0.001	95.80	0.63
IPCWsL1L2L3	-0.080	0.001	-0.000	0.001	0.040	0.001	0.040	0.001	0.040	0.001	96.20	0.60
IPCWuL1L2L4	-0.079	0.001	0.000	0.001	0.040	0.001	0.040	0.001	0.041	0.001	95.90	0.63
IPCWsL1L2L4	-0.080	0.001	-0.001	0.001	0.040	0.001	0.040	0.001	0.041	0.001	96.00	0.62
IPCWuL1L2L4L5	-0.079	0.001	0.001	0.001	0.040	0.001	0.040	0.002	0.041	0.001	95.70	0.64
IPCWsL1L2L4L5	-0.080	0.001	-0.000	0.001	0.040	0.001	0.040	0.002	0.041	0.001	95.70	0.64
IPCWuL1L2L3L4L5	-0.079	0.001	0.001	0.001	0.040	0.001	0.040	0.002	0.041	0.001	95.70	0.64
IPCWsL1L2L3L4L5	-0.080	0.001	-0.000	0.001	0.040	0.001	0.040	0.002	0.041	0.001	95.60	0.65
IPCWuL1*L2*	-0.071	0.001	0.008	0.001	0.036	0.001	0.037	0.001	0.037	0.000	94.50	0.72
IPCWsL1*L2*	-0.072	0.001	0.008	0.001	0.036	0.001	0.037	0.001	0.037	0.000	94.60	0.71

Table C27. Table of simsum results for scenario 19

	Mean Estimate	MCSE	Bias	MCSE	EmpSE	MCSE	RMSE	MCSE	ModSE	MCSE	Coverage(%)	MCSE
ITT	-0.099	0.001	-0.019	0.001	0.022	0.001	0.030	0.001	0.023	0.000	87.80	1.03
PP	-0.062	0.001	0.018	0.001	0.023	0.001	0.029	0.001	0.024	0.000	87.90	1.03
IPCWuL2	-0.074	0.001	0.005	0.001	0.027	0.001	0.028	0.001	0.028	0.000	94.00	0.75
IPCWsL2	-0.075	0.001	0.004	0.001	0.027	0.001	0.028	0.001	0.028	0.000	94.40	0.73
IPCWuL1	-0.073	0.001	0.006	0.001	0.027	0.001	0.028	0.001	0.027	0.000	93.60	0.77
IPCWsL1	-0.074	0.001	0.005	0.001	0.027	0.001	0.028	0.001	0.027	0.000	93.80	0.76
IPCWuL1L3	-0.073	0.001	0.006	0.001	0.027	0.001	0.028	0.001	0.027	0.000	93.60	0.77
IPCWsL1L3	-0.074	0.001	0.005	0.001	0.027	0.001	0.028	0.001	0.027	0.000	94.00	0.75
IPCWuL1L4	-0.073	0.001	0.007	0.001	0.027	0.001	0.028	0.001	0.027	0.000	93.20	0.80
IPCWsL1L4	-0.074	0.001	0.006	0.001	0.027	0.001	0.028	0.001	0.027	0.000	93.50	0.78
IPCWuL1L4L5	-0.072	0.001	0.007	0.001	0.027	0.001	0.028	0.001	0.027	0.000	93.00	0.81
IPCWsL1L4L5	-0.073	0.001	0.006	0.001	0.027	0.001	0.028	0.001	0.027	0.000	93.40	0.79
IPCWuL1L3L4L5	-0.073	0.001	0.007	0.001	0.027	0.001	0.028	0.001	0.027	0.000	93.20	0.80
IPCWsL1L3L4L5	-0.073	0.001	0.006	0.001	0.027	0.001	0.028	0.001	0.027	0.000	93.30	0.79
IPCWuLb1Lb2	-0.065	0.001	0.014	0.001	0.025	0.001	0.029	0.001	0.025	0.000	90.30	0.94
IPCWsLb1Lb2	-0.066	0.001	0.014	0.001	0.025	0.001	0.029	0.001	0.026	0.000	90.80	0.91
IPCWuL1*	-0.076	0.001	0.004	0.001	0.027	0.001	0.027	0.001	0.028	0.000	94.49	0.72
IPCWsL1*	-0.077	0.001	0.003	0.001	0.027	0.001	0.027	0.001	0.028	0.000	94.69	0.71
IPCWuL1L2	-0.080	0.001	-0.000	0.001	0.029	0.001	0.029	0.001	0.029	0.001	94.50	0.72
IPCWsL1L2	-0.080	0.001	-0.001	0.001	0.029	0.001	0.029	0.001	0.029	0.001	94.40	0.73
IPCWuL1L2L3	-0.079	0.001	-0.000	0.001	0.029	0.001	0.029	0.001	0.029	0.000	94.40	0.73
IPCWsL1L2L3	-0.080	0.001	-0.001	0.001	0.029	0.001	0.029	0.001	0.029	0.001	94.30	0.73
IPCWuL1L2L4	-0.079	0.001	-0.000	0.001	0.029	0.001	0.029	0.001	0.029	0.000	94.10	0.75
IPCWsL1L2L4	-0.080	0.001	-0.001	0.001	0.029	0.001	0.029	0.001	0.029	0.000	94.20	0.74
IPCWuL1L2L4L5	-0.079	0.001	0.001	0.001	0.028	0.001	0.028	0.001	0.029	0.000	94.30	0.73
IPCWsL1L2L4L5	-0.080	0.001	-0.000	0.001	0.028	0.001	0.028	0.001	0.029	0.000	94.20	0.74
IPCWuL1L2L3L4L5	-0.079	0.001	0.001	0.001	0.028	0.001	0.028	0.001	0.029	0.000	94.20	0.74
IPCWsL1L2L3L4L5	-0.080	0.001	-0.000	0.001	0.028	0.001	0.028	0.001	0.029	0.000	94.30	0.73
IPCWuL1*L2*	-0.083	0.001	-0.004	0.001	0.030	0.001	0.030	0.001	0.030	0.001	95.10	0.68
IPCWsL1*L2*	-0.084	0.001	-0.005	0.001	0.030	0.001	0.030	0.001	0.030	0.001	95.30	0.67

Table C28. Table of simsum results for scenario 20

	Mean Estimate	MCSE	Bias	MCSE	EmpSE	MCSE	RMSE	MCSE	ModSE	MCSE	Coverage(%)	MCSE
ITT	-0.090	0.001	-0.010	0.001	0.023	0.001	0.025	0.001	0.023	0.000	92.50	0.83
PP	-0.071	0.001	0.008	0.001	0.023	0.001	0.025	0.001	0.024	0.000	93.40	0.79
IPCWuL2	-0.077	0.001	0.002	0.001	0.029	0.001	0.029	0.001	0.029	0.000	95.10	0.68
IPCWsL2	-0.078	0.001	0.001	0.001	0.029	0.001	0.029	0.001	0.029	0.000	95.20	0.68
IPCWuL1	-0.077	0.001	0.003	0.001	0.028	0.001	0.028	0.001	0.029	0.000	95.20	0.68
IPCWsL1	-0.077	0.001	0.002	0.001	0.028	0.001	0.028	0.001	0.029	0.000	95.20	0.68
IPCWuL1L3	-0.077	0.001	0.003	0.001	0.028	0.001	0.028	0.001	0.029	0.000	95.40	0.66
IPCWsL1L3	-0.077	0.001	0.002	0.001	0.028	0.001	0.028	0.001	0.029	0.000	95.50	0.66
IPCWuL1L4	-0.076	0.001	0.003	0.001	0.028	0.001	0.028	0.001	0.029	0.000	95.30	0.67
IPCWsL1L4	-0.077	0.001	0.002	0.001	0.028	0.001	0.028	0.001	0.029	0.000	95.40	0.66
IPCWuL1L4L5	-0.076	0.001	0.003	0.001	0.028	0.001	0.028	0.001	0.028	0.000	94.90	0.70
IPCWsL1L4L5	-0.077	0.001	0.003	0.001	0.028	0.001	0.028	0.001	0.029	0.000	95.30	0.67
IPCWuL1L3L4L5	-0.076	0.001	0.003	0.001	0.028	0.001	0.028	0.001	0.029	0.000	95.10	0.68
IPCWsL1L3L4L5	-0.077	0.001	0.003	0.001	0.028	0.001	0.028	0.001	0.029	0.000	95.20	0.68
IPCWuLb1Lb2	-0.072	0.001	0.007	0.001	0.026	0.001	0.027	0.001	0.026	0.000	94.90	0.70
IPCWsLb1Lb2	-0.073	0.001	0.006	0.001	0.026	0.001	0.026	0.001	0.026	0.000	94.90	0.70
IPCWuL1*	-0.078	0.001	0.002	0.001	0.030	0.001	0.030	0.002	0.030	0.001	95.50	0.66
IPCWsL1*	-0.078	0.001	0.001	0.001	0.030	0.001	0.030	0.002	0.030	0.001	95.60	0.65
IPCWuL1L2	-0.080	0.001	-0.000	0.001	0.030	0.001	0.030	0.001	0.031	0.001	96.00	0.62
IPCWsL1L2	-0.081	0.001	-0.001	0.001	0.030	0.001	0.030	0.001	0.031	0.001	96.10	0.61
IPCWuL1L2L3	-0.080	0.001	-0.000	0.001	0.030	0.001	0.030	0.001	0.031	0.000	95.70	0.64
IPCWsL1L2L3	-0.081	0.001	-0.001	0.001	0.030	0.001	0.030	0.001	0.031	0.000	95.80	0.63
IPCWuL1L2L4	-0.080	0.001	-0.000	0.001	0.030	0.001	0.030	0.001	0.031	0.000	95.60	0.65
IPCWsL1L2L4	-0.080	0.001	-0.001	0.001	0.031	0.001	0.031	0.001	0.031	0.000	96.10	0.61
IPCWuL1L2L4L5	-0.079	0.001	0.000	0.001	0.030	0.001	0.030	0.001	0.030	0.000	95.30	0.67
IPCWsL1L2L4L5	-0.080	0.001	-0.001	0.001	0.030	0.001	0.030	0.001	0.031	0.000	95.70	0.64
IPCWuL1L2L3L4L5	-0.079	0.001	0.000	0.001	0.030	0.001	0.030	0.001	0.030	0.000	95.30	0.67
IPCWsL1L2L3L4L5	-0.080	0.001	-0.001	0.001	0.030	0.001	0.030	0.001	0.031	0.000	95.40	0.66
IPCWuL1*L2*	-0.081	0.001	-0.002	0.001	0.031	0.001	0.032	0.001	0.032	0.001	95.60	0.65
IPCWsL1*L2*	-0.082	0.001	-0.003	0.001	0.032	0.001	0.032	0.001	0.032	0.001	95.80	0.63

Table C29. Table of simsum results for scenario 21

	Mean Estimate	MCSE	Bias	MCSE	EmpSE	MCSE	RMSE	MCSE	ModSE	MCSE	Coverage(%)	MCSE
ITT	-0.103	0.001	-0.024	0.001	0.022	0.000	0.033	0.001	0.023	0.000	82.80	1.19
PP	-0.071	0.001	0.008	0.001	0.023	0.001	0.025	0.001	0.024	0.000	93.40	0.79
IPCWuL2	-0.077	0.001	0.002	0.001	0.029	0.001	0.029	0.001	0.029	0.000	95.10	0.68
IPCWsL2	-0.078	0.001	0.001	0.001	0.029	0.001	0.029	0.001	0.029	0.000	95.20	0.68
IPCWuL1	-0.077	0.001	0.003	0.001	0.028	0.001	0.028	0.001	0.029	0.000	95.20	0.68
IPCWsL1	-0.077	0.001	0.002	0.001	0.028	0.001	0.028	0.001	0.029	0.000	95.20	0.68
IPCWuL1L3	-0.077	0.001	0.003	0.001	0.028	0.001	0.028	0.001	0.029	0.000	95.40	0.66
IPCWsL1L3	-0.077	0.001	0.002	0.001	0.028	0.001	0.028	0.001	0.029	0.000	95.50	0.66
IPCWuL1L4	-0.076	0.001	0.003	0.001	0.028	0.001	0.028	0.001	0.029	0.000	95.30	0.67
IPCWsL1L4	-0.077	0.001	0.002	0.001	0.028	0.001	0.028	0.001	0.029	0.000	95.40	0.66
IPCWuL1L4L5	-0.076	0.001	0.003	0.001	0.028	0.001	0.028	0.001	0.028	0.000	94.90	0.70
IPCWsL1L4L5	-0.077	0.001	0.003	0.001	0.028	0.001	0.028	0.001	0.029	0.000	95.30	0.67
IPCWuL1L3L4L5	-0.076	0.001	0.003	0.001	0.028	0.001	0.028	0.001	0.029	0.000	95.10	0.68
IPCWsL1L3L4L5	-0.077	0.001	0.003	0.001	0.028	0.001	0.028	0.001	0.029	0.000	95.20	0.68
IPCWuLb1Lb2	-0.072	0.001	0.007	0.001	0.026	0.001	0.027	0.001	0.026	0.000	94.90	0.70
IPCWsLb1Lb2	-0.073	0.001	0.006	0.001	0.026	0.001	0.026	0.001	0.026	0.000	94.90	0.70
IPCWuL1*	-0.082	0.001	-0.003	0.001	0.029	0.001	0.029	0.002	0.029	0.001	95.60	0.65
IPCWsL1*	-0.083	0.001	-0.004	0.001	0.029	0.001	0.029	0.002	0.029	0.001	95.40	0.66
IPCWuL1L2	-0.080	0.001	-0.000	0.001	0.030	0.001	0.030	0.001	0.031	0.001	96.00	0.62
IPCWsL1L2	-0.081	0.001	-0.001	0.001	0.030	0.001	0.030	0.001	0.031	0.001	96.10	0.61
IPCWuL1L2L3	-0.080	0.001	-0.000	0.001	0.030	0.001	0.030	0.001	0.031	0.000	95.70	0.64
IPCWsL1L2L3	-0.081	0.001	-0.001	0.001	0.030	0.001	0.030	0.001	0.031	0.000	95.80	0.63
IPCWuL1L2L4	-0.080	0.001	-0.000	0.001	0.030	0.001	0.030	0.001	0.031	0.000	95.60	0.65
IPCWsL1L2L4	-0.080	0.001	-0.001	0.001	0.031	0.001	0.031	0.001	0.031	0.000	96.10	0.61
IPCWuL1L2L4L5	-0.079	0.001	0.000	0.001	0.030	0.001	0.030	0.001	0.030	0.000	95.30	0.67
IPCWsL1L2L4L5	-0.080	0.001	-0.001	0.001	0.030	0.001	0.030	0.001	0.031	0.000	95.70	0.64
IPCWuL1L2L3L4L5	-0.079	0.001	0.000	0.001	0.030	0.001	0.030	0.001	0.030	0.000	95.30	0.67
IPCWsL1L2L3L4L5	-0.080	0.001	-0.001	0.001	0.030	0.001	0.030	0.001	0.031	0.000	95.40	0.66
IPCWuL1*L2*	-0.087	0.001	-0.008	0.001	0.031	0.001	0.032	0.001	0.031	0.001	95.30	0.67
IPCWsL1*L2*	-0.088	0.001	-0.009	0.001	0.031	0.001	0.032	0.001	0.031	0.001	95.30	0.67

Table C30. Table of simsum results for scenario 22

	Mean Estimate	MCSE	Bias	MCSE	EmpSE	MCSE	RMSE	MCSE	ModSE	MCSE	Coverage(%)	MCSE
ITT	-0.058	0.001	0.021	0.001	0.022	0.000	0.030	0.001	0.023	0.000	85.10	1.13
PP	-0.071	0.001	0.008	0.001	0.023	0.001	0.025	0.001	0.024	0.000	93.40	0.79
IPCWuL2	-0.077	0.001	0.002	0.001	0.029	0.001	0.029	0.001	0.029	0.000	95.10	0.68
IPCWsL2	-0.078	0.001	0.001	0.001	0.029	0.001	0.029	0.001	0.029	0.000	95.20	0.68
IPCWuL1	-0.077	0.001	0.003	0.001	0.028	0.001	0.028	0.001	0.029	0.000	95.20	0.68
IPCWsL1	-0.077	0.001	0.002	0.001	0.028	0.001	0.028	0.001	0.029	0.000	95.20	0.68
IPCWuL1L3	-0.077	0.001	0.003	0.001	0.028	0.001	0.028	0.001	0.029	0.000	95.40	0.66
IPCWsL1L3	-0.077	0.001	0.002	0.001	0.028	0.001	0.028	0.001	0.029	0.000	95.50	0.66
IPCWuL1L4	-0.076	0.001	0.003	0.001	0.028	0.001	0.028	0.001	0.029	0.000	95.30	0.67
IPCWsL1L4	-0.077	0.001	0.002	0.001	0.028	0.001	0.028	0.001	0.029	0.000	95.40	0.66
IPCWuL1L4L5	-0.076	0.001	0.003	0.001	0.028	0.001	0.028	0.001	0.028	0.000	94.90	0.70
IPCWsL1L4L5	-0.077	0.001	0.003	0.001	0.028	0.001	0.028	0.001	0.029	0.000	95.30	0.67
IPCWuL1L3L4L5	-0.076	0.001	0.003	0.001	0.028	0.001	0.028	0.001	0.029	0.000	95.10	0.68
IPCWsL1L3L4L5	-0.077	0.001	0.003	0.001	0.028	0.001	0.028	0.001	0.029	0.000	95.20	0.68
IPCWuLb1Lb2	-0.072	0.001	0.007	0.001	0.026	0.001	0.027	0.001	0.026	0.000	94.90	0.70
IPCWsLb1Lb2	-0.073	0.001	0.006	0.001	0.026	0.001	0.026	0.001	0.026	0.000	94.90	0.70
IPCWuL1*	-0.069	0.001	0.010	0.001	0.027	0.001	0.028	0.001	0.027	0.000	93.80	0.76
IPCWsL1*	-0.070	0.001	0.009	0.001	0.027	0.001	0.028	0.001	0.027	0.000	94.10	0.75
IPCWuL1L2	-0.080	0.001	-0.000	0.001	0.030	0.001	0.030	0.001	0.031	0.001	96.00	0.62
IPCWsL1L2	-0.081	0.001	-0.001	0.001	0.030	0.001	0.030	0.001	0.031	0.001	96.10	0.61
IPCWuL1L2L3	-0.080	0.001	-0.000	0.001	0.030	0.001	0.030	0.001	0.031	0.000	95.70	0.64
IPCWsL1L2L3	-0.081	0.001	-0.001	0.001	0.030	0.001	0.030	0.001	0.031	0.000	95.80	0.63
IPCWuL1L2L4	-0.080	0.001	-0.000	0.001	0.030	0.001	0.030	0.001	0.031	0.000	95.60	0.65
IPCWsL1L2L4	-0.080	0.001	-0.001	0.001	0.031	0.001	0.031	0.001	0.031	0.000	96.10	0.61
IPCWuL1L2L4L5	-0.079	0.001	0.000	0.001	0.030	0.001	0.030	0.001	0.030	0.000	95.30	0.67
IPCWsL1L2L4L5	-0.080	0.001	-0.001	0.001	0.030	0.001	0.030	0.001	0.031	0.000	95.70	0.64
IPCWuL1L2L3L4L5	-0.079	0.001	0.000	0.001	0.030	0.001	0.030	0.001	0.030	0.000	95.30	0.67
IPCWsL1L2L3L4L5	-0.080	0.001	-0.001	0.001	0.030	0.001	0.030	0.001	0.031	0.000	95.40	0.66
IPCWuL1*L2*	-0.070	0.001	0.010	0.001	0.028	0.001	0.029	0.001	0.028	0.000	94.20	0.74
IPCWsL1*L2*	-0.070	0.001	0.009	0.001	0.028	0.001	0.029	0.001	0.029	0.000	94.20	0.74

Appendix D Supplementary information for the case study

D.1 Analysis details

In ODYSSEY, 707 participants were randomised and they were followed at weeks 4, 12 and then every 12 weeks. The primary endpoint was treatment failure by 96 weeks and the non-inferiority margin was specified to be 0.10 on the risk difference scale. The primary analysis was done by comparing the cumulative risk of treatment failure at 96 weeks between randomised groups following the ITT principle. PP excluding patients with major violation of the eligibility criteria and censoring by deviation from treatment strategies was done as supplementary analysis.

We implemented the outcome modelling and analysis as in the published ODYSSEY main paper. In the main paper, the time to the first event was compared between groups with the use of Cox regression. The cumulative treatment-failure function for each randomised group was estimated as a weighted mean of the corresponding stratum-specific cumulative treatment-failure functions (estimated from a Cox model with adjustment for stratification factors and randomised group), with weights proportional to the number of participants in each stratum at baseline. The estimated probability of treatment failure by week 96 was compared between the treatment groups. The 95% bias-corrected confidence intervals were estimated with the use of the bootstrap method with 1000 samples.

Three covariates which are stratification factors were adjusted: previous experience of antiretroviral treatment, site having routine availability vs. non availability of resistance tests for children failing treatment, and pre-specified nucleoside (or nucleotide) reverse-transcriptase inhibitor (NRTI) backbone.

We implemented both IPCW with unstabilised weights and IPCW stabilising for time.

D.2 Sensitivity analysis on a potential confounder

Table D1. Results of the sensitivity analysis with additional covariates added into the weighting model.

Covariate added[a]	Estimator	Estimate [b]		
		EST	SE	95% CI[c]
Baseline weight	IPCW1u	-0.061	0.033	(-0.118, 0.013)
	IPCW1s	-0.061	0.033	(-0.116, 0.010)
	IPCW2u	-0.061	0.033	(-0.118, 0.011)
	IPCW2s	-0.061	0.032	(-0.117, 0.007)
Baseline BMI	IPCW1u	-0.061	0.033	(-0.118, 0.012)
	IPCW1s	-0.061	0.032	(-0.116, 0.011)
	IPCW2u	-0.061	0.033	(-0.117, 0.012)
	IPCW2s	-0.061	0.032	(-0.116, 0.009)

Note: EST = Point estimate; SE = Standard error; 95% CI = 95% Confidence interval; IPCW1u = IPCW with unstabilised weight for ICE 2; IPCW1s = IPCW with stabilised weight for ICE 2; IPCW2u = IPCW with unstabilised weight for ICE 1 and ICE 2; IPCW2s = IPCW with stabilised weight for ICE 1 and ICE 2; BMI = Body mass index.
[a] Analysis in the main text included baseline covariates (age, sex and WHO performance stage) and time-varying covariates (CD4 count, CD4/CD8 ratio and viral load). Here we explore adding two baseline covariates which are predictors of the outcome.
[b] Pre-specified NI margin: 0.100.
